# Breed-Specific Hematological Phenotypes in the Dog: A Natural Resource for the Genetic Dissection of Hematological Parameters in a Mammalian Species

**DOI:** 10.1371/journal.pone.0081288

**Published:** 2013-11-25

**Authors:** Jennifer Lawrence, Yu-Mei Ruby Chang, Balazs Szladovits, Lucy J. Davison, Oliver A. Garden

**Affiliations:** 1 Department of Clinical Sciences and Services, Regulatory T Cell Laboratory, The Royal Veterinary College, Camden Campus, Royal College Street, London, United Kingdom; 2 Research Office, The Royal Veterinary College, Camden Campus, Royal College Street, London, United Kingdom; 3 Department of Pathology and Pathogen Biology, The Royal Veterinary College, Hawkshead Campus, North Mymms, Hatfield, Hertfordshire, United Kingdom; 4 Henry Wellcome Building, Centre for Cellular and Molecular Physiology, University of Oxford, Roosevelt Drive, Headington, Oxford, United Kingdom; 5 Department of Veterinary Medicine, University of Cambridge, Cambridge, United Kingdom; Auburn University, United States of America

## Abstract

Remarkably little has been published on hematological phenotypes of the domestic dog, the most polymorphic species on the planet. Information on the signalment and complete blood cell count of all dogs with normal red and white blood cell parameters judged by existing reference intervals was extracted from a veterinary database. Normal hematological profiles were available for 6046 dogs, 5447 of which also had machine platelet concentrations within the reference interval. Seventy-five pure breeds plus a mixed breed control group were represented by 10 or more dogs. All measured parameters except mean corpuscular hemoglobin concentration (MCHC) varied with age. Concentrations of white blood cells (WBCs), neutrophils, monocytes, lymphocytes, eosinophils and platelets, but not red blood cell parameters, all varied with sex. Neutering status had an impact on hemoglobin concentration, mean corpuscular hemoglobin (MCH), MCHC, and concentrations of WBCs, neutrophils, monocytes, lymphocytes and platelets. Principal component analysis of hematological data revealed 37 pure breeds with distinctive phenotypes. Furthermore, all hematological parameters except MCHC showed significant differences between specific individual breeds and the mixed breed group. Twenty-nine breeds had distinctive phenotypes when assessed in this way, of which 19 had already been identified by principal component analysis. Tentative breed-specific reference intervals were generated for breeds with a distinctive phenotype identified by comparative analysis. This study represents the first large-scale analysis of hematological phenotypes in the dog and underlines the important potential of this species in the elucidation of genetic determinants of hematological traits, triangulating phenotype, breed and genetic predisposition.

## Introduction

The remarkable steady-state constancy of numbers of peripheral red and white blood cells reflects a network of homeostatic mechanisms that retain the *status quo* in health [1-3]. Aberrations of cell numbers may be implicated in immunodeficiency, autoimmune disease and hemolymphatic malignancies [4,5], and the complete blood cell count (CBC) is a crucial diagnostic and screening tool in medical practice. Recent genome-wide association studies (GWAS) of healthy humans have shown that a number of hematological parameters are associated with key genetic loci, suggesting novel regulatory pathways and candidate causative genes in hemolymphatic diseases [6,7]. Similar genetic constraints on peripheral blood lineages have also been discovered in laboratory mice [8-10], including differences in numbers of peripheral regulatory T cells (Tregs) [11]. 

The dog is the most polymorphic terrestrial mammal on the planet. The creation of distinct breeds has introduced remarkable genetic homogeneity, providing an unparalleled opportunity to dissect the genetic basis of complex traits: studies that would require thousands of human subjects typically require fewer than 100 in the dog [12,13], which is rapidly gaining traction as a model species in a number of biomedical disciplines. Certain hematological phenotypes have been recognized in dogs for many years [14-21], but no large-scale, systematic studies of the phenotypic diversity of peripheral blood in this species have been undertaken. Our objective was therefore to undertake a comparative analysis of normal hematological profiles within a large veterinary database in order to identify possible breed-specific phenotypes as a prelude to genetic analysis of these traits in the future. Two complementary analytical approaches identified a number of unique hematological phenotypes in this species. 

## Materials and Methods

(Detailed protocols may be found in the Materials & Methods S1; a summary of methods is presented here.)

### Inclusion and exclusion criteria

Hematological profiles were collected from the database of the Royal Veterinary College (RVC) Diagnostic Laboratory (DL) from December 1998 to August 2012, comparing each dog’s profile to the generic reference intervals used by the DL for all dogs other than greyhounds [22]. In the case of greyhounds, comparisons were made to widely-applied greyhound-specific reference intervals. Hematological profiles were included in the study if every parameter fell within the reference interval, excluding platelet concentrations which were considered separately. Platelet concentrations falling outside the reference interval were not considered as an exclusion criterion for the interpretation of the other hematological parameters, but only those platelet concentrations falling within the reference interval were considered in the analysis of platelets. Data were collected from both pure breed dogs and a control group of mixed breed dogs, anonymizing all patient details in accordance with general guidelines laid down by the Royal Veterinary College Ethics and Welfare Committee. 

### Specimen collection, analysis, and quality control

All blood samples were collected by licensed veterinarians for routine diagnostic purposes under the Veterinary Surgeons Act (1966), following written informed consent by the owners of the dogs. Blood was analyzed using a CELL-DYN 3500 Hematology Analyzer (Abbott Laboratories Ltd, Maidenhead; UK). Blood smears were evaluated under the direct supervision of a Board-certified veterinary clinical pathologist in every case. Reported parameters were red blood cell (RBC) concentration, mean corpuscular volume (MCV), hematocrit (Hct), hemoglobin (Hb) concentration, mean corpuscular hemoglobin (MCH), mean corpuscular hemoglobin concentration (MCHC), and concentrations of white blood cells (WBCs), neutrophils, monocytes, lymphocytes, eosinophils, and platelets. In the case of WBCs, at least a 100-cell manual differential count was undertaken as a basis for the calculation of the concentrations of neutrophils, monocytes, lymphocytes and eosinophils.

### Statistical analysis

Age of the dogs was classified into eight mutually exclusive categories: less than or equal to one year of age (designated ≤1), greater than one year to less than or equal to two years of age (designated >1:≤2), and >2:≤4, >4:≤6, >6:≤8, >8:≤10, >10:≤12, and >12 years of age. (For clarity of expression in the text of the Results and Discussion sections, these age categories are respectively labeled ≤1, 1‒2, 2‒4, 4‒6, 6‒8, 8‒10, 10‒12 and >12 years.) A linear mixed effects model was used to assess the effect of age, sex, neutering status, and all two-way and three-way interactions on each parameter, taking breed as a random effect. Significance was assumed when p<0.05. Residuals were defined as the observed values minus the estimated fixed effects of age, sex, and neutering status. For those breeds represented by at least 10 dogs, principal component analysis (PCA) was undertaken on all but the mixed breed dogs. Furthermore, for each hematological parameter the distributions of residuals for all breeds represented by at least 10 dogs were compared with those of the mixed breed dogs by two-sample Kolmogorov-Smirnov (KS) tests. All breeds were classified into groups previously defined by haplotype analysis of single nucleotide polymorphisms (SNPs) [23], or to their closest matches (Tables 1 to 12). Those breeds not identified in this haplotype analysis and without an obvious nearest match were considered in a miscellaneous group labeled ‘Other’. Where significant differences were identified for breeds with at least 120 individuals, tentative breed-specific reference intervals were calculated as the estimated 2.5% and 97.5% of the residuals, plus the appropriate adjusted means accounting for age, sex and neutering status. 

## Results

### Study population

 Of 7281 normal hematological profiles identified in the study period, complete information was available for 6046 dogs, 5447 of which had platelet concentrations within the reference interval. The study population included 877 intact females, 1665 neutered females, 1670 intact males and 1834 neutered males. Dogs ranged in age from eight weeks to 23 years. Seventy-five breeds plus the mixed breed control group included 10 or more individuals, for which descriptive statistics are represented in Tables 1 to 12. Thirteen breeds plus the mixed breed group included 120 or more individuals, including – in descending popularity – the Labrador retriever (n=761), German shepherd dog (GSD; n=346), boxer (n=351), Cavalier King Charles spaniel (CKCS; n=280), cocker spaniel (n=227), West Highland white terrier (WHWT; n=199), Jack Russell terrier (JRT; n=180), golden retriever (n=171), springer spaniel (n=168), Staffordshire bull terrier (SBT; n=165), Yorkshire terrier (n=154), Border collie (n=146) and Rottweiler (n=128). 

### Effects of age, sex and neutering status

**Figure 1:**
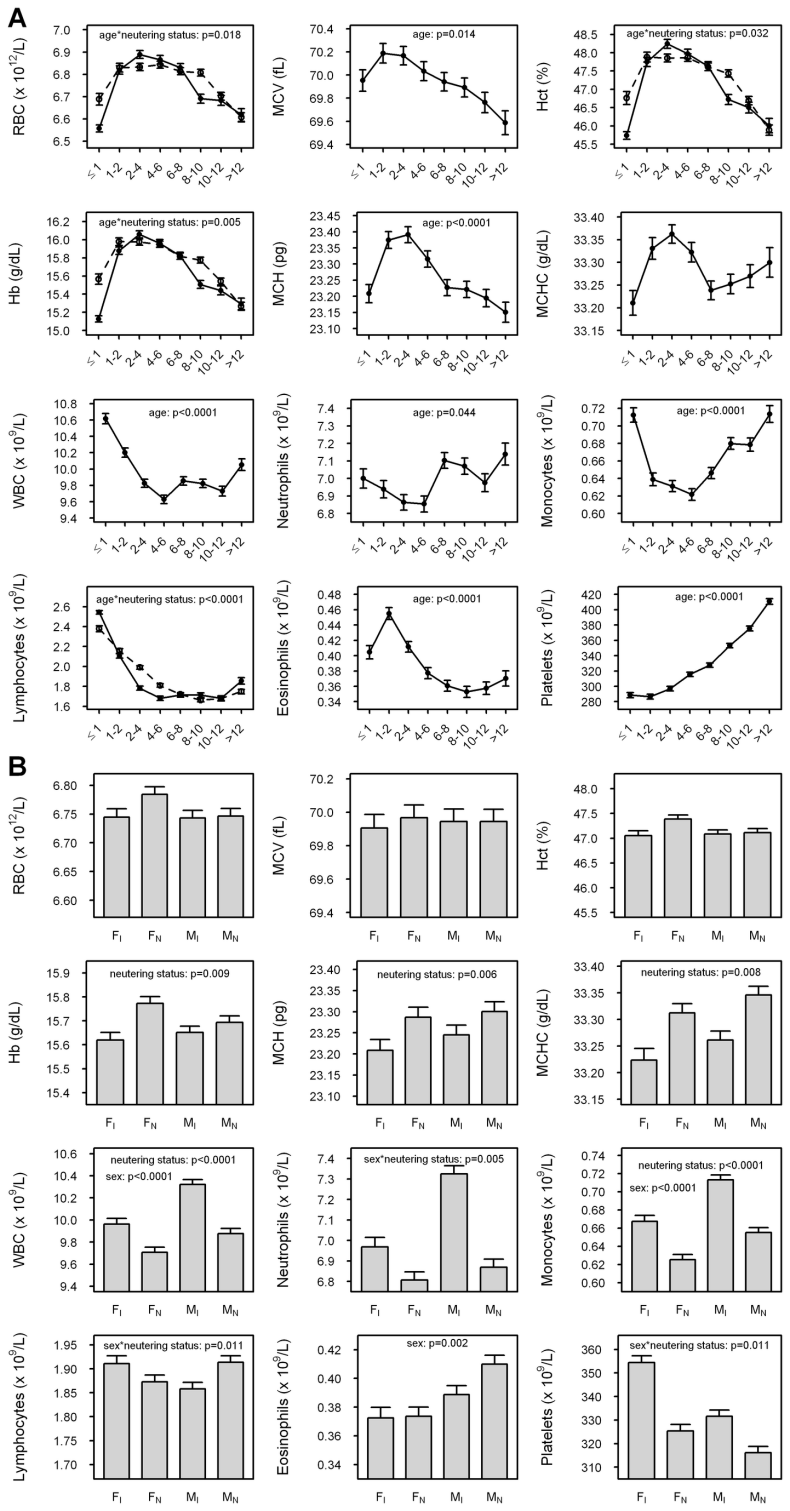
Effects of age, sex and neutering status on hematological parameters. (a) Age: The adjusted mean values – accounting for sex, breed and neutering status – for each of the 12 hematological parameters are represented on the respective *y* axes, showing age in years on the x axes. All measurands except MCHC (p=0.24) varied with age and in the case of RBC concentration, Hct, Hb and lymphocyte concentration there was an interaction between age and neutering status; in these cases, values for the intact dogs (solid lines) were distinguished from those for the neutered dogs (interrupted lines) in the plots shown. (b) Sex and neutering status: The adjusted mean values – accounting for breed and age – for each of the 12 hematological parameters are represented on the respective *y* axes, showing sex (F=female; M=male) and neutering status (I=intact; N=neutered) on the x axes. When present, significant differences (in sex, neutering status, or sex*neutering status interaction) are shown at the top of the figure; in some cases, only the interaction between sex and neutering status was significant. These analyses were all undertaken on the complete dataset of dogs, including both mixed and pure breeds.

All measured parameters except MCHC (p=0.24) varied with age (p<0.05; Figure 1A, Table S13). Red cell measurands generally increased to a maximum value at 1‒2 or 2‒4 years of age and then decreased with advancing age, while white cell measurands showed a variable response: WBC and monocyte concentrations decreased from ≤1 to 4‒6 years of age and then increased again to values similar to those of the dogs ≤1 year of age (monocytes) or showed only a modest increase (WBCs); concentrations of neutrophils appeared to peak at 6‒8 and >12 years of age, but the differences between age groups were generally small; lymphocyte concentrations showed a progressive decrease from ≤1 to 10‒12 years of age and then marginally increased to >12 years of age; and eosinophil concentrations showed a peak at 1‒2 years of age and then decreased to a plateau from 6‒8 to >12 years of age, arriving at a value lower than that of dogs ≤1 year of age. Platelet concentrations progressively increased from 1‒2 to >12 years of age. There were no differences in red cell measurands between the sexes, but concentrations of WBCs, neutrophils, monocytes, lymphocytes, eosinophils and platelets all varied with sex (p<0.05; Figure 1B, Table S13). There were lower mean concentrations of WBCs, neutrophils, monocytes and eosinophils, but higher mean concentrations of platelets, in female than male dogs. By comparison, neutering status had an impact on Hb concentration, MCH, MCHC, and concentrations of WBCs, neutrophils, monocytes, lymphocytes and platelets (p<0.05; Figure 1B, Table S13). In the case of Hb concentration, MCH and MCHC, neutering increased the mean value for each of the parameters in both sexes, whereas for concentrations of WBCs, neutrophils, monocytes and platelets neutering decreased mean values of the parameters (Figure 1B). There were no significant interactions between age and sex for any of the parameters, but significant interactions between sex and neutering status for neutrophil (p=0.005), lymphocyte (p=0.011) and platelet (p=0.011) concentrations (Figure 1B). For neutrophils, adjusted mean concentrations of intact male dogs were 0.45±0.97 x 10^9^/L (±SEM) higher than those of neutered male dogs (p<0.0001), those of intact female dogs were 0.36±0.08 x 10^9^/L lower than those of intact male dogs, and there were no significant differences between intact and neutered female dogs (p=0.061) or neutered male and female dogs (p=0.333); for lymphocytes, adjusted mean concentrations of intact male dogs were 0.06±0.02 x 10^9^/L lower than those of neutered male dogs (p=0.021), but there were no differences between concentrations of intact and neutered female dogs (p=0.207), intact male and female dogs (p=0.064), or neutered male and female dogs (p=0.074); and for platelets, adjusted mean concentrations of intact male dogs were 15.5±3.5 x 10^9^/L higher than those of neutered male dogs (p<0.0001), those of intact female dogs were 29.0±4.4 x 10^9^/L higher than those of neutered female dogs (p<0.0001), those of intact female dogs were 22.8±4.1 x 10^9^/L higher than those of intact male dogs (p<0.0001), and those of neutered female dogs were 9.28±3.28 x 10^9^/L higher than those of neutered male dogs (p=0.005). There were significant interactions between age and neutering status for RBC concentrations (p=0.018), Hb concentration (p=0.005), Hct (p=0.032) and lymphocyte concentrations (p<0.0001): in all cases, the patterns of change were similar but more exaggerated in the intact compared to the neutered dogs (Figure 1A). 

The observations recorded in this section relate to the complete dataset of dogs, including both mixed and pure breeds. However, broadly similar trends were observed when the three most populous breeds – the Labrador retriever (n=761), GSD (n=346) and boxer (n=351) – were examined independently (Figures S1 to S3). 

### Principal component analysis identifies breed-specific hematological phenotypes

**Figure 2:**
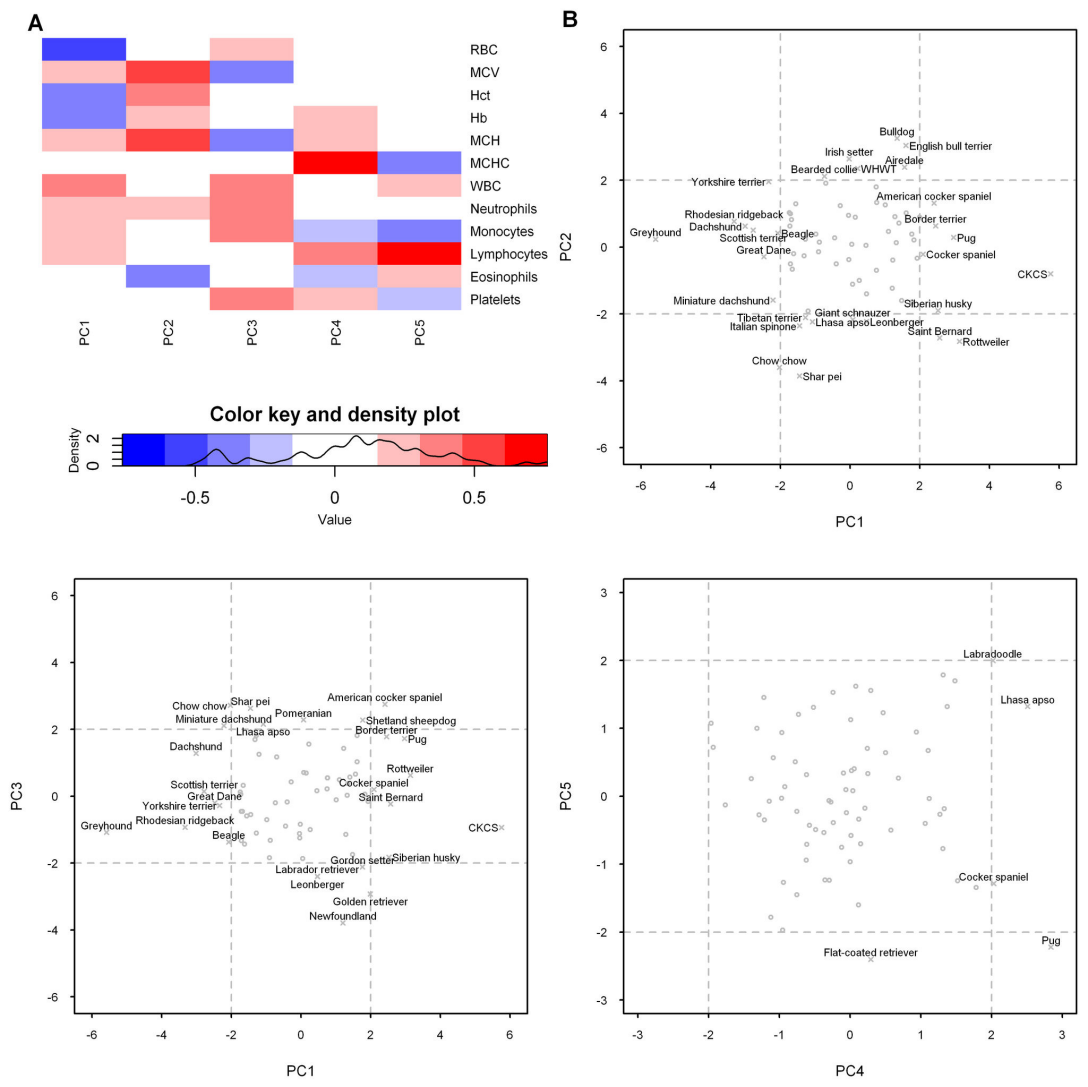
Principal component analysis identifies breed-specific hematological phenotypes. Of the 11 principal components identified in the current dataset, the first five had Eigenvalues of over 1 and cumulatively accounted for 82% of the total variance. A heatmap showing correlations between the hematological parameters and the first five principal components (PC1 to PC5) is shown in part (a). Biplots (PC1 *vs* PC2; PC1 *vs* PC3; PC4 *vs* PC5) are shown in part (b), revealing outlying breeds with principal components of greater than +2 and less than -2, representing distinctive phenotypes. These outlying breeds are labeled in the biplots.

 Eleven principal components were identified in the current dataset, of which the first five had Eigenvalues of greater than 1 (Figure 2A), cumulatively accounting for 82% of the total variance of the dataset (Table S14). However, the first three principal components identified the majority of breeds with distinctive phenotypes, defined by at least one principal component of less than -2 or greater than +2; the inclusion of principal components 4 and 5 added only the Labradoodle and flat-coated retriever to this list. Thus, a total of 37 of 75 breeds showed distinctive phenotypes on the basis of the first five principal components (Figure 2B). There were 10 breeds with distinctive phenotypes on the basis of principal component 1, eight on the basis of principal component 2, two on the basis of principal components 1 and 2, six on the basis of principal component 3, two on the basis of principal components 2 and 3, and two on the basis of principal components 1 and 3 (Figure 2B). The chow chow had a distinctive phenotype on the basis of principal components 1, 2 and 4; the Lhasa apso, on the basis of principal components 2, 3 and 4; and the cocker spaniel, on the basis of principal components 1 and 4. Finally, the Labradoodle had a distinctive phenotype on the basis of principal component 4, while the flat-coated retriever had a distinctive phenotype on the basis of principal component 5; only the pug had a distinctive phenotype on the basis of principal components 1, 4 and 5. 

Having identified a number of breeds with ‘outlying’ hematological characteristics by PCA, we undertook a complementary analytical approach by comparing all of the individual breeds with the mixed breed (control) group, parameter by parameter, reasoning that those inbred dogs with a distinctive phenotype should have significantly different distributions of hematological measurands from outbred dogs with a heterogeneous genetic background. 

### Comparisons with a control group of mixed breed dogs confirm breed-specific phenotypes

**Figure 3:**
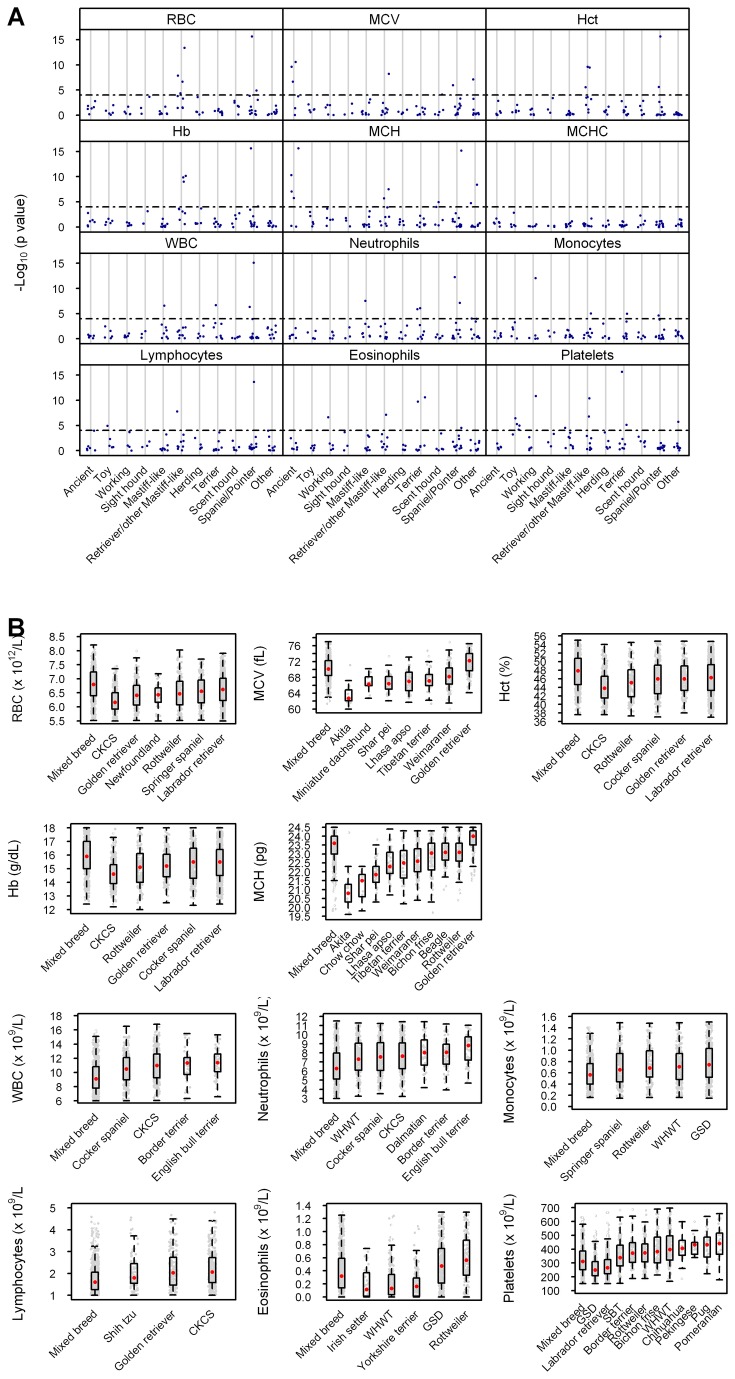
Pairwise comparisons of hematological parameters between pure breed and mixed breed dogs confirm breed-specific phenotypes. The results of two-sample Kolmogorov-Smirnov tests to compare the distributions of residuals for the pure breed *versus* mixed breed dogs are shown in part (a). (Residuals were defined as the observed values minus the estimated fixed effects of age, sex and neutering status.) Respective *y* axes show -log_10_(p value) of specific individual breeds assigned to one of 11 genetically-related groups, shown on the x axes. A Bonferroni correction yielded a threshold for significance of 10^-4^, represented by the interrupted line. All breeds with residual values significantly different from those of the mixed breed population are shown above the line. Combined box-and-whisker / dot plots representing hematological data from breeds showing a significant difference from the mixed breed group are shown in part (b) for each of the 11 parameters for which differences were documented. Each small dot represents an individual dog, the boxes show the respective 25^th^ and 75^th^ percentiles, the larger red dots median values, and the whiskers the lowest and highest data points still within 1.5 times the interquartile range of the respective lower and upper quartiles.

 All hematological parameters except MCHC showed significant differences between specific individual breeds and the mixed breed group (Figure 3; Table S15). Notable observations included the differences between pure and mixed breed dogs in RBC concentration, Hct and Hb concentration in members of the Retriever/other Mastiff-like group (golden and Labrador retrievers, and Rottweiler) and members of the Spaniel/Pointer group (CKCS, cocker and springer spaniels); differences in MCV and MCH in members of the Ancient group (shar pei, Akita and Tibetan terrier) and individual members of the Retriever/other Mastiff-like (golden retriever), Spaniel/Pointer (weimaraner) and Other (Lhasa apso) groups; differences in WBC and neutrophil concentrations in members of the Spaniel/Pointer (CKCS, cocker spaniel), Terrier (WHWT, Border terrier) and Mastiff-like (English bulldog) groups; differences in monocyte concentrations in members of the Working (German shepherd dog; GSD), Retriever/other Mastiff-like (Rottweiler), Terrier (WHWT) and Spaniel/Pointer (springer spaniel) groups; differences in lymphocyte concentrations in members of the Spaniel/Pointer (CKCS), Retriever/other Mastiff-like (golden retriever) and Toy (shih tzu) groups; differences in eosinophil concentrations in members of the Terrier (WHWT, Yorkshire terrier), Working (GSD), Retriever/other Mastiff-like (Rottweiler) and Spaniel/Pointer (Irish setter) groups; and differences in platelet concentrations in members of the Toy (Pomeranian, pug, Pekingese and Chihuahua), Retriever/other Mastiff-like (Rottweiler, Labrador retriever) and Terrier (WHWT, Border terrier) groups, as well as individual members of the Working (GSD), Mastiff-like (SBT) and Other (bichon frise) groups (Figure 3A,B). Breeds with multiple hematological differences from the mixed breed group, spanning both red and white cell parameters and, or platelet concentrations, were the CKCS (RBC, Hct, Hb, WBC, neutrophils, lymphocytes); golden retriever (RBC, MCV, Hct, Hb, MCHC, lymphocytes) and Labrador retriever (RBC, Hct, Hb, platelets); springer spaniel (RBC, monocytes) and cocker spaniel (Hct, Hb, WBC, neutrophils); Rottweiler (RBC, Hb, MCH, monocytes, eosinophils, platelets); WHWT (monocytes, eosinophils, platelets) and Border terrier (WBC, neutrophils, platelets); and GSD (monocytes, eosinophils, platelets). 

Figure S4 summarizes the results of PCA and pairwise comparisons with the mixed breed group, showing that the majority of breeds highlighted by comparative analysis (19/29) were also identified by PCA but that almost 50% (18/37) of the breeds highlighted by PCA were identified by this analytical approach alone. 

### Towards breed-specific hematological reference intervals

Of the 29 breeds identified by pairwise comparisons with the mixed breed group, 11 were represented by at least 120 dogs (Labrador retriever, GSD, cocker spaniel, WHWT, JRT, golden retriever, springer spaniel, SBT, Yorkshire terrier, Border collie and Rottweiler). Histograms of the hematological profiles and tentative breed-specific reference intervals, adjusted for sex, neutering status and age, were calculated for these breeds (Figures S5 to S15; Tables S16 to S26). Upper limits for MCH were not calculated for any of the breeds owing to our concern for data truncation: 3.2% of individuals had an MCH equal to the existing RVC DL upper reference limit of 24.5pg. 

## Discussion

 The current study adds to the growing body of literature on the phenotypic diversity of the domestic dog [24], examining hematological data from over 6000 individuals representing 75 different breeds. Distinct, breed-specific hematological phenotypes were identified by two complementary analytical methods. Some of these phenotypes corroborated existing literature (e.g. the low MCV of Ancient breeds [19,20]), but others have hitherto not been described (e.g. the relatively high neutrophil concentrations of SBT; the relatively high platelet concentrations of Pekingese).

One of the first considerations in the analysis of this dataset was the impact of age, sex and neutering status, which were all potential confounders in the dissection of breed effects. All measurands other than MCHC varied with age. Consistent with their physiological relationship, RBC concentration, hematocrit and hemoglobin concentration all showed a similar pattern, increasing to 2‒4 years of age and then decreasing thereafter – though the absolute change in each of these parameters was small. All three parameters also showed an interaction between age and neutering status, neutered dogs showing less pronounced variations in these parameters with age. A similar pattern of change was also observed in MCV and MCH. Though there is general recognition that puppies have a lower red cell mass and Hb concentration than adult dogs [25-28], attributed to the maturation of erythropoiesis and a transition from fetal to postnatal erythrocytes [29], this is the first study to document a sustained increase in these parameters to up to four years of age, followed by a gradual decrease to 12 years of age and beyond. We speculate that this observation is a general reflection of continued maturation of erythropoiesis into adulthood, followed by a decrease in erythropoiesis with a decline in renal erythropoietin synthesis and a greater propensity to inflammatory disease with advancing age (‘inflammaging’). The mechanism for the influence of neutering on these parameters remains unclear since it was independent of sex, but we speculate that physiological concentrations of male and female sex hormones may promote erythropoiesis in the dog; in this regard, this study has afforded us a unique opportunity to gauge the impact of sex hormones on hematopoiesis. Age had a variable effect on the values of white cell parameters, which tended to decrease from young to middle age, in common with other canine studies [25,28,30]. In particular, lymphocyte concentrations showed a significant decrease with age, reflecting immunosenescence [31,32], and the early peak in eosinophil concentrations could have reflected – at least in part – higher parasite burdens in younger dogs [17,33,34]. However, in the case of neutrophils and monocytes – and to a lesser extent, lymphocytes and eosinophils – white cell parameters subsequently increased into older age, a novel observation that we attributed to inflammaging. In common with studies of elderly humans [35] a progressive increase in platelet concentration with age was observed, again attributed to increasing inflammatory burden. Interestingly, canine studies to date have suggested that platelet concentrations decrease with age [36], but none has assessed concentrations in such a large cohort of dogs of widely differing age. Nevertheless, the inaccuracy of machine platelet counts and the biases inherent in a cross-sectional study prompt a note of caution in the interpretation of the platelet data, despite the large number of dogs sampled. 

 Sex was also a potential confounding variable. While none of the red cell parameters showed differences between male and female dogs, neutered individuals had higher Hb concentration, MCH and MCHC than intact individuals, though in each case the absolute differences were small and of questionable physiological significance. The implication, however, is that sex hormones may have a subtle impact on these parameters, either by directly influencing erythropoiesis or by an indirect effect on the hematopoietic niche of the bone marrow. Male dogs had higher concentrations of WBC, neutrophils, monocytes and eosinophils, but lower concentrations of platelets, than female dogs. Lower concentrations of platelets have previously been documented in male compared to female beagles [27,37]. Similar patterns have also been observed in species as disparate as rock hyraxes [38] and humans [35], suggesting a differential effect of sex hormones on WBC and platelet dynamics across a spectrum of evolutionarily distant species. We speculate that at physiological concentrations androgens promote the differentiation of the myeloid lineage and estrogens promote the differentiation of the megakaryocytic lineage, but we cannot rule out more trivial explanations such as stress-related distributional effects, to which one sex may be more prone than the other. Nevertheless, tonic levels of both male and female sex hormones are presumably required for optimal hematopoiesis and, or peripheral distribution of myeloid cells and platelets in general, since neutered dogs had lower concentrations of WBC, neutrophils, monocytes and platelets than intact dogs of the same sex. While the preceding observations were made on the basis of the complete dataset of all breeds, similar conclusions could be drawn when the three most populous breeds were specifically examined, suggesting that the influences of age, sex and neutering status on hematological parameters generally transcended breed-specific phenotypes.

 Two complementary analytical techniques were employed to identify distinctive hematological phenotypes, namely PCA of all of the data from pure breed dogs and pairwise comparisons of data from the pure breed and mixed breed dogs. Principal component analysis reduces the dimensionality of a dataset and is often a first step in the classification of samples. Several breeds clustered in a region represented by principal components between -2 and +2, allowing us to identify ‘outlying’ breeds that were thought to have distinctive hematological phenotypes. The first five principal components were considered in our analyses, since they all had Eigenvalues of greater than 1 and cumulatively accounted for more than 80% of the total variance of the dataset. A comparison of the two analytical methods revealed that PCA disclosed 37 breeds with a distinctive phenotype, in contrast to only 29 by pairwise comparisons. Moreover, almost 50% of the breeds identified by PCA were not revealed by pairwise comparisons, arguing for the greater sensitivity of the former method. However, the principal component thresholds of -2 and +2 were arbitrary and increasing them (for example to -3 and +3) would have increased the stringency of the analysis and disclosed fewer ‘unique’ breeds. Notwithstanding this caveat, several of the breeds identified by PCA were those with published hematological phenotypes – for example the shar pei [39], Rottweiler [17], CKCS [14] and greyhound [15,16]. Several other breeds had not previously been identified in the literature and were also highlighted in our pairwise comparisons. Notable examples included the relatively low MCV of the miniature dachshund, the relatively high platelet concentration of the pug, the relatively high neutrophil concentration of the Border terrier, the relatively high lymphocyte concentration of the Siberian husky and the relatively low eosinophil concentration of the Irish setter. One of the limitations of our study was the inevitable inclusion of samples from unhealthy dogs. Unfortunately, not all blood samples were associated with clear information on why the samples were submitted, precluding a systematic analysis of this possibility. While the impact of disease and medications would have been minimized by our acceptance of profiles only when all parameters (other than platelet concentration) fell within the DL reference intervals, we could not exclude the possibility of small deviations occurring as a consequence of such factors. Certain parameters would be more vulnerable to these effects than others: for example, the low eosinophil concentration of WHWT could be a reflection of a breed-specific hematological phenotype or the propensity of this breed to develop atopy and the widespread use of corticosteroids in the treatment of this condition. Furthermore, the tendency of the generic reference interval used in the selection of cases recruited into this study to truncate the breed-specific data for certain parameters meant that for these values at least, the calculated reference intervals were likely to be inaccurate. We therefore censored any parameters for which more than 2% of total individuals had values at the existing DL reference limit. A direct sampling strategy would have circumvented all such shortcomings, but the accrual of homogeneous data from thousands of healthy dogs in this way would have been impracticable. Nevertheless, a prospective study of this nature is a future aspiration based on the current data. 

 Despite these provisos, this study highlights the intriguing phenotypic diversity of hematological parameters in the dog, which mirrors the diversity of many other traits in this species. Genome-wide association studies have begun to identify loci governing a number of human hematological parameters, including concentrations of RBC, neutrophils, eosinophils, basophils, lymphocytes, monocytes and platelets [6,7,40,41], yielding important insights into not only normal homeostatic control mechanisms of the hematopoietic system but also, in some cases, susceptibility to disease. For example, a locus associated with platelet concentration is also associated with cardiac disease and a locus associated with monocyte concentration has been linked with renal function and cancer [6,7]. Future studies may also lead to the discovery of novel candidate genes that are somatically mutated in premalignant conditions such as polycythemia vera and myelodysplastic syndrome, and in hematological malignancies [6,7]. Nevertheless, hematological parameters are complex traits influenced by a potentially large number of loci, making genetic studies of this nature challenging by virtue of the sheer number of individuals that need to be sampled to attain sufficient power. Furthermore, the links of these loci to disease are still poorly understood. Given the breed segregation of hematological phenotypes in the dog and the genetic homogeneity of individuals within a breed [42,43], we believe that dogs offer the exciting potential to advance this field. Genetic studies in this species have already identified loci associated with size, behavior and longevity [43-45], as well as a number of diseases, including progressive retinal atrophy [46], degenerative myelopathy [47] and systemic lupus erythematosus [48] – often requiring a fraction of the number of individuals needed for the equivalent human studies. Adding credence to this proposal is the increasing recognition of the value of the dog as a model for human disease [49-52], given the similar etiology and pathogenesis of canine disease in many cases, coupled with the shared environment of dogs and humans. This contrasts with the induced nature and controlled environment of many rodent models. 

 In summary, we have demonstrated that hematological traits in the domestic dog show breed-specific variation when the confounding influences of age, sex and neutering status are accounted for, offering the exciting potential to unravel the genetic constraints on hematological parameters in this species. Such studies are likely to have far-reaching, cross-species ramifications for our understanding of hematopoiesis in both health and disease, and will advance veterinary clinical practice towards breed-specific reference intervals for a number of important hematological parameters. 

## Supporting Information

Figure S1 **Effects of age, sex and neutering status on hematological parameters for the Labrador retriever (n=761).** (a) Age: The adjusted mean values – accounting for sex and neutering status for each of the 12 hematological parameters are represented on the respective *y* axes, showing age in years on the x axes. All measurands except MCV and MCHC varied with age. (b) Sex and neutering status: The adjusted mean values – accounting for age – for each of the 12 hematological parameters are represented on the respective *y* axes, showing sex (F=female; M=male) and neutering status (I=intact; N=neutered) on the x axes. When present, significant differences (in sex, neutering status, or sex*neutering status interaction) are shown at the top of the figure; in some cases, only the interaction between sex and neutering status was significant.
(TIF)

Figure S2 **Effects of age, sex and neutering status on hematological parameters for the German shepherd dog (n=346).** (a) Age: The adjusted mean values – accounting for sex and neutering status – for each of the 12 hematological parameters are represented on the respective *y* axes, showing age in years on the *x* axes. When present, significant differences in age are shown at the top of the figure. (b) Sex and neutering status: The adjusted mean values – accounting for age – for each of the 12 hematological parameters are represented on the respective *y* axes, showing sex (F=female; M=male) and neutering status (I=intact; N=neutered) on the *x* axes. When present, significant differences (in sex, neutering status, or sex*neutering status interaction) are shown at the top of the figure.
(TIF)

Figure S3 **Effects of age, sex and neutering status on hematological parameters for the boxer (n=351).** (a) Age: The adjusted mean values – accounting for sex and neutering status – for each of the 12 hematological parameters are represented on the respective *y* axes, showing age in years on the *x* axes. When present, significant differences in age are shown at the top of the figure. (b) Sex and neutering status: The adjusted mean values – accounting for age – for each of the 12 hematological parameters are represented on the respective *y* axes, showing sex (F=female; M=male) and neutering status (I=intact; N=neutered) on the *x* axes. When present, significant differences (in sex, neutering status, or sex*neutering status interaction) are shown at the top of the figure.
(TIF)

Figure S4 **A comparison of the results of principal component analysis with pairwise comparative analysis.** Breeds with a distinctive phenotype identified by both methods are shown in bold text and joined by a line, *versus* those identified only by one or the other analytical method. Complementarity was generally observed between the methods.
(TIF)

Figure S5 **Histograms of the hematological data for the Labrador retriever (n=761).**
(TIF)

Figure S6 **Histograms of the hematological data for the German shepherd dog (n=346).**
(TIF)

Figure S7 **Histograms of the hematological data for the Cavalier King Charles spaniel (n=280).**
(TIF)

Figure S8 **Histograms of the hematological data for the cocker spaniel (n=227).**
(TIF)

Figure S9 **Histograms of the hematological data for the West Highland white terrier (n=199).**
(TIF)

Figure S10 **Histograms of the hematological data for the Jack Russell terrier (n=180).**
(TIF)

Figure S11 **Histograms of the hematological data for the golden retriever (n=171).**
(TIF)

Figure S12 **Histograms of the hematological data for the springer spaniel (n=168).**
(TIF)

Figure S13 **Histograms of the hematological data for the Staffordshire bull terrier (n=165).**
(TIF)

Figure S14 **Histograms of the hematological data for the Yorkshire terrier (n=154).**
(TIF)

Figure S15 **Histograms of the hematological data for the Rottweiler (n=128).**
(TIF)

Table S1 **Descriptive statistics – red blood cell concentration^§^.** § Unit of measurement: x 10^12^/L; SD = standard deviation; IQR = interquartile range; Min. = minimum value recorded; Max. = maximum value recorded.
(DOC)

Table S2 **Descriptive statistics – mean corpuscular volume^§^.** § Unit of measurement: fL; SD = standard deviation; IQR = interquartile range; Min. = minimum value recorded; Max. = maximum value recorded.
(DOC)

Table S3 **Descriptive statistics – hematocrit^§^.** § Stated as a percentage; SD = standard deviation; IQR = interquartile range; Min. = minimum value recorded; Max. = maximum value recorded.
(DOC)

Table S4 **Descriptive statistics – hemoglobin^§^.** § Unit of measurement: g/dL; SD = standard deviation; IQR = interquartile range; Min. = minimum value recorded; Max. = maximum value recorded.
(DOC)

Table S5 **Descriptive statistics – mean corpuscular hemoglobin^§^.** § Unit of measurement: pg; SD = standard deviation; IQR = interquartile range; Min. = minimum value recorded; Max. = maximum value recorded.
(DOC)

Table S6 **Descriptive statistics – mean cell hemoglobin concentration^§^.** § Unit of measurement: g/dL; SD = standard deviation; IQR = interquartile range; Min. = minimum value recorded; Max. = maximum value recorded.
(DOC)

Table S7 **Descriptive statistics – white blood cell concentration^§^.** § Unit of measurement: x 10^9^/L; SD = standard deviation; IQR = interquartile range; Min. = minimum value recorded; Max. = maximum value recorded.
(DOC)

Table S8 **Descriptive statistics – neutrophil concentration^§^.** § Unit of measurement: x 10^9^/L; SD = standard deviation; IQR = interquartile range; Min. = minimum value recorded; Max. = maximum value recorded.
(DOC)

Table S9 **Descriptive statistics – monocyte concentration^§^.** § Unit of measurement: x 10^9^/L; SD = standard deviation; IQR = interquartile range; Min. = minimum value recorded; Max. = maximum value recorded.
(DOC)

Table S10 **Descriptive statistics – lymphocyte concentration^§^.** § Unit of measurement: x 10^9^/L; SD = standard deviation; IQR = interquartile range; Min. = minimum value recorded; Max. = maximum value recorded.
(DOC)

Table S11 **Descriptive statistics – eosinophil concentration^§^.** § Unit of measurement: x 10^9^/L; SD = standard deviation; IQR = interquartile range; Min. = minimum value recorded; Max. = maximum value recorded.
(DOC)

Table S12 **Descriptive statistics – platelet concentration^§^.** § Unit of measurement: x 10^9^/L; SD = standard deviation; IQR = interquartile range; Min. = minimum value recorded; Max. = maximum value recorded.
(DOC)

Table S13 **Statistical analysis of the effects of age, sex and neutering status.** This table shows the results of a linear mixed effects model to assess the effect of age, sex and neutering status, and all two-way and three-way interactions on each hematological parameter, taking breed as a random effect.
Red cell parameters
Abbreviations: RBC=red blood cell concentration; MCV=mean corpuscular volume; Hct=hematocrit; Hb=hemoglobin; MCH=mean corpuscular hemoglobin; MCHC=mean corpuscular hemoglobin concentration.
White cell and platelet concentrations
Abbreviations: Concentrations of WBC=white blood cells, Neut=neutrophils, Mono=monocytes, Lymph=lymphocytes, Eosin=eosinophils and PLT=platelets
(DOC)

Table S14 **Principal component analysis – Eigenvalues of the correlation matrix.**
(DOC)

Table S15 **Statistical analysis of pairwise comparisons of hematological parameters between the pure breed and mixed breed dogs.** The results of two-sample Kolmogorov-Smirnov tests to compare the distributions of residuals for the pure breed *versus* mixed breed dogs are shown as the -log_10_(p value). (Residuals were defined as the observed values minus the estimated fixed effects of age, sex and neutering status.) A Bonferroni correction to account for multiple comparisons was applied, yielding a threshold for significance of 10^-4^ (i.e. 4 as stated in the table).
Abbreviations: RBC=red blood cell concentration; MCV=mean corpuscular volume; Hct=hematocrit; Hb=hemoglobin; MCH=mean corpuscular hemoglobin; MCHC=mean corpuscular hemoglobin concentration; concentrations of WBC=white blood cell, Neut=neutrophils, Mono=monocytes, Lymph=lymphocytes, Eosin=eosinophils and PLT=platelets.
(DOC)

Table S16 **Tentative breed-specific reference intervals for the Labrador retriever (n=761).** Abbreviations: RBC, red blood cells; Hb, hemoglobin concentration; Hct, hematocrit; MCV, mean corpuscular volume; MCH, mean corpuscular hemoglobin; WBC, white blood cells; RI, reference interval; F, female; M, male; I, intact; N, neutered; *, undetermined owing to data truncation; §, these values fell below (above) the current lower (upper) RIs because they were calculated lower (upper) limits, i.e. the estimated 2.5% (97.5%) of the residuals plus the adjusted means accounting for age, sex and neutering status for each measurand.
(DOC)

Table S17 **Tentative breed-specific reference intervals for the German shepherd dog (n=346).** Abbreviations: RBC, red blood cells; Hb, hemoglobin concentration; Hct, hematocrit; MCV, mean corpuscular volume; MCH, mean corpuscular hemoglobin; WBC, white blood cells; RI, reference interval; F, female; M, male; I, intact; N, neutered; *, undetermined owing to data truncation; §, these values fell below (above) the current lower (upper) RIs because they were calculated lower (upper) limits, i.e. the estimated 2.5% (97.5%) of the residuals plus the adjusted means accounting for age, sex and neutering status for each measurand.
(DOC)

Table S18 **Tentative breed-specific reference intervals for the Cavalier King Charles spaniel (n=280).** Abbreviations: RBC, red blood cells; Hb, hemoglobin concentration; Hct, hematocrit; MCV, mean corpuscular volume; MCH, mean corpuscular hemoglobin; WBC, white blood cells; RI, reference interval; F, female; M, male; I, intact; N, neutered; *, undetermined owing to data truncation; §, these values fell below (above) the current lower (upper) RIs because they were calculated lower (upper) limits, i.e. the estimated 2.5% (97.5%) of the residuals plus the adjusted means accounting for age, sex and neutering status for each measurand.
(DOC)

Table S19 **Tentative breed-specific reference intervals for the cocker spaniel (n=227).** Abbreviations: RBC, red blood cells; Hb, hemoglobin concentration; Hct, hematocrit; MCV, mean corpuscular volume; MCH, mean corpuscular hemoglobin; WBC, white blood cells; RI, reference interval; F, female; M, male; I, intact; N, neutered; *, undetermined owing to data truncation; §, these values fell below (above) the current lower (upper) RIs because they were calculated lower (upper) limits, i.e. the estimated 2.5% (97.5%) of the residuals plus the adjusted means accounting for age, sex and neutering status for each measurand.
(DOC)

Table S20 **Tentative breed-specific reference intervals for the West Highland white terrier (n=199).** Abbreviations: RBC, red blood cells; Hb, hemoglobin concentration; Hct, hematocrit; MCV, mean corpuscular volume; MCH, mean corpuscular hemoglobin; WBC, white blood cells; RI, reference interval; F, female; M, male; I, intact; N, neutered; *, undetermined owing to data truncation; §, these values fell below (above) the current lower (upper) RIs because they were calculated lower (upper) limits, i.e. the estimated 2.5% (97.5%) of the residuals plus the adjusted means accounting for age, sex and neutering status for each measurand.
(DOC)

Table S21 **Tentative breed-specific reference intervals for the Jack Russell terrier (n=180).** Abbreviations: RBC, red blood cells; Hb, hemoglobin concentration; Hct, hematocrit; MCV, mean corpuscular volume; MCH, mean corpuscular hemoglobin; WBC, white blood cells; RI, reference interval; F, female; M, male; I, intact; N, neutered; *, undetermined owing to data truncation; §, these values fell below (above) the current lower (upper) RIs because they were calculated lower (upper) limits, i.e. the estimated 2.5% (97.5%) of the residuals plus the adjusted means accounting for age, sex and neutering status for each measurand.
(DOC)

Table S22 **Tentative breed-specific reference intervals for the golden retriever (n=171).** Abbreviations: RBC, red blood cells; Hb, hemoglobin concentration; Hct, hematocrit; MCV, mean corpuscular volume; MCH, mean corpuscular hemoglobin; WBC, white blood cells; RI, reference interval; F, female; M, male; I, intact; N, neutered; *, undetermined owing to data truncation; §, these values fell below (above) the current lower (upper) RIs because they were calculated lower (upper) limits, i.e. the estimated 2.5% (97.5%) of the residuals plus the adjusted means accounting for age, sex and neutering status for each measurand.
(DOC)

Table S23 **Tentative breed-specific reference intervals for the springer spaniel (n=168).** Abbreviations: RBC, red blood cells; Hb, hemoglobin concentration; Hct, hematocrit; MCV, mean corpuscular volume; MCH, mean corpuscular hemoglobin; WBC, white blood cells; RI, reference interval; F, female; M, male; I, intact; N, neutered; *, undetermined owing to data truncation; §, these values fell below (above) the current lower (upper) RIs because they were calculated lower (upper) limits, i.e. the estimated 2.5% (97.5%) of the residuals plus the adjusted means accounting for age, sex and neutering status for each measurand.
(DOC)

Table S24 **Tentative breed-specific reference intervals for the Staffordshire bull terrier (n=165).** Abbreviations: RBC, red blood cells; Hb, hemoglobin concentration; Hct, hematocrit; MCV, mean corpuscular volume; MCH, mean corpuscular hemoglobin; WBC, white blood cells; RI, reference interval; F, female; M, male; I, intact; N, neutered; *, undetermined owing to data truncation; §, these values fell below (above) the current lower (upper) RIs because they were calculated lower (upper) limits, i.e. the estimated 2.5% (97.5%) of the residuals plus the adjusted means accounting for age, sex and neutering status for each measurand.
(DOC)

Table S25 **Tentative breed-specific reference intervals for the Yorkshire terrier (n=154).** Abbreviations: RBC, red blood cells; Hb, hemoglobin concentration; Hct, hematocrit; MCV, mean corpuscular volume; MCH, mean corpuscular hemoglobin; WBC, white blood cells; RI, reference interval; F, female; M, male; I, intact; N, neutered; *, undetermined owing to data truncation; §, these values fell below (above) the current lower (upper) RIs because they were calculated lower (upper) limits, i.e. the estimated 2.5% (97.5%) of the residuals plus the adjusted means accounting for age, sex and neutering status for each measurand.
(DOC)

Table S26 **Tentative breed-specific reference intervals for the Rottweiler (n=128).** Abbreviations: RBC, red blood cells; Hb, hemoglobin concentration; Hct, hematocrit; MCV, mean corpuscular volume; MCH, mean corpuscular hemoglobin; WBC, white blood cells; RI, reference interval; F, female; M, male; I, intact; N, neutered; *, undetermined owing to data truncation; §, these values fell below (above) the current lower (upper) RIs because they were calculated lower (upper) limits, i.e. the estimated 2.5% (97.5%) of the residuals plus the adjusted means accounting for age, sex and neutering status for each measurand.
(DOC)

Materials and Methods S1 (DOC)
