# Supplementary figures and images for: Breed-Specific Hematological Phenotypes in the Dog: A Natural Resource for the Genetic Dissection of Hematological Parameters in a Mammalian Species
Source: PLoS One. 2013 Nov 25;8(11):e81288. doi: 10.1371/journal.pone.0081288 (PMC3840015; doi:10.1371/journal.pone.0081288)

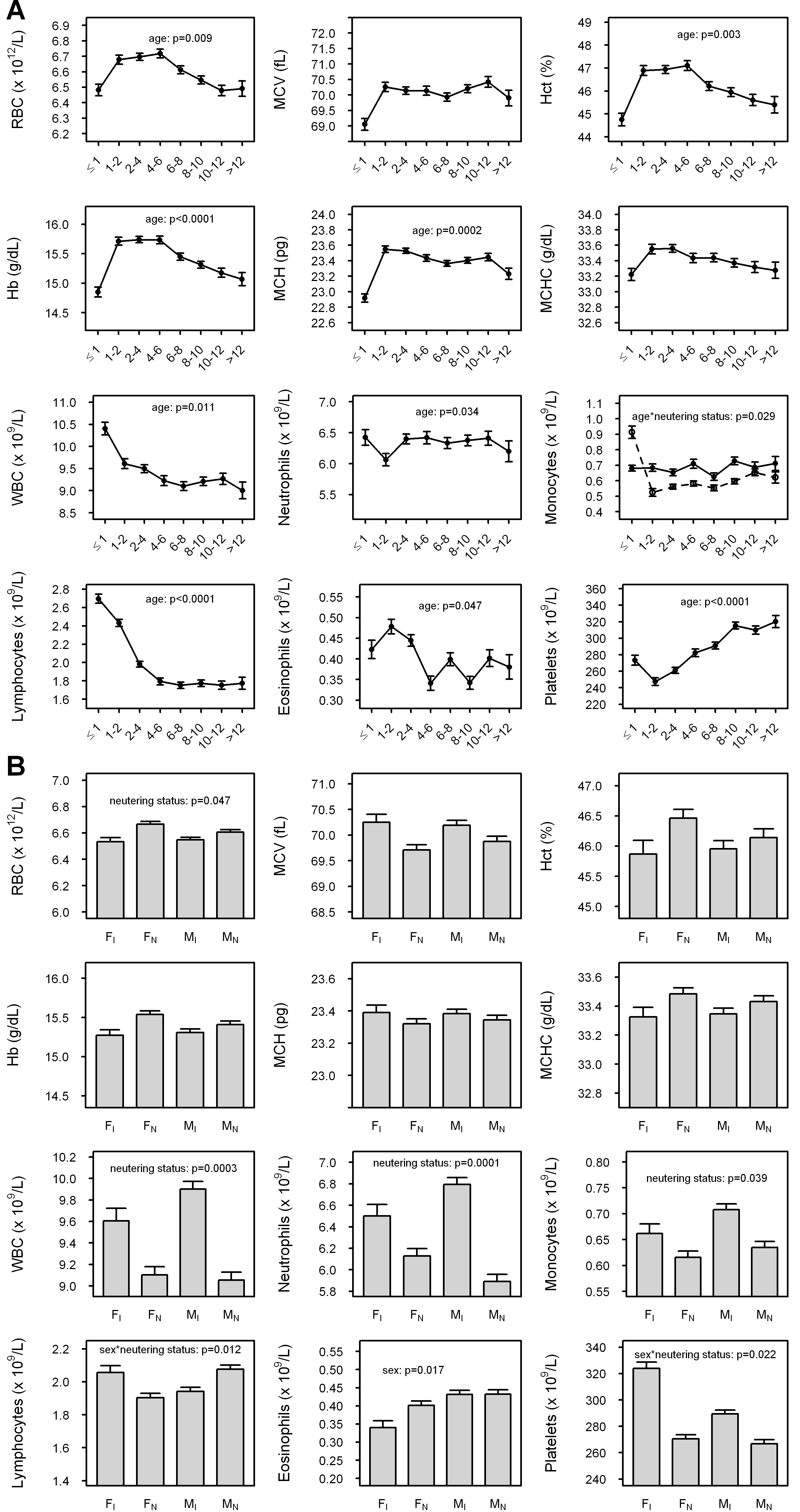

Supplement: Figure S1 — Effects of age, sex and neutering status on hematological parameters for the Labrador retriever (n=761). (a) Age: The adjusted mean values – accounting for sex and neutering status for each of the 12 hematological parameters are represented on the respective y axes, showing age in years on the x axes. All measurands except MCV and MCHC varied with age. (b) Sex and neutering status: The adjusted mean values – accounting for age – for each of the 12 hematological parameters are represented on the respective y axes, showing sex (F=female; M=male) and neutering status (I=intact; N=neutered) on the x axes. When present, significant differences (in sex, neutering status, or sex*neutering status interaction) are shown at the top of the figure; in some cases, only the interaction between sex and neutering status was significant. (TIF) [file pone.0081288.s001.tif]

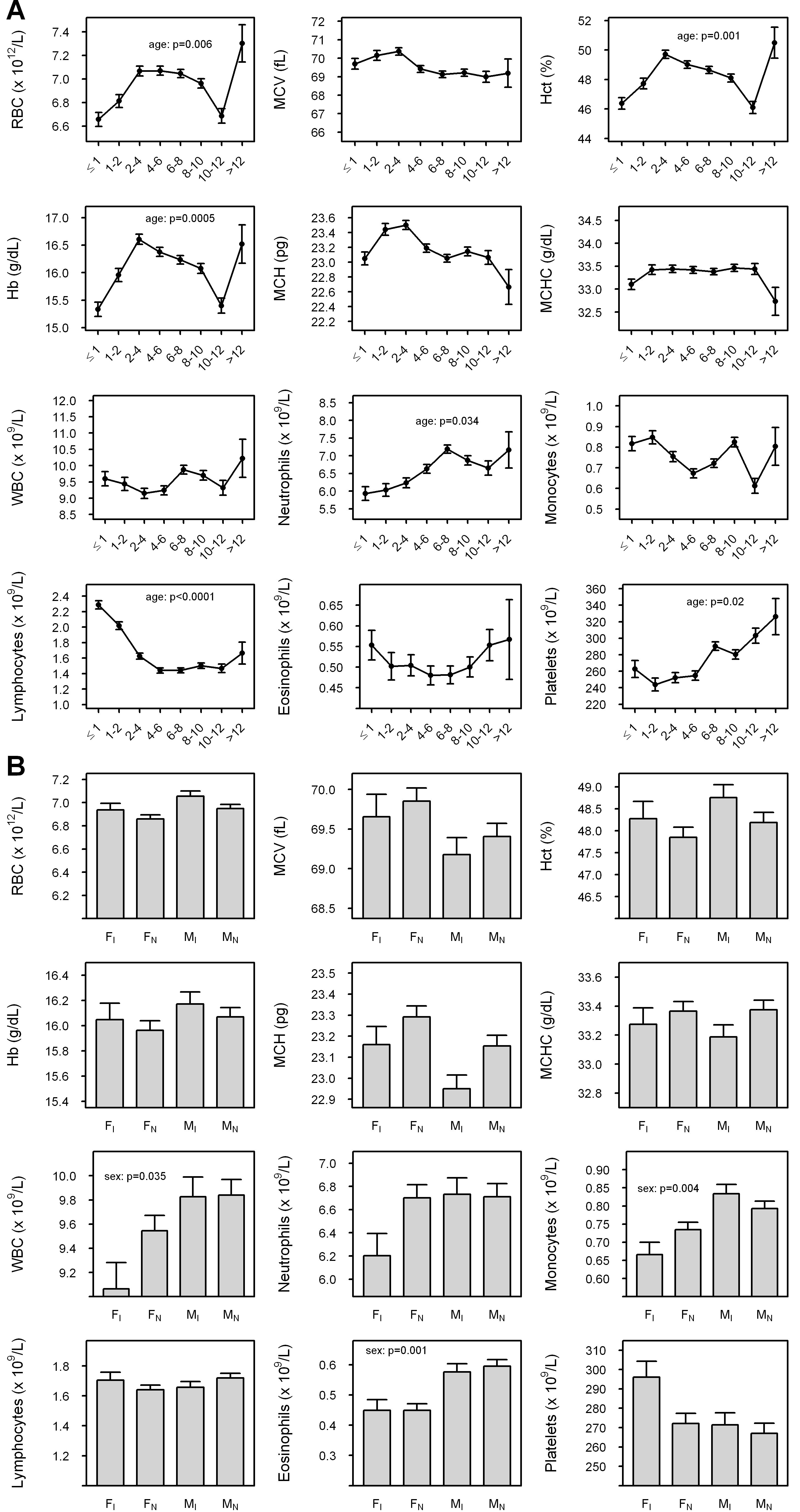

Supplement: Figure S2 — Effects of age, sex and neutering status on hematological parameters for the German shepherd dog (n=346). (a) Age: The adjusted mean values – accounting for sex and neutering status – for each of the 12 hematological parameters are represented on the respective y axes, showing age in years on the x axes. When present, significant differences in age are shown at the top of the figure. (b) Sex and neutering status: The adjusted mean values – accounting for age – for each of the 12 hematological parameters are represented on the respective y axes, showing sex (F=female; M=male) and neutering status (I=intact; N=neutered) on the x axes. When present, significant differences (in sex, neutering status, or sex*neutering status interaction) are shown at the top of the figure. (TIF) [file pone.0081288.s002.tif]

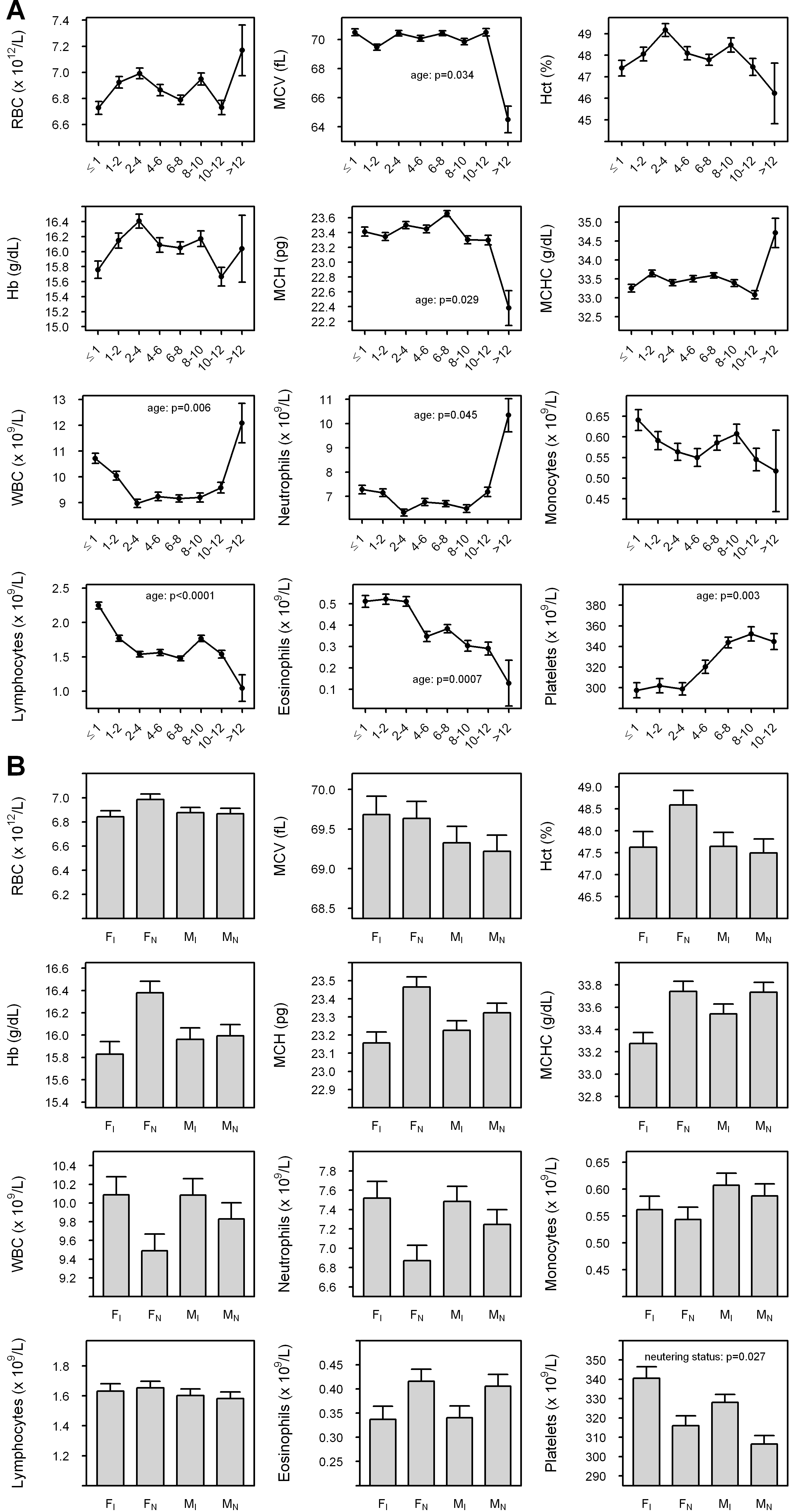

Supplement: Figure S3 — Effects of age, sex and neutering status on hematological parameters for the boxer (n=351). (a) Age: The adjusted mean values – accounting for sex and neutering status – for each of the 12 hematological parameters are represented on the respective y axes, showing age in years on the x axes. When present, significant differences in age are shown at the top of the figure. (b) Sex and neutering status: The adjusted mean values – accounting for age – for each of the 12 hematological parameters are represented on the respective y axes, showing sex (F=female; M=male) and neutering status (I=intact; N=neutered) on the x axes. When present, significant differences (in sex, neutering status, or sex*neutering status interaction) are shown at the top of the figure. (TIF) [file pone.0081288.s003.tif]

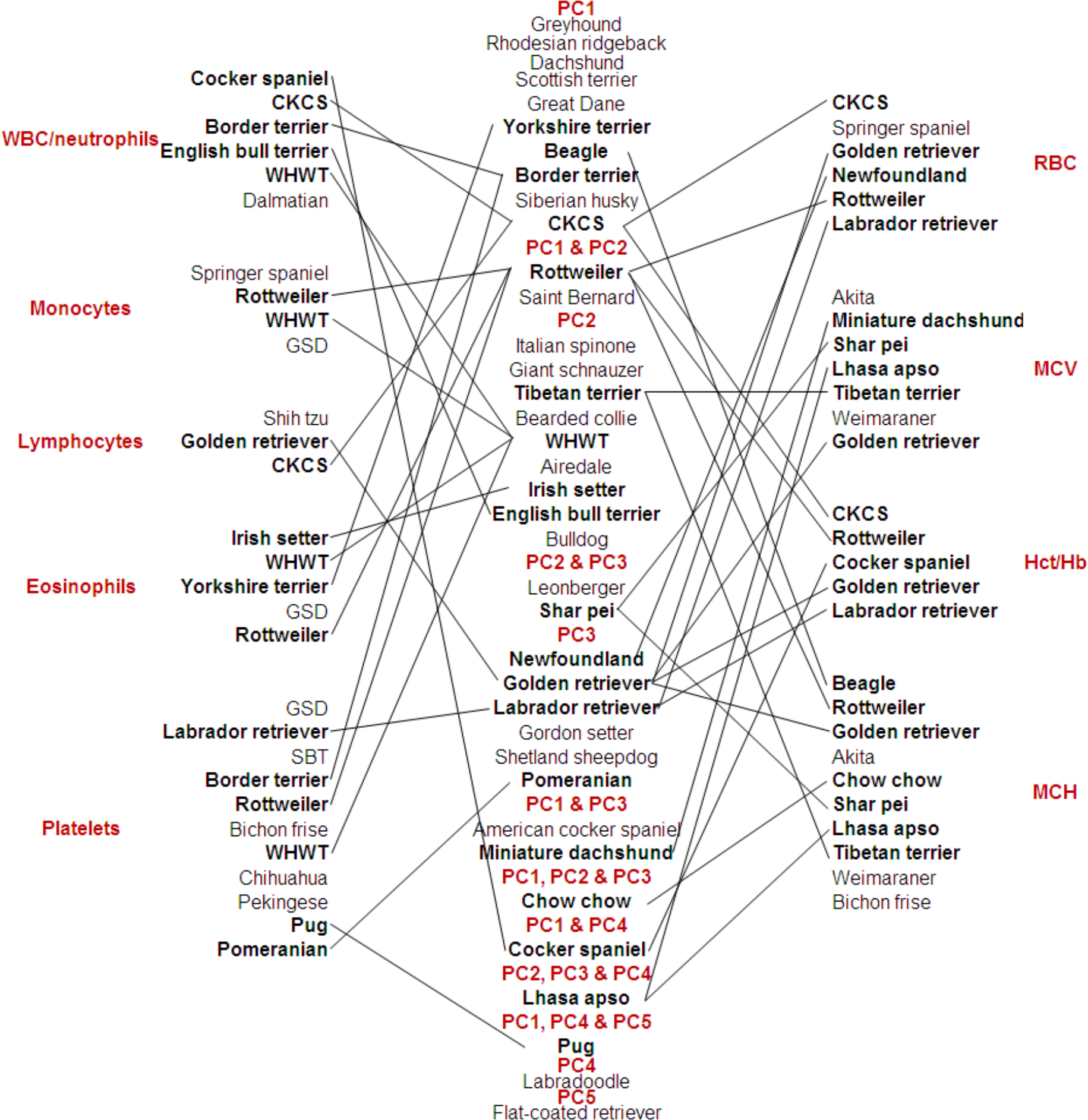

Supplement: Figure S4 — A comparison of the results of principal component analysis with pairwise comparative analysis. Breeds with a distinctive phenotype identified by both methods are shown in bold text and joined by a line, versus those identified only by one or the other analytical method. Complementarity was generally observed between the methods. (TIF) [file pone.0081288.s004.tif]

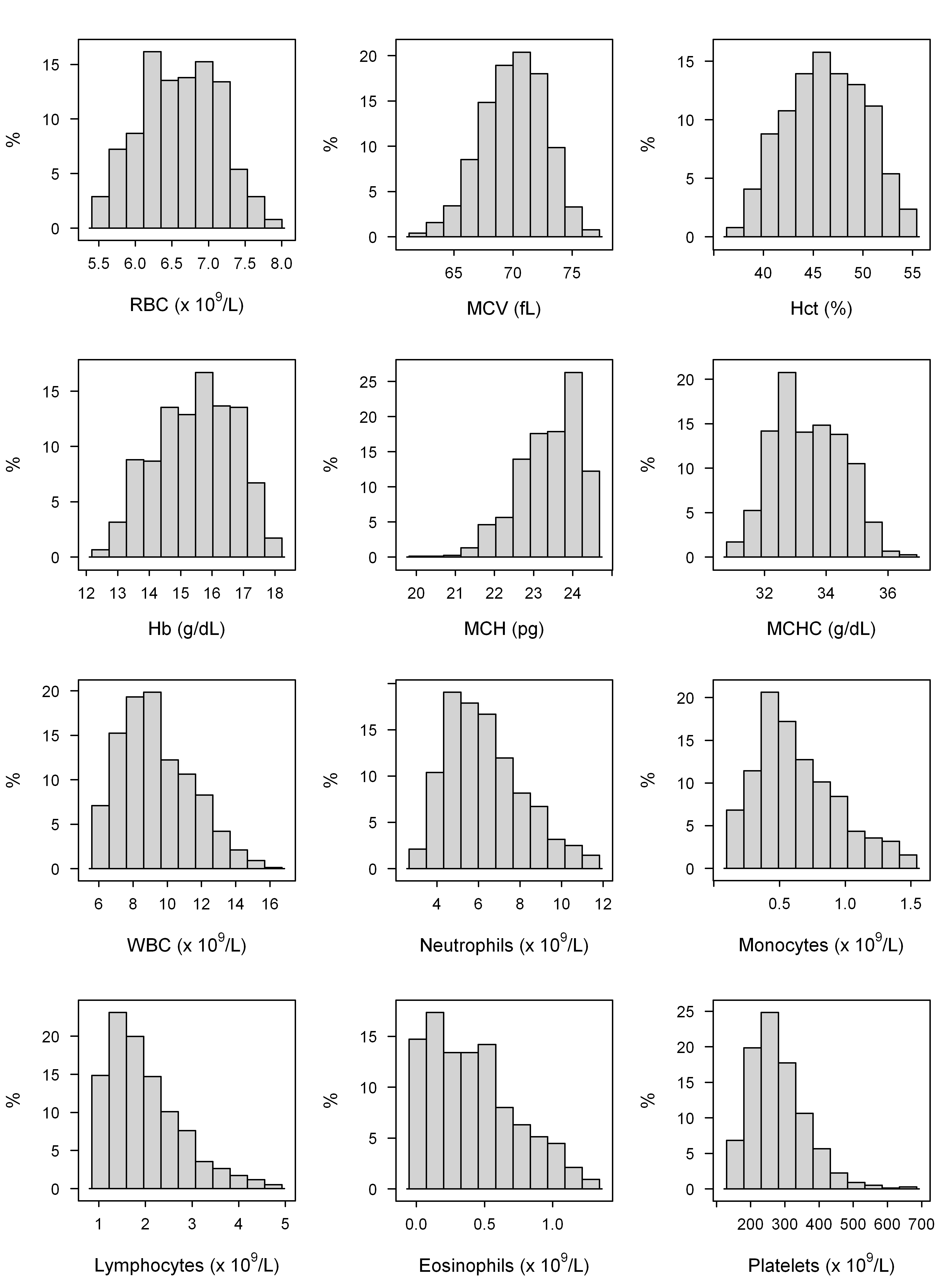

Supplement: Figure S5 — Histograms of the hematological data for the Labrador retriever (n=761). (TIF) [file pone.0081288.s005.tif]

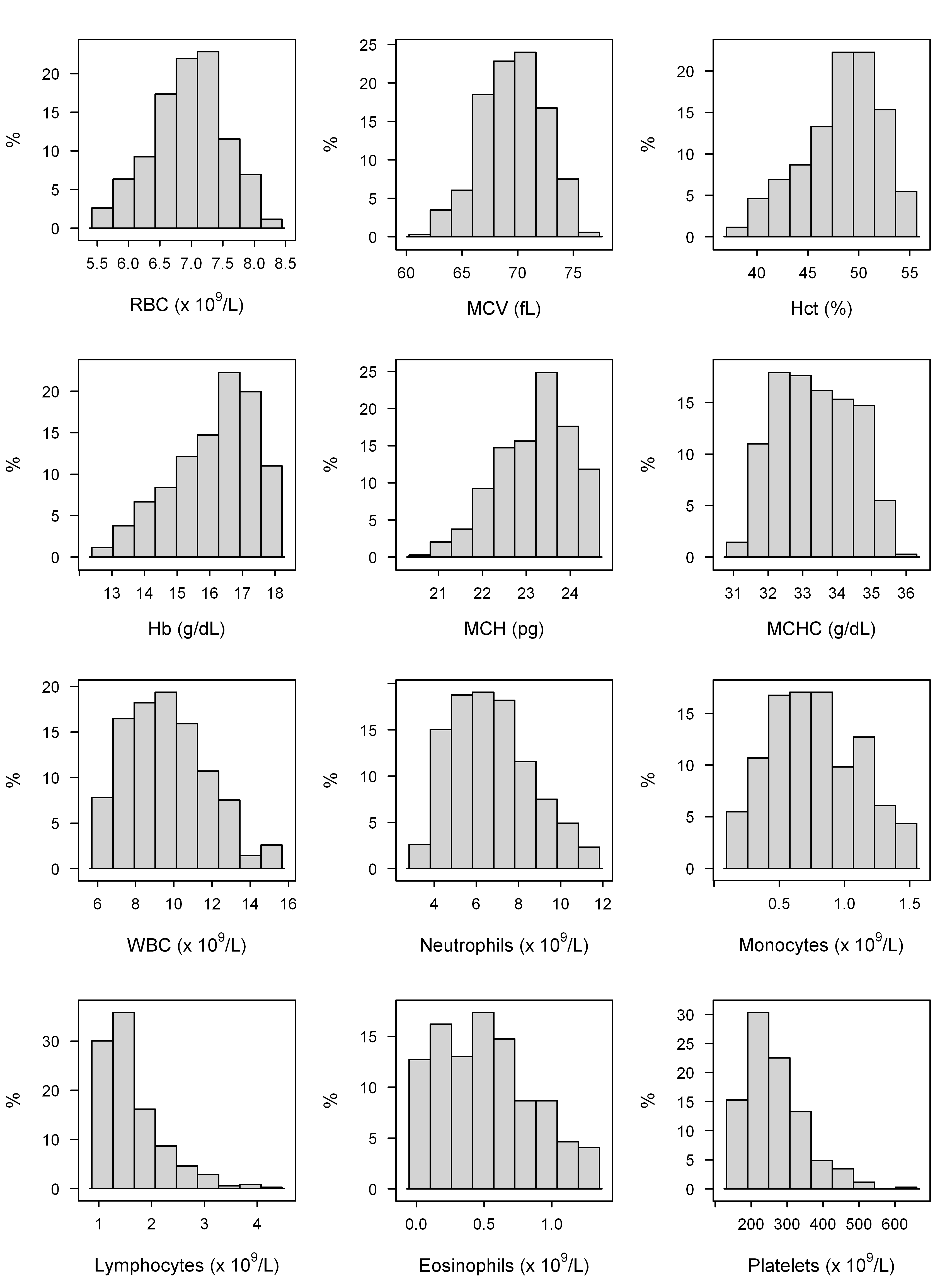

Supplement: Figure S6 — Histograms of the hematological data for the German shepherd dog (n=346). (TIF) [file pone.0081288.s006.tif]

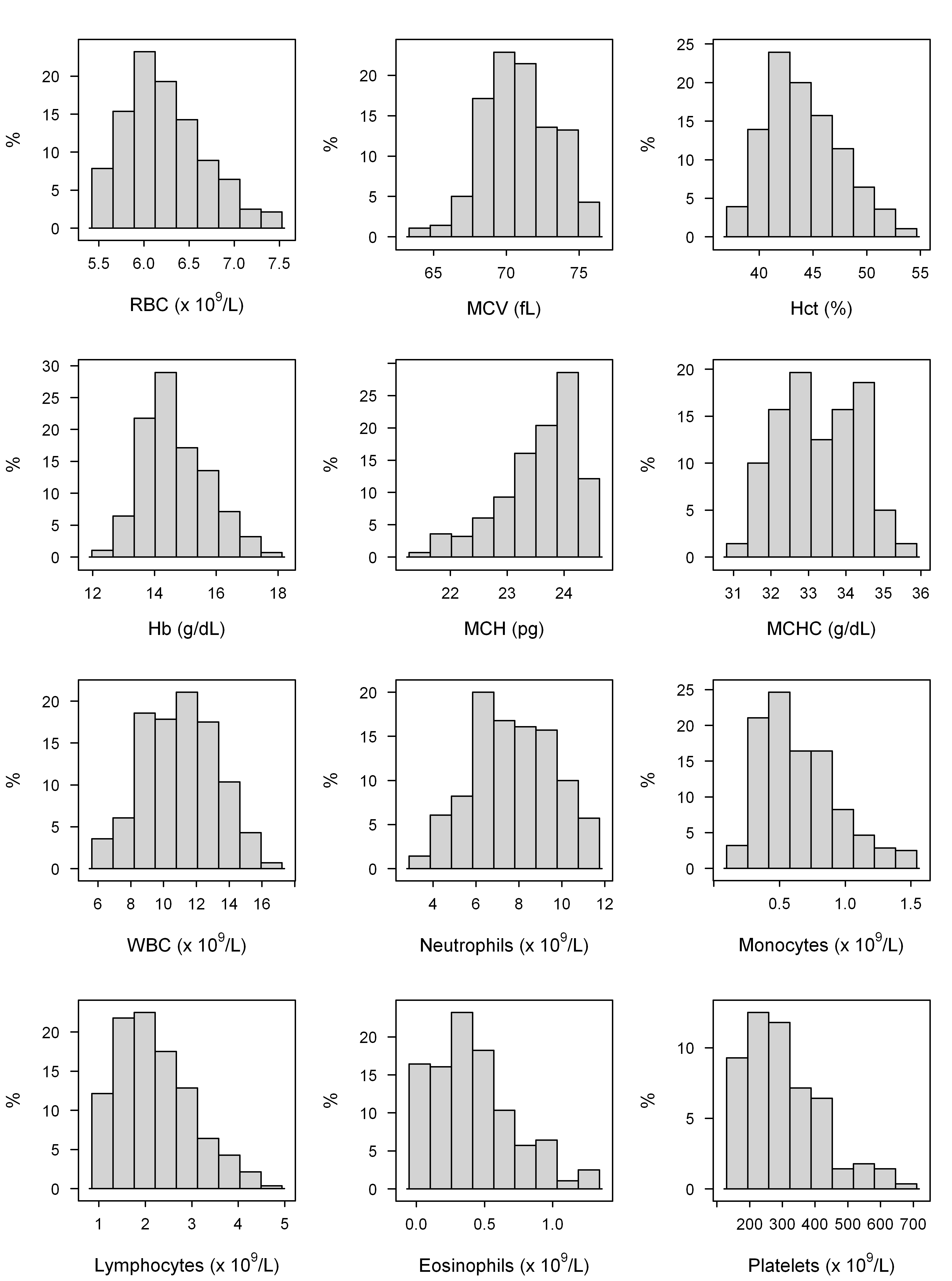

Supplement: Figure S7 — Histograms of the hematological data for the Cavalier King Charles spaniel (n=280). (TIF) [file pone.0081288.s007.tif]

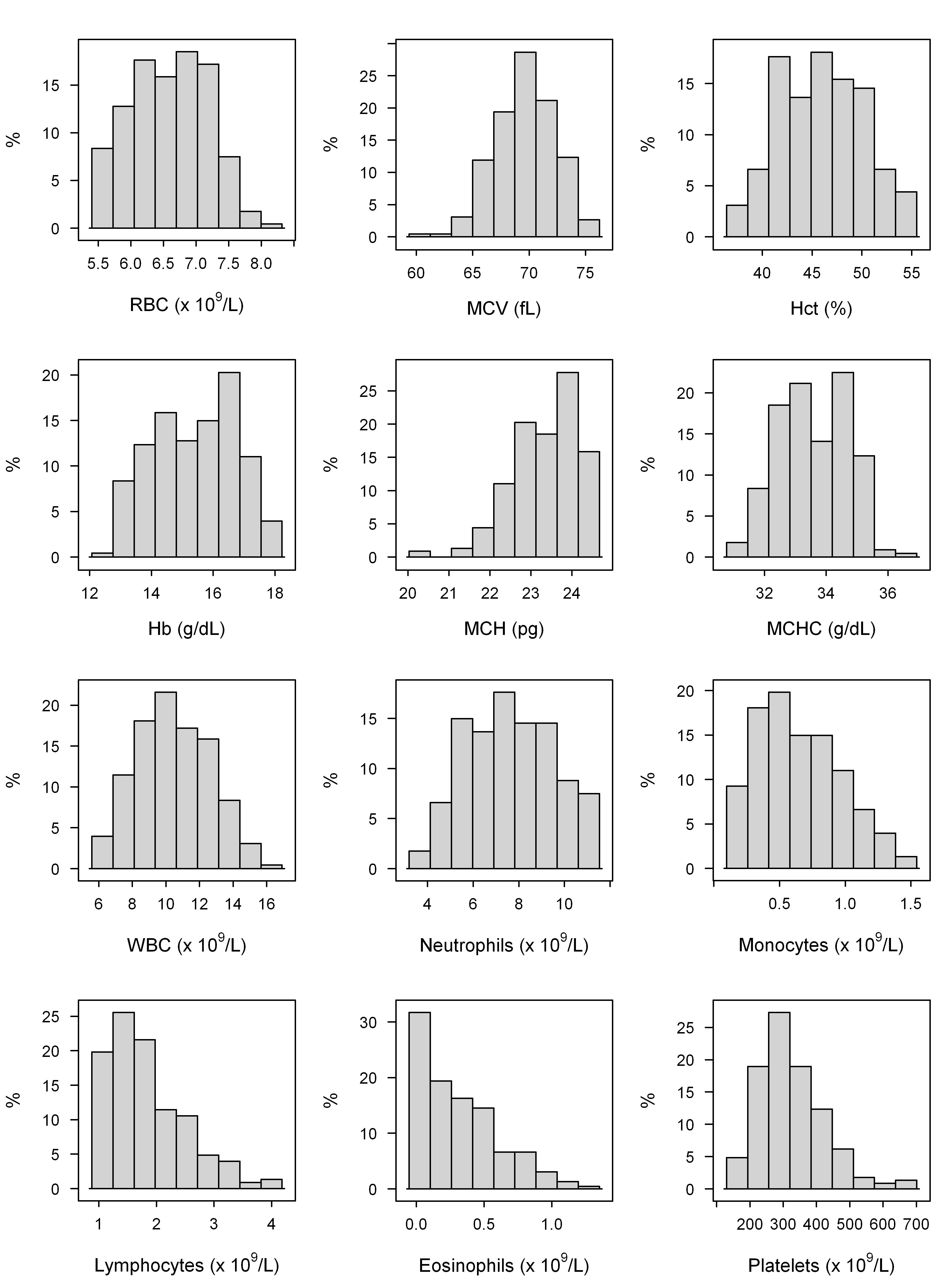

Supplement: Figure S8 — Histograms of the hematological data for the cocker spaniel (n=227). (TIF) [file pone.0081288.s008.tif]

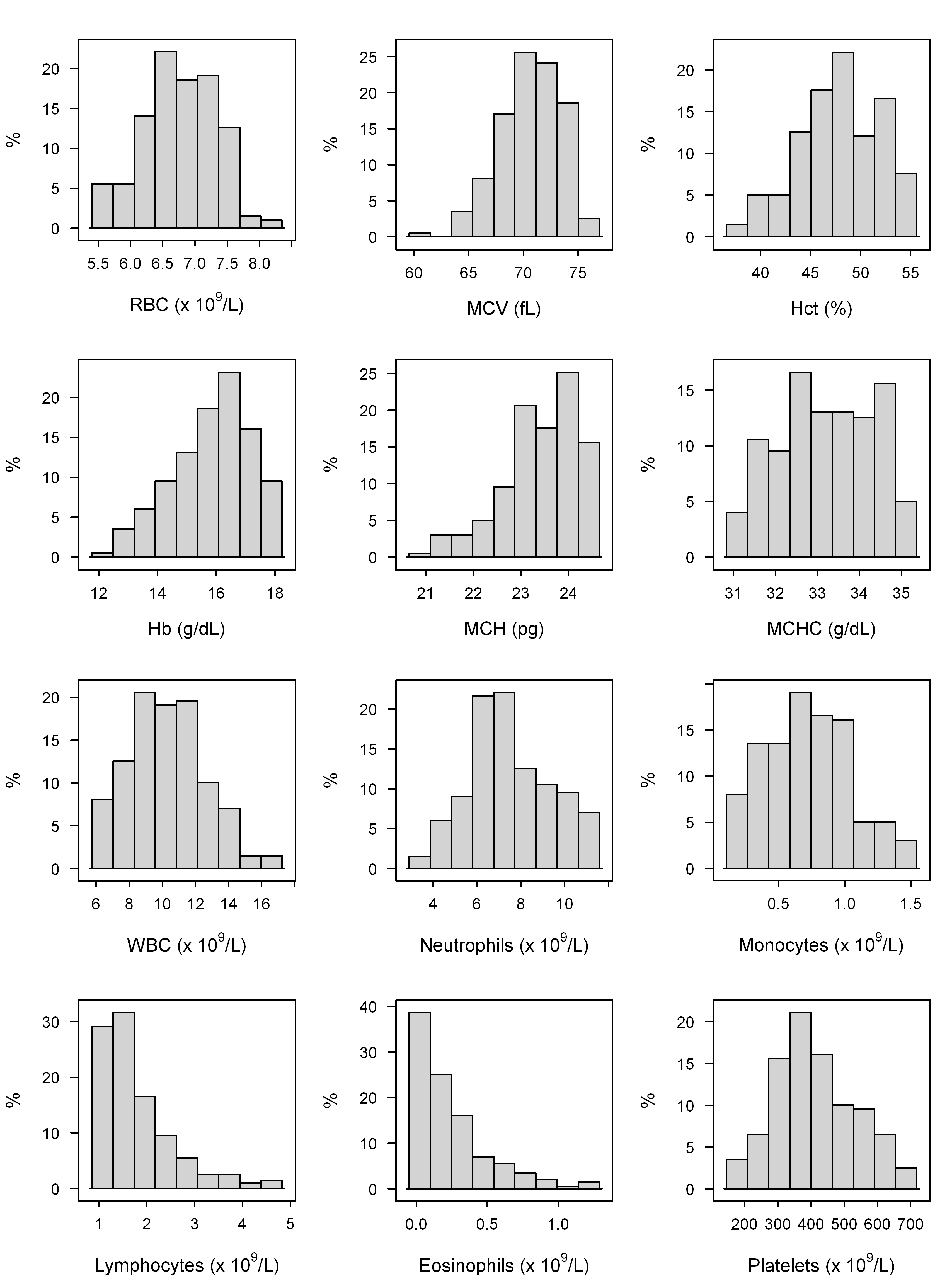

Supplement: Figure S9 — Histograms of the hematological data for the West Highland white terrier (n=199). (TIF) [file pone.0081288.s009.tif]

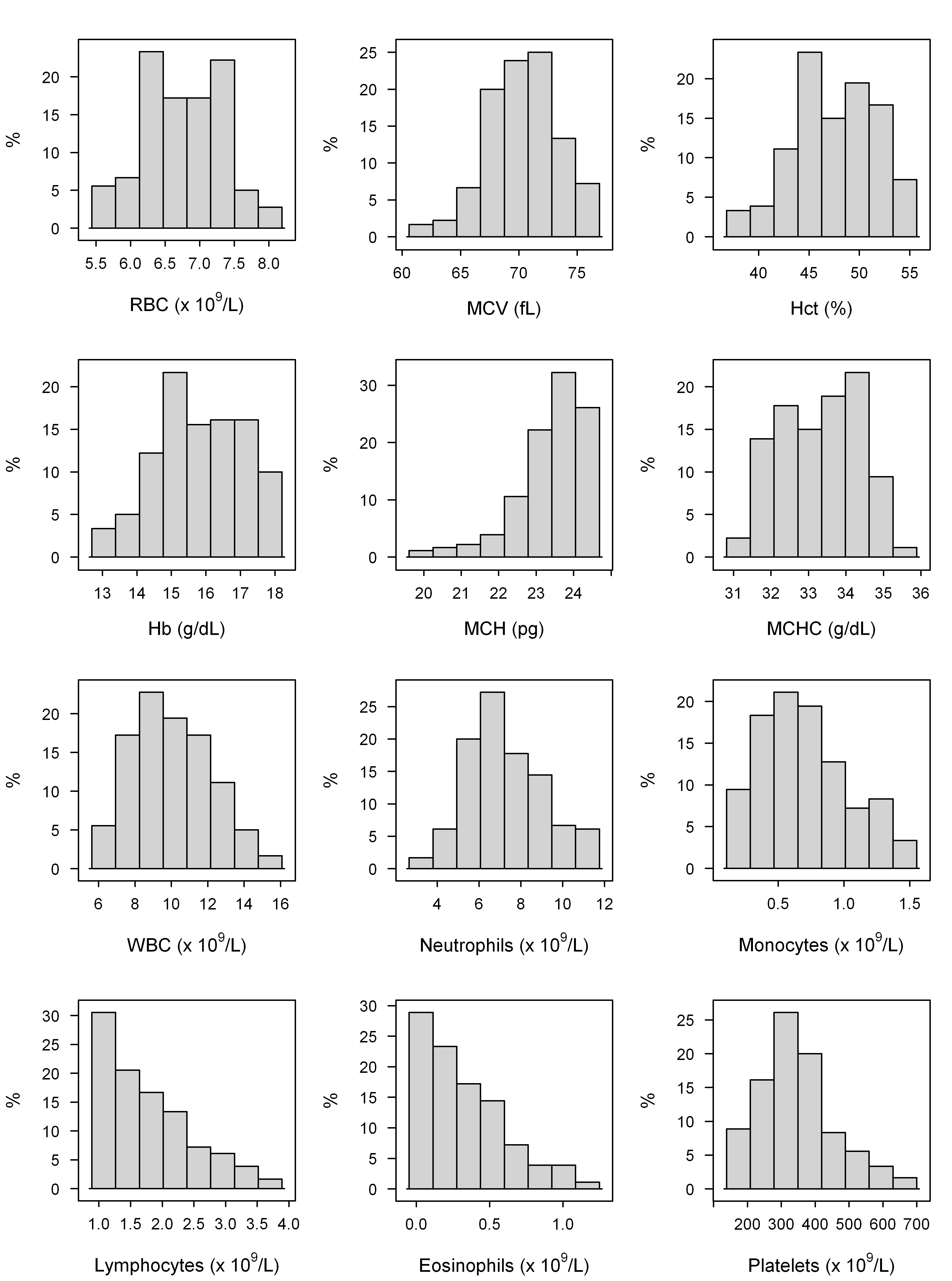

Supplement: Figure S10 — Histograms of the hematological data for the Jack Russell terrier (n=180). (TIF) [file pone.0081288.s010.tif]

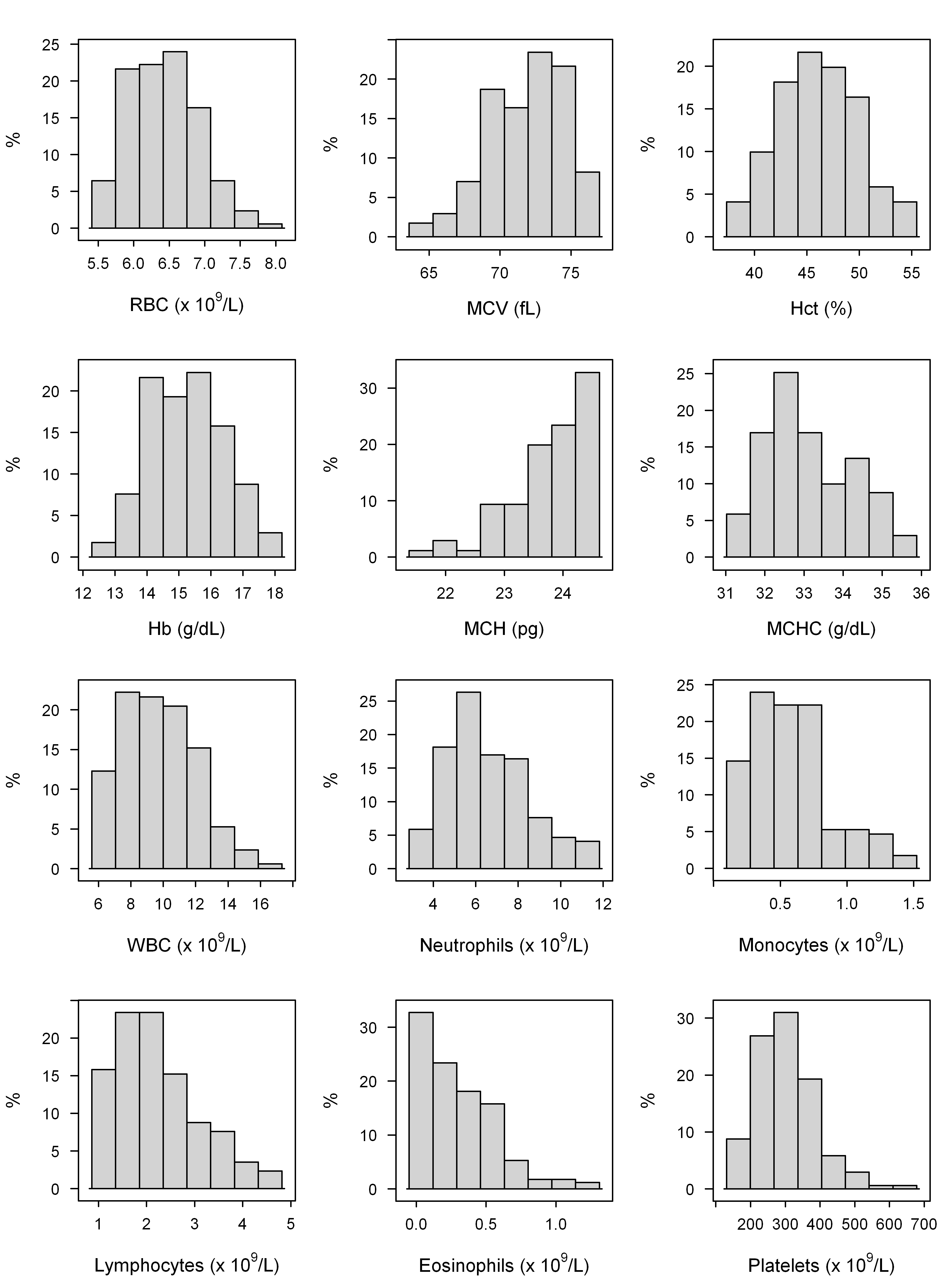

Supplement: Figure S11 — Histograms of the hematological data for the golden retriever (n=171). (TIF) [file pone.0081288.s011.tif]

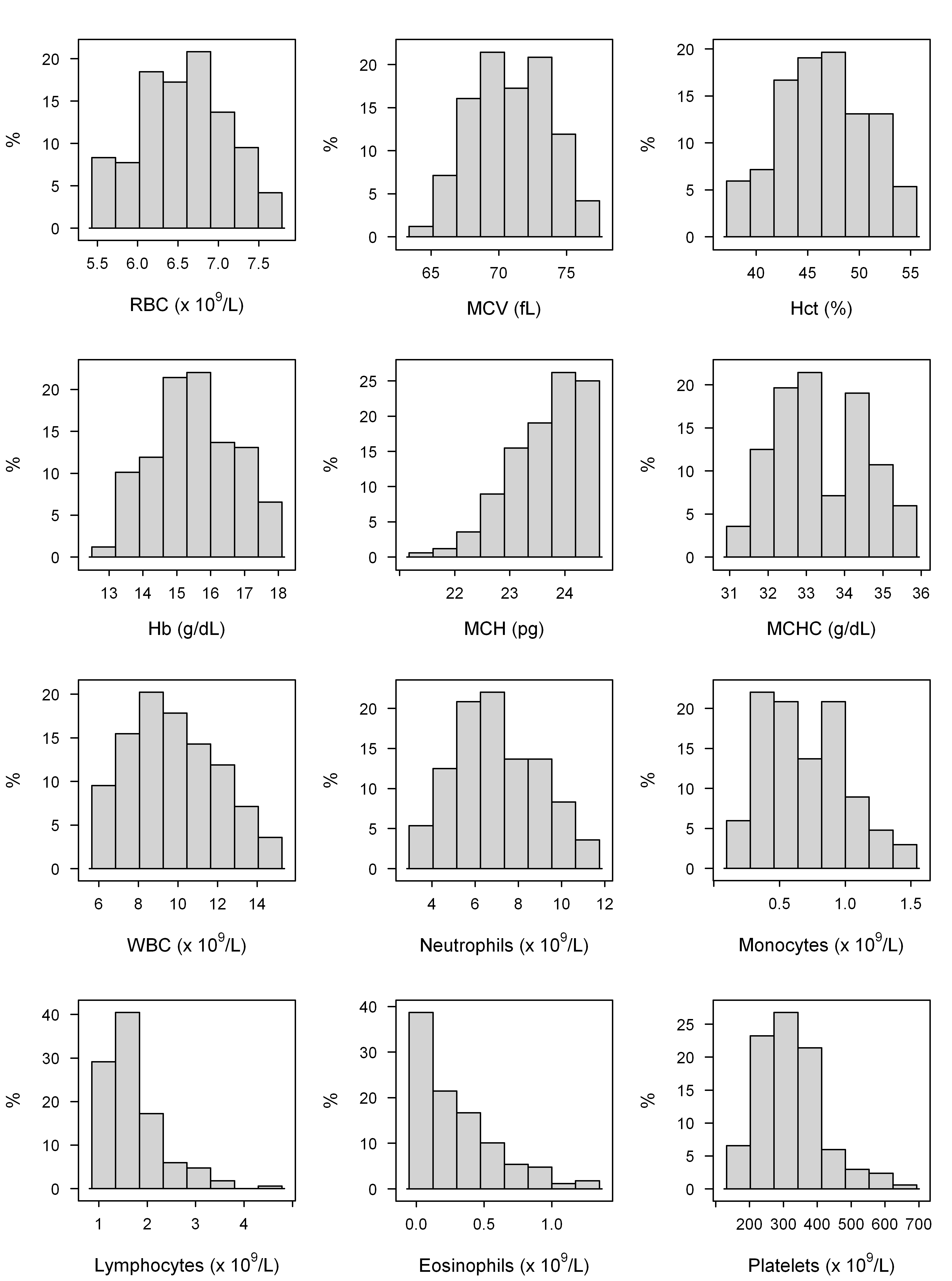

Supplement: Figure S12 — Histograms of the hematological data for the springer spaniel (n=168). (TIF) [file pone.0081288.s012.tif]

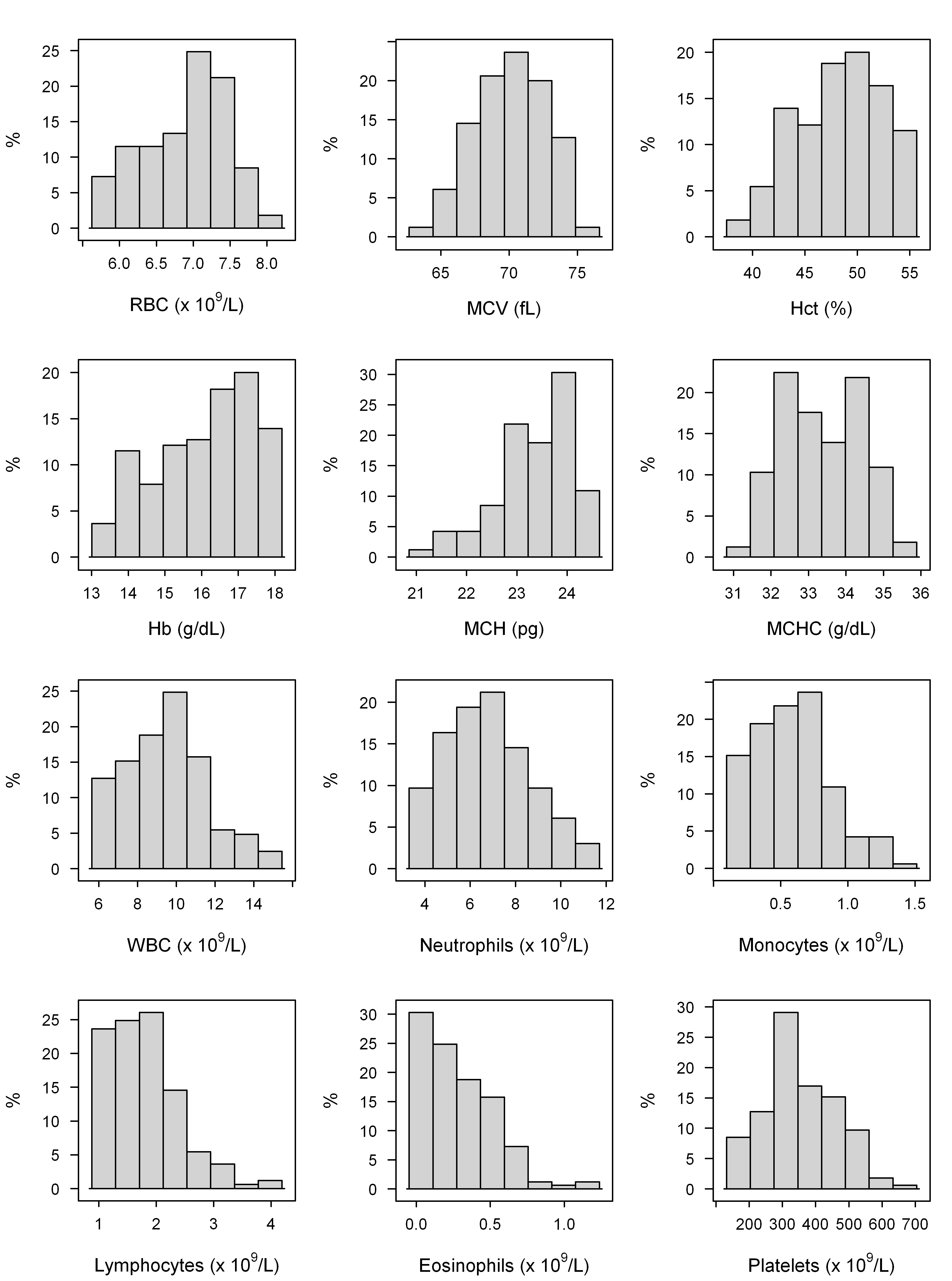

Supplement: Figure S13 — Histograms of the hematological data for the Staffordshire bull terrier (n=165). (TIF) [file pone.0081288.s013.tif]

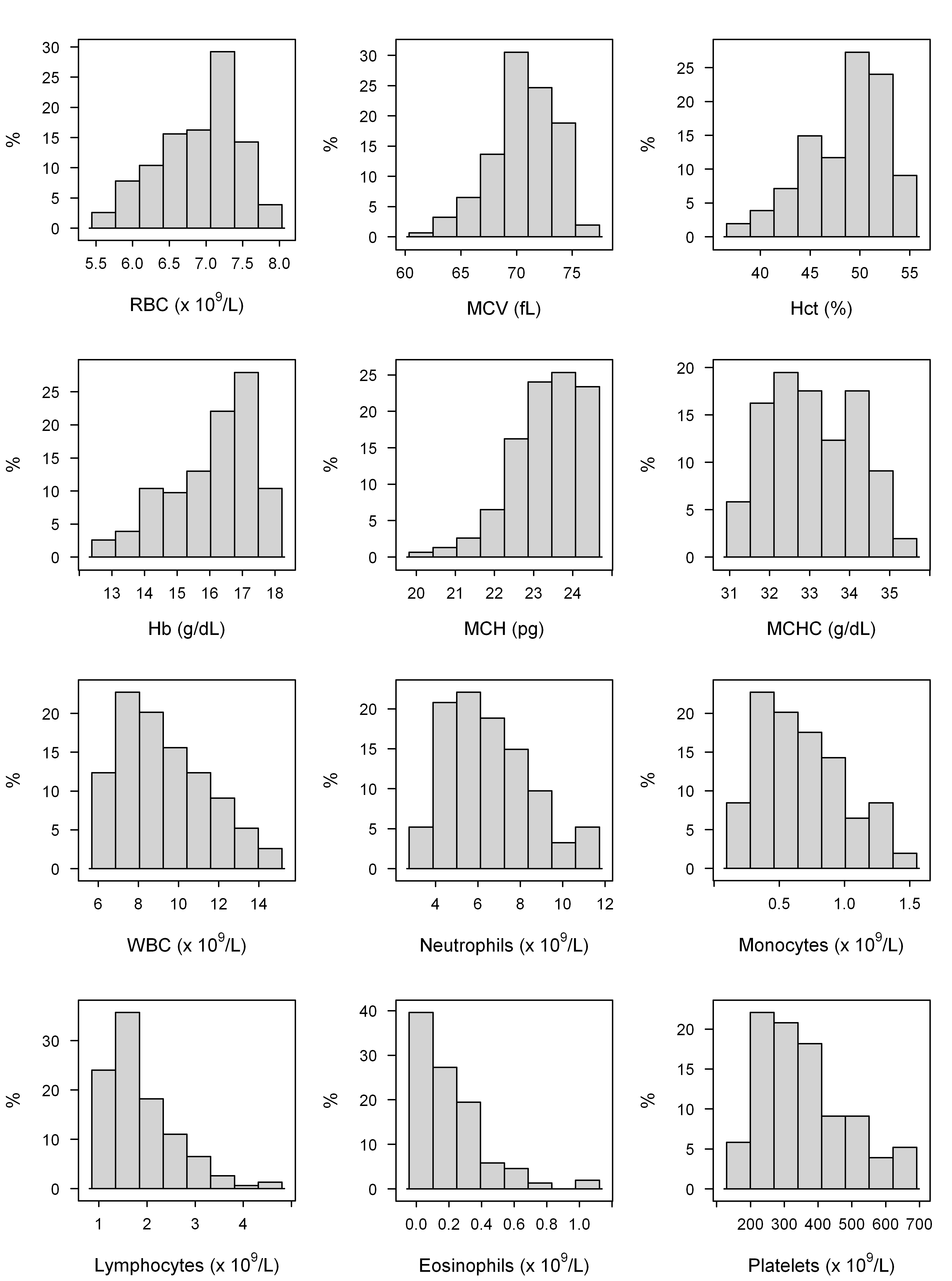

Supplement: Figure S14 — Histograms of the hematological data for the Yorkshire terrier (n=154). (TIF) [file pone.0081288.s014.tif]

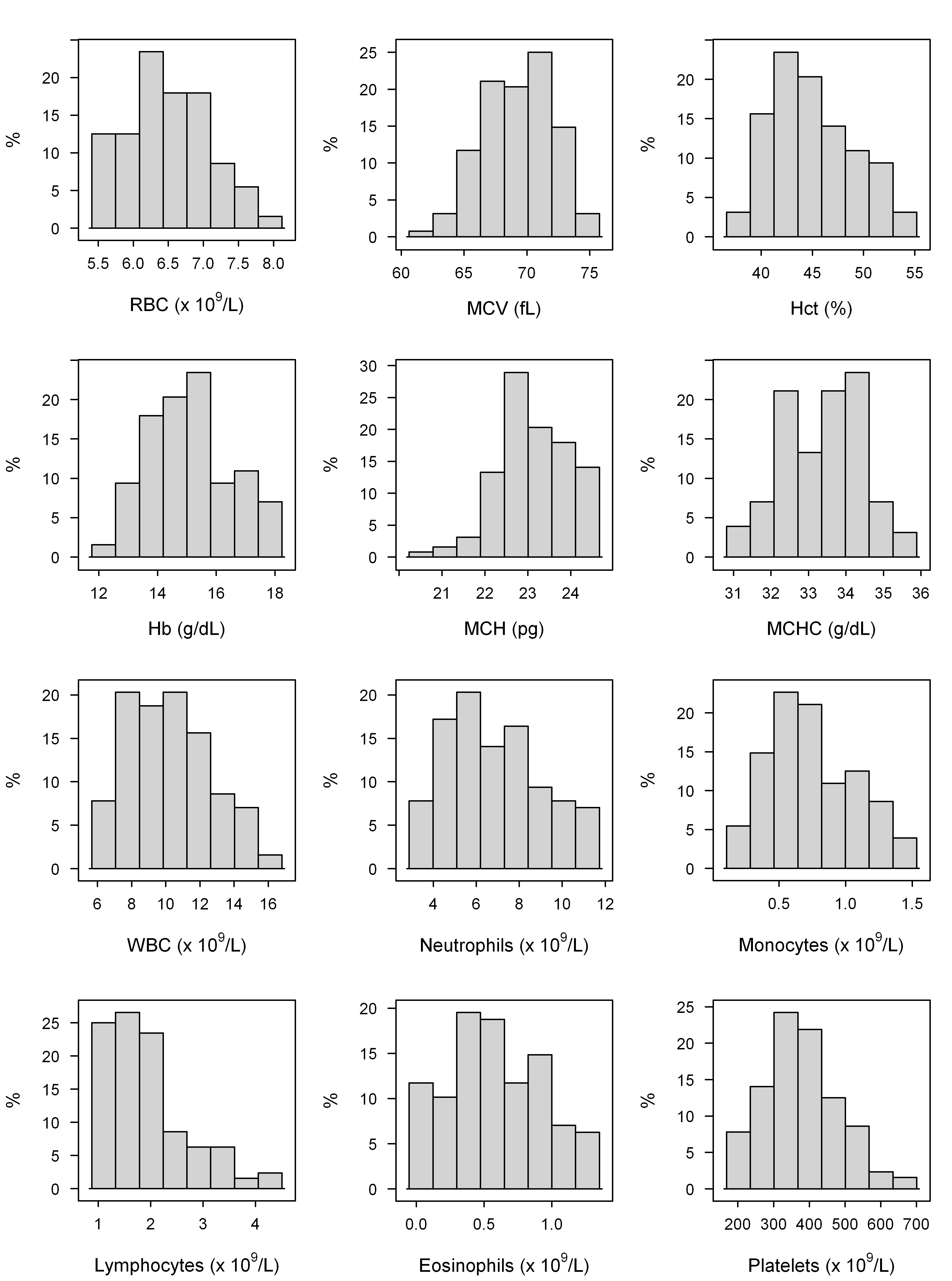

Supplement: Figure S15 — Histograms of the hematological data for the Rottweiler (n=128). (TIF) [file pone.0081288.s015.tif]
