# Supplementary material for: Breed-Specific Hematological Phenotypes in the Dog: A Natural Resource for the Genetic Dissection of Hematological Parameters in a Mammalian Species
Source: PLoS One. 2013 Nov 25;8(11):e81288. doi: 10.1371/journal.pone.0081288 (PMC3840015; doi:10.1371/journal.pone.0081288)
Supplement: Table S1 — Descriptive statistics – red blood cell concentration§. § Unit of measurement: x 1012/L; SD = standard deviation; IQR = interquartile range; Min. = minimum value recorded; Max. = maximum value recorded. (DOC) [file pone.0081288.s016.doc]

| **Breed** | **N** | **Mean** | **SD** | **Median** | **IQR** | **Min.** | **Max.** |
| --- | --- | --- | --- | --- | --- | --- | --- |
| Mixed breed | 580 | 6.79 | 0.56 | 6.80 | 0.84 | 5.51 | 8.20 |
|  |  |  |  |  |  |  |  |
| **Ancient** |  |  |  |  |  |  |  |
| Akita | 17 | 7.20 | 0.73 | 7.30 | 1.24 | 5.85 | 8.28 |
| Chow chow | 11 | 7.27 | 0.48 | 7.31 | 0.59 | 6.44 | 8.14 |
| Maltese terrier | 23 | 6.91 | 0.47 | 6.94 | 0.65 | 6.02 | 7.71 |
| Shar pei | 42 | 7.10 | 0.62 | 7.15 | 0.91 | 5.75 | 8.01 |
| Siberian husky | 26 | 6.40 | 0.59 | 6.44 | 0.83 | 5.51 | 7.76 |
| Tibetan terrier | 35 | 7.00 | 0.71 | 6.93 | 1.29 | 5.56 | 8.32 |
|  |  |  |  |  |  |  |  |
| **Toy** |  |  |  |  |  |  |  |
| Chihuahua | 18 | 6.65 | 0.56 | 6.51 | 0.95 | 5.76 | 7.56 |
| Pekingese | 17 | 6.69 | 0.61 | 6.55 | 0.77 | 5.74 | 7.77 |
| Pomeranian | 23 | 6.89 | 0.47 | 6.83 | 0.59 | 6.12 | 7.95 |
| Pug | 28 | 6.55 | 0.56 | 6.49 | 0.76 | 5.60 | 7.91 |
| Shih tzu | 92 | 6.75 | 0.59 | 6.81 | 0.85 | 5.60 | 8.17 |
|  |  |  |  |  |  |  |  |
| **Working** |  |  |  |  |  |  |  |
| Dobermann | 77 | 6.84 | 0.65 | 6.86 | 0.8 | 5.57 | 8.45 |
| German shepherd dog | 346 | 6.95 | 0.57 | 7.01 | 0.76 | 5.53 | 8.33 |
| Giant schnauzer | 19 | 6.89 | 0.67 | 6.96 | 1.03 | 5.72 | 7.84 |
| Miniature Schnauzer | 37 | 6.63 | 0.57 | 6.72 | 0.93 | 5.56 | 7.67 |
| Schnauzer | 13 | 6.64 | 0.54 | 6.57 | 0.78 | 5.89 | 7.62 |
|  |  |  |  |  |  |  |  |
| **Sight hound** |  |  |  |  |  |  |  |
| Deerhound | 10 | 7.27 | 0.35 | 7.26 | 0.35 | 6.63 | 7.82 |
| Greyhound | 10 | 7.68 | 0.46 | 7.69 | 0.69 | 7.10 | 8.37 |
| Irish wolfhound | 13 | 6.56 | 0.59 | 6.69 | 0.90 | 5.61 | 7.37 |
|  |  |  |  |  |  |  |  |
| **Mastiff-like** |  |  |  |  |  |  |  |
| Boston terrier | 10 | 6.62 | 0.44 | 6.78 | 0.66 | 5.87 | 7.09 |
| Boxer | 351 | 6.82 | 0.56 | 6.92 | 0.83 | 5.51 | 7.81 |
| Bull mastiff | 46 | 6.65 | 0.55 | 6.66 | 0.78 | 5.51 | 7.79 |
| Bulldog | 16 | 6.65 | 0.51 | 6.63 | 0.81 | 5.65 | 7.50 |
| Dogue de Bordeaux | 31 | 6.75 | 0.46 | 6.77 | 0.63 | 5.90 | 7.78 |
| English bull terrier | 53 | 6.73 | 0.58 | 6.71 | 0.77 | 5.54 | 8.01 |
| Mastiff | 23 | 6.85 | 0.73 | 7.01 | 0.66 | 5.50 | 7.97 |
| Staffordshire bull terrier | 165 | 6.89 | 0.56 | 7.03 | 0.89 | 5.72 | 8.11 |
|  |  |  |  |  |  |  |  |
| **Retriever/other Mastiff-like** |  |  |  |  |  |  |  |
| Bernese mountan dog | 40 | 6.63 | 0.56 | 6.66 | 0.85 | 5.57 | 7.63 |
| Flat-coated retriever | 44 | 6.46 | 0.52 | 6.52 | 0.59 | 5.50 | 7.94 |
| Golden retriever | 171 | 6.42 | 0.49 | 6.41 | 0.69 | 5.51 | 7.99 |
| Great dane | 41 | 7.05 | 0.63 | 7.26 | 0.99 | 5.56 | 8.16 |
| Labrador retriever | 761 | 6.61 | 0.53 | 6.62 | 0.80 | 5.50 | 7.91 |
| Leonberger | 20 | 6.44 | 0.44 | 6.33 | 0.54 | 5.81 | 7.53 |
| Newfoundland | 33 | 6.41 | 0.45 | 6.43 | 0.51 | 5.50 | 7.18 |
| Rottweiler | 128 | 6.52 | 0.60 | 6.47 | 0.82 | 5.51 | 8.02 |
| Saint Bernard | 24 | 6.49 | 0.53 | 6.53 | 0.79 | 5.66 | 7.47 |
|  |  |  |  |  |  |  |  |
| **Herding** |  |  |  |  |  |  |  |
| Bearded collie | 23 | 6.90 | 0.44 | 6.97 | 0.59 | 6.14 | 7.69 |
| Border collie | 146 | 6.59 | 0.61 | 6.55 | 1.03 | 5.51 | 7.88 |
| Old English sheepdog | 27 | 6.64 | 0.68 | 6.57 | 1.04 | 5.51 | 7.74 |
| Rough collie | 15 | 6.70 | 0.72 | 6.49 | 1.21 | 5.65 | 7.72 |
| Shetland sheepdog | 26 | 6.69 | 0.72 | 6.63 | 1.06 | 5.50 | 7.85 |
|  |  |  |  |  |  |  |  |
| **Terrier** |  |  |  |  |  |  |  |
| Airedale | 30 | 6.63 | 0.52 | 6.66 | 0.61 | 5.77 | 7.60 |
| Border terrier | 56 | 6.60 | 0.60 | 6.63 | 0.85 | 5.51 | 7.98 |
| Cairn terrier | 40 | 6.70 | 0.59 | 6.81 | 0.84 | 5.53 | 7.96 |
| Fox terrier | 13 | 6.99 | 0.46 | 7.11 | 0.46 | 5.90 | 7.74 |
| Norfolk terrier | 16 | 6.77 | 0.61 | 6.76 | 0.99 | 5.68 | 7.69 |
| Scottish terrier | 18 | 7.08 | 0.59 | 7.05 | 0.84 | 5.95 | 8.06 |
| West Highland white terrier | 199 | 6.76 | 0.57 | 6.76 | 0.82 | 5.51 | 8.24 |
| Yorkshire terrier | 154 | 6.89 | 0.54 | 7.02 | 0.82 | 5.54 | 7.94 |
|  |  |  |  |  |  |  |  |
| **Scent hound** |  |  |  |  |  |  |  |
| Basset hound | 20 | 7.06 | 0.47 | 7.18 | 0.42 | 5.68 | 7.74 |
| Beagle | 116 | 6.85 | 0.58 | 6.89 | 0.83 | 5.66 | 8.34 |
| Dachshund | 64 | 7.12 | 0.55 | 7.24 | 0.87 | 5.83 | 8.28 |
| Miniature dachshund | 15 | 7.19 | 0.67 | 7.34 | 0.64 | 5.85 | 8.10 |
| Rhodesian ridgeback | 33 | 7.12 | 0.58 | 7.32 | 0.72 | 5.89 | 8.32 |
|  |  |  |  |  |  |  |  |
| **Spaniel/Pointer** |  |  |  |  |  |  |  |
| American cocker spaniel | 12 | 6.71 | 0.43 | 6.79 | 0.74 | 5.96 | 7.23 |
| Cavalier King Charles spaniel | 280 | 6.23 | 0.44 | 6.16 | 0.59 | 5.50 | 7.45 |
| Cocker spaniel | 227 | 6.61 | 0.59 | 6.59 | 0.90 | 5.51 | 8.21 |
| English setter | 19 | 6.85 | 0.57 | 6.90 | 0.63 | 5.56 | 7.86 |
| German shorthaired pointer | 18 | 6.86 | 0.59 | 6.91 | 0.82 | 5.75 | 7.77 |
| Gordon setter | 23 | 6.45 | 0.54 | 6.45 | 0.57 | 5.64 | 7.47 |
| Hungarian vizsla | 33 | 6.72 | 0.58 | 6.66 | 0.83 | 5.58 | 7.91 |
| Irish setter | 44 | 6.67 | 0.51 | 6.78 | 0.79 | 5.54 | 7.48 |
| Italian spinone | 42 | 6.93 | 0.62 | 6.94 | 0.69 | 5.55 | 8.17 |
| Pointer | 13 | 6.67 | 0.56 | 6.85 | 0.42 | 5.59 | 7.26 |
| Springer spaniel | 168 | 6.56 | 0.54 | 6.56 | 0.81 | 5.52 | 7.70 |
| Weimaraner | 103 | 6.95 | 0.61 | 7.07 | 0.77 | 5.50 | 8.07 |
|  |  |  |  |  |  |  |  |
| **Other** |  |  |  |  |  |  |  |
| Bichon frise | 80 | 6.95 | 0.57 | 6.89 | 0.86 | 5.73 | 8.27 |
| Dalmatian | 39 | 6.70 | 0.58 | 6.75 | 0.86 | 5.55 | 7.77 |
| Jack russell terrier | 180 | 6.77 | 0.57 | 6.78 | 0.89 | 5.54 | 8.09 |
| Labradoodle | 16 | 6.77 | 0.69 | 6.74 | 1.2 | 5.78 | 7.86 |
| Lhasa apso | 49 | 7.05 | 0.61 | 7.18 | 0.88 | 5.60 | 8.15 |
| Miniature poodle | 19 | 6.95 | 0.55 | 7.18 | 0.53 | 5.60 | 7.50 |
| Samoyed | 25 | 6.67 | 0.57 | 6.81 | 0.73 | 5.54 | 7.51 |
| Standard poodle | 24 | 6.57 | 0.52 | 6.72 | 0.74 | 5.72 | 7.32 |
| Toy poodle | 15 | 6.97 | 0.62 | 7.01 | 0.83 | 5.88 | 8.08 |
