# Supplementary material for: Breed-Specific Hematological Phenotypes in the Dog: A Natural Resource for the Genetic Dissection of Hematological Parameters in a Mammalian Species
Source: PLoS One. 2013 Nov 25;8(11):e81288. doi: 10.1371/journal.pone.0081288 (PMC3840015; doi:10.1371/journal.pone.0081288)
Supplement: Table S2 — Descriptive statistics – mean corpuscular volume§. § Unit of measurement: fL; SD = standard deviation; IQR = interquartile range; Min. = minimum value recorded; Max. = maximum value recorded. (DOC) [file pone.0081288.s017.doc]

| **Breed** | **N** | **Mean** | **SD** | **Median** | **IQR** | **Min.** | **Max.** |
| --- | --- | --- | --- | --- | --- | --- | --- |
| Mixed breed | 580 | 70.13 | 2.73 | 70.10 | 3.73 | 61.30 | 77.00 |
|  |  |  |  |  |  |  |  |
| **Ancient** |  |  |  |  |  |  |  |
| Akita | 17 | 63.65 | 2.67 | 62.70 | 2.70 | 60.00 | 71.10 |
| Chow chow | 11 | 65.32 | 3.12 | 66.30 | 5.90 | 61.10 | 69.00 |
| Maltese terrier | 23 | 71.19 | 3.22 | 72.10 | 4.20 | 63.90 | 75.20 |
| Shar pei | 42 | 66.56 | 2.33 | 66.45 | 3.10 | 62.10 | 73.30 |
| Siberian husky | 26 | 70.13 | 2.46 | 69.85 | 3.38 | 64.80 | 75.10 |
| Tibetan terrier | 35 | 67.33 | 3.07 | 67.10 | 2.80 | 61.30 | 74.70 |
|  |  |  |  |  |  |  |  |
| **Toy** |  |  |  |  |  |  |  |
| Chihuahua | 18 | 70.99 | 2.30 | 70.95 | 1.95 | 66.40 | 75.50 |
| Pekingese | 17 | 68.78 | 2.07 | 68.60 | 3.60 | 65.30 | 71.80 |
| Pomeranian | 23 | 68.56 | 2.61 | 68.20 | 3.20 | 62.70 | 72.90 |
| Pug | 28 | 69.21 | 3.04 | 69.25 | 3.30 | 61.10 | 75.20 |
| Shih tzu | 92 | 69.48 | 2.80 | 69.50 | 3.43 | 62.60 | 75.50 |
|  |  |  |  |  |  |  |  |
| **Working** |  |  |  |  |  |  |  |
| Dobermann | 77 | 70.00 | 3.05 | 70.30 | 4.20 | 61.40 | 76.00 |
| German shepherd dog | 346 | 69.56 | 2.75 | 69.65 | 4.00 | 60.90 | 76.70 |
| Giant schnauzer | 19 | 68.15 | 2.59 | 67.90 | 3.55 | 63.90 | 72.60 |
| Miniature Schnauzer | 37 | 71.15 | 3.14 | 71.40 | 3.10 | 63.40 | 76.60 |
| Schnauzer | 13 | 69.35 | 3.23 | 70.00 | 3.70 | 63.20 | 73.60 |
|  |  |  |  |  |  |  |  |
| **Sight hound** |  |  |  |  |  |  |  |
| Deerhound | 10 | 68.61 | 2.98 | 68.20 | 1.10 | 63.80 | 75.20 |
| Greyhound | 10 | 69.11 | 3.58 | 69.35 | 5.98 | 63.80 | 74.20 |
| Irish wolfhound | 13 | 69.83 | 2.83 | 69.70 | 5.00 | 65.90 | 74.80 |
|  |  |  |  |  |  |  |  |
| **Mastiff-like** |  |  |  |  |  |  |  |
| Boston terrier | 10 | 70.35 | 2.98 | 71.20 | 5.05 | 66.30 | 74.90 |
| Boxer | 351 | 70.11 | 2.57 | 70.20 | 3.10 | 61.70 | 77.00 |
| Bull mastiff | 46 | 70.26 | 2.30 | 70.65 | 3.10 | 65.70 | 74.00 |
| Bulldog | 16 | 72.96 | 2.51 | 73.75 | 2.35 | 66.60 | 76.00 |
| Dogue de Bordeaux | 31 | 70.80 | 2.50 | 70.50 | 3.10 | 66.00 | 75.50 |
| English bull terrier | 53 | 71.32 | 2.61 | 71.50 | 3.40 | 64.50 | 75.60 |
| Mastiff | 23 | 70.23 | 3.16 | 70.20 | 4.55 | 65.70 | 76.90 |
| Staffordshire bull terrier | 165 | 70.04 | 2.61 | 70.20 | 3.70 | 63.20 | 76.10 |
|  |  |  |  |  |  |  |  |
| **Retriever/other Mastiff-like** |  |  |  |  |  |  |  |
| Bernese mountan dog | 40 | 70.27 | 2.47 | 70.60 | 3.43 | 64.70 | 74.80 |
| Flat-coated retriever | 44 | 69.50 | 2.68 | 69.15 | 3.55 | 61.60 | 75.20 |
| Golden retriever | 171 | 71.82 | 2.76 | 72.20 | 4.30 | 64.10 | 76.50 |
| Great dane | 41 | 69.62 | 2.45 | 69.90 | 3.40 | 64.50 | 75.30 |
| Labrador retriever | 761 | 70.02 | 2.68 | 70.10 | 3.70 | 61.80 | 76.70 |
| Leonberger | 20 | 68.88 | 2.59 | 69.00 | 2.90 | 63.30 | 73.90 |
| Newfoundland | 33 | 71.05 | 2.43 | 70.90 | 3.70 | 66.50 | 75.60 |
| Rottweiler | 128 | 69.26 | 2.73 | 69.50 | 3.90 | 61.20 | 75.20 |
| Saint Bernard | 24 | 69.21 | 2.66 | 69.50 | 2.82 | 60.70 | 73.60 |
|  |  |  |  |  |  |  |  |
| **Herding** |  |  |  |  |  |  |  |
| Bearded collie | 23 | 71.00 | 2.52 | 71.30 | 3.20 | 65.90 | 75.30 |
| Border collie | 146 | 70.57 | 2.80 | 70.70 | 3.05 | 61.80 | 76.70 |
| Old English sheepdog | 27 | 70.21 | 3.74 | 71.30 | 5.45 | 61.50 | 74.50 |
| Rough collie | 15 | 70.11 | 1.79 | 70.30 | 2.25 | 67.30 | 73.90 |
| Shetland sheepdog | 26 | 69.86 | 2.46 | 69.90 | 2.68 | 65.50 | 75.90 |
|  |  |  |  |  |  |  |  |
| **Terrier** |  |  |  |  |  |  |  |
| Airedale | 30 | 71.99 | 2.31 | 72.00 | 3.93 | 67.50 | 76.90 |
| Border terrier | 56 | 70.55 | 2.86 | 70.75 | 4.33 | 64.40 | 75.80 |
| Cairn terrier | 40 | 70.36 | 3.00 | 70.45 | 3.98 | 64.00 | 76.30 |
| Fox terrier | 13 | 70.88 | 3.32 | 70.60 | 3.70 | 64.10 | 76.20 |
| Norfolk terrier | 16 | 69.60 | 3.71 | 70.10 | 5.80 | 61.60 | 73.70 |
| Scottish terrier | 18 | 69.29 | 2.54 | 69.35 | 3.05 | 64.00 | 74.40 |
| West Highland white terrier | 199 | 70.65 | 2.74 | 70.90 | 3.65 | 60.20 | 76.30 |
| Yorkshire terrier | 154 | 70.54 | 2.90 | 70.55 | 3.35 | 61.00 | 76.80 |
|  |  |  |  |  |  |  |  |
| **Scent hound** |  |  |  |  |  |  |  |
| Basset hound | 20 | 70.13 | 2.37 | 70.05 | 2.35 | 65.00 | 74.10 |
| Beagle | 116 | 70.07 | 2.52 | 70.10 | 3.47 | 64.20 | 76.60 |
| Dachshund | 64 | 69.28 | 3.21 | 69.40 | 3.83 | 62.20 | 76.80 |
| Miniature dachshund | 15 | 66.47 | 2.27 | 66.30 | 2.25 | 62.70 | 70.20 |
| Rhodesian ridgeback | 33 | 70.55 | 2.72 | 70.90 | 3.10 | 62.50 | 74.30 |
|  |  |  |  |  |  |  |  |
| **Spaniel/Pointer** |  |  |  |  |  |  |  |
| American cocker spaniel | 12 | 70.43 | 1.55 | 70.65 | 2.28 | 68.20 | 72.70 |
| Cavalier King Charles spaniel | 280 | 70.86 | 2.38 | 70.70 | 3.30 | 63.80 | 75.90 |
| Cocker spaniel | 227 | 69.58 | 2.68 | 69.70 | 3.65 | 60.00 | 75.60 |
| English setter | 19 | 70.11 | 2.93 | 70.50 | 3.40 | 63.60 | 74.00 |
| German shorthaired pointer | 18 | 71.27 | 2.61 | 71.10 | 2.20 | 63.90 | 75.80 |
| Gordon setter | 23 | 70.30 | 1.94 | 70.50 | 2.90 | 67.20 | 73.90 |
| Hungarian vizsla | 33 | 70.33 | 2.50 | 70.40 | 4.10 | 66.10 | 75.20 |
| Irish setter | 44 | 71.55 | 2.35 | 71.45 | 2.75 | 65.20 | 75.60 |
| Italian spinone | 42 | 68.47 | 3.22 | 68.60 | 4.45 | 62.40 | 74.40 |
| Pointer | 13 | 71.19 | 2.50 | 72.20 | 4.30 | 66.60 | 74.10 |
| Springer spaniel | 168 | 70.90 | 2.81 | 70.85 | 4.33 | 63.90 | 76.90 |
| Weimaraner | 103 | 68.37 | 3.01 | 68.20 | 4.05 | 60.00 | 76.90 |
|  |  |  |  |  |  |  |  |
| **Other** |  |  |  |  |  |  |  |
| Bichon frise | 80 | 68.84 | 3.26 | 68.75 | 4.93 | 61.50 | 75.80 |
| Dalmatian | 39 | 71.37 | 2.72 | 71.60 | 4.40 | 65.60 | 76.00 |
| Jack russell terrier | 180 | 70.31 | 3.04 | 70.45 | 4.05 | 61.20 | 76.30 |
| Labradoodle | 16 | 69.68 | 2.34 | 69.95 | 3.05 | 65.70 | 74.60 |
| Lhasa apso | 49 | 66.98 | 2.78 | 67.00 | 4.70 | 61.70 | 73.10 |
| Miniature poodle | 19 | 69.38 | 2.05 | 69.20 | 2.35 | 66.00 | 73.30 |
| Samoyed | 25 | 70.63 | 3.35 | 70.40 | 4.80 | 62.10 | 76.10 |
| Standard poodle | 24 | 70.47 | 3.39 | 70.40 | 4.22 | 62.90 | 75.70 |
| Toy poodle | 15 | 70.45 | 3.10 | 70.70 | 4.50 | 65.90 | 76.70 |
