# Supplementary material for: Breed-Specific Hematological Phenotypes in the Dog: A Natural Resource for the Genetic Dissection of Hematological Parameters in a Mammalian Species
Source: PLoS One. 2013 Nov 25;8(11):e81288. doi: 10.1371/journal.pone.0081288 (PMC3840015; doi:10.1371/journal.pone.0081288)
Supplement: Table S3 — Descriptive statistics – hematocrit§. § Stated as a percentage; SD = standard deviation; IQR = interquartile range; Min. = minimum value recorded; Max. = maximum value recorded. (DOC) [file pone.0081288.s018.doc]

| **Breed** | **N** | **Mean** | **SD** | **Median** | **IQR** | **Min.** | **Max.** |
| --- | --- | --- | --- | --- | --- | --- | --- |
| Mixed breed | 580 | 47.64 | 3.99 | 47.85 | 6.05 | 37.60 | 55.00 |
|  |  |  |  |  |  |  |  |
| **Ancient** |  |  |  |  |  |  |  |
| Akita | 17 | 45.78 | 4.23 | 46.20 | 8.00 | 39.60 | 52.00 |
| Chow chow | 11 | 47.50 | 3.78 | 47.60 | 4.65 | 40.20 | 52.80 |
| Maltese terrier | 23 | 49.15 | 2.99 | 50.00 | 5.10 | 44.10 | 53.70 |
| Shar pei | 42 | 47.19 | 3.80 | 47.50 | 6.37 | 39.80 | 54.10 |
| Siberian husky | 26 | 44.86 | 4.35 | 43.85 | 6.20 | 37.50 | 53.60 |
| Tibetan terrier | 35 | 47.10 | 4.74 | 48.20 | 7.35 | 37.40 | 54.90 |
|  |  |  |  |  |  |  |  |
| **Toy** |  |  |  |  |  |  |  |
| Chihuahua | 18 | 47.13 | 3.40 | 46.30 | 6.15 | 42.30 | 53.20 |
| Pekingese | 17 | 45.95 | 4.10 | 45.10 | 5.50 | 39.40 | 53.30 |
| Pomeranian | 23 | 47.20 | 2.98 | 46.90 | 5.00 | 42.50 | 52.20 |
| Pug | 28 | 45.24 | 3.56 | 45.55 | 3.73 | 37.50 | 50.50 |
| Shih tzu | 92 | 46.87 | 4.27 | 47.10 | 6.03 | 37.40 | 54.90 |
|  |  |  |  |  |  |  |  |
| **Working** |  |  |  |  |  |  |  |
| Dobermann | 77 | 47.79 | 4.15 | 48.00 | 5.90 | 38.60 | 55.00 |
| German shepherd dog | 346 | 48.30 | 3.82 | 48.75 | 5.18 | 37.70 | 55.00 |
| Giant schnauzer | 19 | 46.87 | 4.30 | 48.20 | 6.00 | 39.30 | 53.70 |
| Miniature Schnauzer | 37 | 47.11 | 3.83 | 47.10 | 5.80 | 37.40 | 53.40 |
| Schnauzer | 13 | 45.98 | 3.31 | 45.30 | 3.10 | 40.70 | 52.10 |
|  |  |  |  |  |  |  |  |
| **Sight hound** |  |  |  |  |  |  |  |
| Deerhound | 10 | 49.80 | 1.73 | 49.45 | 2.70 | 47.50 | 52.40 |
| Greyhound | 10 | 53.02 | 3.25 | 53.80 | 3.53 | 47.50 | 56.80 |
| Irish wolfhound | 13 | 45.77 | 4.17 | 46.10 | 4.60 | 37.00 | 53.40 |
|  |  |  |  |  |  |  |  |
| **Mastiff-like** |  |  |  |  |  |  |  |
| Boston terrier | 10 | 46.68 | 4.41 | 48.55 | 6.40 | 38.90 | 51.60 |
| Boxer | 351 | 47.82 | 4.01 | 48.60 | 5.35 | 37.50 | 55.00 |
| Bull mastiff | 46 | 46.73 | 4.08 | 46.80 | 6.08 | 37.50 | 53.70 |
| Bulldog | 16 | 48.46 | 3.43 | 49.30 | 4.70 | 42.50 | 53.80 |
| Dogue de Bordeaux | 31 | 47.77 | 3.53 | 48.50 | 4.85 | 39.00 | 54.90 |
| English bull terrier | 53 | 47.99 | 4.31 | 47.90 | 6.50 | 38.80 | 54.80 |
| Mastiff | 23 | 48.00 | 4.67 | 48.20 | 5.65 | 38.40 | 54.90 |
| Staffordshire bull terrier | 165 | 48.24 | 4.09 | 48.70 | 6.60 | 38.20 | 55.00 |
|  |  |  |  |  |  |  |  |
| **Retriever/other Mastiff-like** |  |  |  |  |  |  |  |
| Bernese mountan dog | 40 | 46.60 | 3.98 | 47.20 | 6.65 | 40.20 | 53.80 |
| Flat-coated retriever | 44 | 44.84 | 3.73 | 44.20 | 5.43 | 37.80 | 51.60 |
| Golden retriever | 171 | 46.11 | 3.75 | 46.00 | 5.70 | 38.00 | 54.80 |
| Great dane | 41 | 49.05 | 4.59 | 50.40 | 6.20 | 39.50 | 54.70 |
| Labrador retriever | 761 | 46.25 | 3.92 | 46.30 | 6.00 | 37.00 | 54.70 |
| Leonberger | 20 | 44.38 | 3.40 | 43.25 | 3.90 | 40.20 | 53.00 |
| Newfoundland | 33 | 45.48 | 3.06 | 45.70 | 3.80 | 40.90 | 53.70 |
| Rottweiler | 128 | 45.09 | 4.09 | 45.10 | 6.30 | 37.30 | 54.50 |
| Saint Bernard | 24 | 44.90 | 4.15 | 44.80 | 5.85 | 37.60 | 54.90 |
|  |  |  |  |  |  |  |  |
| **Herding** |  |  |  |  |  |  |  |
| Bearded collie | 23 | 48.96 | 3.30 | 48.20 | 4.55 | 43.00 | 54.80 |
| Border collie | 146 | 46.50 | 4.34 | 46.85 | 6.73 | 37.30 | 54.90 |
| Old English sheepdog | 27 | 46.53 | 4.68 | 46.60 | 7.60 | 40.10 | 54.20 |
| Rough collie | 15 | 46.97 | 4.73 | 46.70 | 7.30 | 38.70 | 54.00 |
| Shetland sheepdog | 26 | 46.67 | 4.81 | 46.70 | 7.68 | 38.00 | 54.10 |
|  |  |  |  |  |  |  |  |
| **Terrier** |  |  |  |  |  |  |  |
| Airedale | 30 | 47.68 | 3.92 | 47.50 | 6.30 | 40.10 | 53.60 |
| Border terrier | 56 | 46.53 | 4.09 | 47.00 | 5.20 | 37.70 | 54.40 |
| Cairn terrier | 40 | 47.07 | 3.83 | 48.25 | 5.53 | 38.60 | 54.20 |
| Fox terrier | 13 | 49.47 | 3.46 | 49.40 | 2.40 | 40.30 | 54.40 |
| Norfolk terrier | 16 | 46.99 | 3.91 | 46.50 | 6.40 | 40.00 | 51.90 |
| Scottish terrier | 18 | 48.99 | 3.58 | 49.05 | 4.88 | 40.00 | 54.80 |
| West Highland white terrier | 199 | 47.76 | 4.06 | 47.60 | 6.10 | 37.30 | 54.90 |
| Yorkshire terrier | 154 | 48.62 | 4.06 | 49.40 | 6.33 | 37.30 | 55.00 |
|  |  |  |  |  |  |  |  |
| **Scent hound** |  |  |  |  |  |  |  |
| Basset hound | 20 | 49.47 | 3.48 | 49.90 | 3.93 | 41.20 | 55.00 |
| Beagle | 116 | 47.97 | 4.06 | 48.00 | 5.88 | 38.00 | 54.60 |
| Dachshund | 64 | 49.30 | 3.83 | 50.15 | 6.35 | 40.40 | 55.00 |
| Miniature dachshund | 15 | 47.81 | 4.62 | 49.30 | 5.80 | 40.00 | 54.10 |
| Rhodesian ridgeback | 33 | 50.16 | 3.66 | 51.50 | 5.60 | 43.20 | 54.80 |
|  |  |  |  |  |  |  |  |
| **Spaniel/Pointer** |  |  |  |  |  |  |  |
| American cocker spaniel | 12 | 47.23 | 3.30 | 47.55 | 4.97 | 40.70 | 51.20 |
| Cavalier King Charles spaniel | 280 | 44.15 | 3.44 | 43.80 | 5.10 | 37.60 | 54.00 |
| Cocker spaniel | 227 | 45.93 | 4.15 | 46.00 | 6.70 | 37.10 | 54.80 |
| English setter | 19 | 47.99 | 3.75 | 48.20 | 4.95 | 39.00 | 53.30 |
| German shorthaired pointer | 18 | 48.81 | 3.76 | 49.50 | 3.53 | 40.80 | 54.30 |
| Gordon setter | 23 | 45.37 | 4.12 | 45.20 | 4.90 | 39.70 | 52.60 |
| Hungarian vizsla | 33 | 47.23 | 4.10 | 46.70 | 6.40 | 38.40 | 54.90 |
| Irish setter | 44 | 47.71 | 3.85 | 48.35 | 5.43 | 38.20 | 53.70 |
| Italian spinone | 42 | 47.39 | 4.37 | 48.00 | 4.93 | 37.50 | 54.50 |
| Pointer | 13 | 47.46 | 4.10 | 48.30 | 4.50 | 41.10 | 52.70 |
| Springer spaniel | 168 | 46.48 | 4.09 | 46.40 | 5.95 | 37.80 | 54.90 |
| Weimaraner | 103 | 47.47 | 4.09 | 47.70 | 5.65 | 37.20 | 54.90 |
|  |  |  |  |  |  |  |  |
| **Other** |  |  |  |  |  |  |  |
| Bichon frise | 80 | 47.78 | 3.42 | 47.65 | 4.87 | 38.50 | 54.40 |
| Dalmatian | 39 | 47.83 | 4.49 | 48.70 | 6.90 | 39.30 | 54.40 |
| Jack russell terrier | 180 | 47.55 | 4.02 | 47.70 | 6.25 | 37.50 | 55.00 |
| Labradoodle | 16 | 47.16 | 4.99 | 46.35 | 8.03 | 39.50 | 54.90 |
| Lhasa apso | 49 | 47.19 | 4.21 | 47.30 | 5.80 | 38.50 | 54.00 |
| Miniature poodle | 19 | 48.17 | 3.37 | 48.90 | 3.25 | 40.70 | 53.70 |
| Samoyed | 25 | 47.10 | 4.42 | 47.10 | 5.50 | 39.00 | 54.20 |
| Standard poodle | 24 | 46.27 | 3.85 | 46.50 | 6.47 | 38.40 | 51.90 |
| Toy poodle | 15 | 49.03 | 4.52 | 49.50 | 7.70 | 42.00 | 54.40 |
