# Supplementary material for: Breed-Specific Hematological Phenotypes in the Dog: A Natural Resource for the Genetic Dissection of Hematological Parameters in a Mammalian Species
Source: PLoS One. 2013 Nov 25;8(11):e81288. doi: 10.1371/journal.pone.0081288 (PMC3840015; doi:10.1371/journal.pone.0081288)
Supplement: Table S4 — Descriptive statistics – hemoglobin§. § Unit of measurement: g/dL; SD = standard deviation; IQR = interquartile range; Min. = minimum value recorded; Max. = maximum value recorded. (DOC) [file pone.0081288.s019.doc]

| **Breed** | **N** | **Mean** | **SD** | **Median** | **IQR** | **Min.** | **Max.** |
| --- | --- | --- | --- | --- | --- | --- | --- |
| Mixed breed | 580 | 15.88 | 1.29 | 15.90 | 2.00 | 12.40 | 18.00 |
|  |  |  |  |  |  |  |  |
| **Ancient** |  |  |  |  |  |  |  |
| Akita | 17 | 15.06 | 1.26 | 14.90 | 2.50 | 13.40 | 16.80 |
| Chow chow | 11 | 15.43 | 1.23 | 15.10 | 1.60 | 13.10 | 17.30 |
| Maltese terrier | 23 | 16.19 | 0.98 | 16.60 | 1.45 | 14.00 | 17.40 |
| Shar pei | 42 | 15.52 | 1.36 | 15.75 | 2.18 | 12.80 | 17.90 |
| Siberian husky | 26 | 15.04 | 1.34 | 14.95 | 1.70 | 12.50 | 17.90 |
| Tibetan terrier | 35 | 15.70 | 1.58 | 15.80 | 2.25 | 12.30 | 17.90 |
|  |  |  |  |  |  |  |  |
| **Toy** |  |  |  |  |  |  |  |
| Chihuahua | 18 | 15.66 | 1.32 | 15.65 | 2.03 | 13.40 | 17.70 |
| Pekingese | 17 | 15.44 | 1.12 | 15.30 | 1.70 | 13.60 | 17.10 |
| Pomeranian | 23 | 15.63 | 1.10 | 15.70 | 1.65 | 13.70 | 17.70 |
| Pug | 28 | 15.45 | 1.26 | 15.40 | 1.83 | 13.20 | 17.70 |
| Shih tzu | 92 | 15.57 | 1.32 | 15.80 | 1.90 | 12.40 | 18.00 |
|  |  |  |  |  |  |  |  |
| **Working** |  |  |  |  |  |  |  |
| Dobermann | 77 | 15.81 | 1.38 | 15.90 | 2.30 | 12.80 | 17.90 |
| German shepherd dog | 346 | 16.11 | 1.27 | 16.30 | 1.88 | 12.60 | 18.00 |
| Giant schnauzer | 19 | 15.77 | 1.54 | 16.20 | 2.25 | 12.50 | 17.60 |
| Miniature Schnauzer | 37 | 15.62 | 1.36 | 15.80 | 2.00 | 13.00 | 17.90 |
| Schnauzer | 13 | 15.39 | 1.21 | 15.40 | 1.70 | 13.40 | 17.40 |
|  |  |  |  |  |  |  |  |
| **Sight hound** |  |  |  |  |  |  |  |
| Deerhound | 10 | 16.61 | 0.79 | 16.65 | 0.88 | 15.20 | 17.80 |
| Greyhound | 10 | 17.67 | 1.19 | 17.70 | 1.93 | 15.90 | 19.30 |
| Irish wolfhound | 13 | 15.15 | 1.23 | 15.40 | 2.00 | 12.70 | 16.80 |
|  |  |  |  |  |  |  |  |
| **Mastiff-like** |  |  |  |  |  |  |  |
| Boston terrier | 10 | 15.53 | 1.30 | 16.15 | 2.18 | 13.70 | 16.90 |
| Boxer | 351 | 16.00 | 1.28 | 16.20 | 1.90 | 12.20 | 18.00 |
| Bull mastiff | 46 | 15.45 | 1.18 | 15.45 | 1.63 | 13.20 | 17.40 |
| Bulldog | 16 | 16.03 | 1.14 | 16.15 | 1.45 | 13.70 | 17.50 |
| Dogue de Bordeaux | 31 | 15.83 | 1.07 | 16.20 | 1.50 | 13.70 | 17.70 |
| English bull terrier | 53 | 15.87 | 1.33 | 16.00 | 1.90 | 13.00 | 18.00 |
| Mastiff | 23 | 15.93 | 1.54 | 16.40 | 2.05 | 12.70 | 17.80 |
| Staffordshire bull terrier | 165 | 16.07 | 1.32 | 16.30 | 2.10 | 13.20 | 18.00 |
|  |  |  |  |  |  |  |  |
| **Retriever/other Mastiff-like** |  |  |  |  |  |  |  |
| Bernese mountan dog | 40 | 15.66 | 1.28 | 16.00 | 2.00 | 12.70 | 17.90 |
| Flat-coated retriever | 44 | 15.05 | 1.20 | 15.00 | 1.63 | 12.60 | 17.60 |
| Golden retriever | 171 | 15.28 | 1.15 | 15.20 | 1.65 | 12.50 | 18.00 |
| Great dane | 41 | 16.34 | 1.46 | 17.00 | 2.00 | 12.60 | 18.00 |
| Labrador retriever | 761 | 15.45 | 1.24 | 15.50 | 1.90 | 12.40 | 18.00 |
| Leonberger | 20 | 14.80 | 1.23 | 14.40 | 1.40 | 13.30 | 17.40 |
| Newfoundland | 33 | 15.19 | 1.05 | 15.30 | 1.60 | 13.20 | 17.10 |
| Rottweiler | 128 | 15.05 | 1.40 | 15.10 | 2.10 | 12.00 | 18.00 |
| Saint Bernard | 24 | 14.84 | 1.44 | 14.75 | 2.15 | 12.40 | 18.00 |
|  |  |  |  |  |  |  |  |
| **Herding** |  |  |  |  |  |  |  |
| Bearded collie | 23 | 16.41 | 0.98 | 16.70 | 1.55 | 14.70 | 17.90 |
| Border collie | 146 | 15.39 | 1.41 | 15.45 | 2.20 | 12.40 | 18.00 |
| Old English sheepdog | 27 | 15.39 | 1.50 | 15.50 | 2.45 | 12.90 | 17.60 |
| Rough collie | 15 | 15.59 | 1.73 | 15.20 | 3.00 | 13.10 | 18.00 |
| Shetland sheepdog | 26 | 15.49 | 1.53 | 15.65 | 2.00 | 12.20 | 17.80 |
|  |  |  |  |  |  |  |  |
| **Terrier** |  |  |  |  |  |  |  |
| Airedale | 30 | 15.73 | 1.29 | 15.80 | 1.85 | 13.10 | 17.60 |
| Border terrier | 56 | 15.49 | 1.33 | 15.80 | 1.80 | 12.70 | 18.00 |
| Cairn terrier | 40 | 15.63 | 1.21 | 15.75 | 1.65 | 12.50 | 17.90 |
| Fox terrier | 13 | 16.42 | 0.85 | 16.70 | 0.70 | 14.20 | 17.40 |
| Norfolk terrier | 16 | 15.49 | 1.28 | 15.80 | 2.10 | 13.60 | 17.50 |
| Scottish terrier | 18 | 16.22 | 1.04 | 16.15 | 1.28 | 14.00 | 17.60 |
| West Highland white terrier | 199 | 15.85 | 1.31 | 15.90 | 1.90 | 12.00 | 18.00 |
| Yorkshire terrier | 154 | 16.08 | 1.29 | 16.40 | 1.80 | 12.60 | 18.00 |
|  |  |  |  |  |  |  |  |
| **Scent hound** |  |  |  |  |  |  |  |
| Basset hound | 20 | 16.52 | 1.00 | 16.85 | 0.98 | 13.90 | 17.70 |
| Beagle | 116 | 15.86 | 1.31 | 15.90 | 2.00 | 13.20 | 18.00 |
| Dachshund | 64 | 16.40 | 1.17 | 16.80 | 1.83 | 13.40 | 18.00 |
| Miniature dachshund | 15 | 16.20 | 1.42 | 16.80 | 1.50 | 13.30 | 17.90 |
| Rhodesian ridgeback | 33 | 16.56 | 1.18 | 16.90 | 1.60 | 13.90 | 18.00 |
|  |  |  |  |  |  |  |  |
| **Spaniel/Pointer** |  |  |  |  |  |  |  |
| American cocker spaniel | 12 | 15.68 | 1.04 | 15.40 | 1.63 | 13.80 | 17.00 |
| Cavalier King Charles spaniel | 280 | 14.68 | 1.05 | 14.60 | 1.40 | 12.20 | 17.90 |
| Cocker spaniel | 227 | 15.42 | 1.33 | 15.50 | 2.20 | 12.30 | 18.00 |
| English setter | 19 | 15.95 | 1.26 | 15.80 | 1.95 | 13.30 | 17.60 |
| German shorthaired pointer | 18 | 16.09 | 1.15 | 16.30 | 1.63 | 13.90 | 17.60 |
| Gordon setter | 23 | 15.05 | 1.18 | 15.00 | 2.15 | 13.40 | 16.80 |
| Hungarian vizsla | 33 | 15.68 | 1.28 | 15.70 | 1.80 | 13.30 | 17.80 |
| Irish setter | 44 | 15.80 | 1.28 | 16.10 | 2.18 | 12.60 | 18.00 |
| Italian spinone | 42 | 15.78 | 1.23 | 15.95 | 1.08 | 12.10 | 18.00 |
| Pointer | 13 | 15.72 | 1.20 | 16.10 | 0.80 | 13.60 | 17.20 |
| Springer spaniel | 168 | 15.49 | 1.23 | 15.55 | 1.80 | 12.70 | 17.90 |
| Weimaraner | 103 | 15.71 | 1.31 | 15.80 | 1.65 | 12.30 | 18.00 |
|  |  |  |  |  |  |  |  |
| **Other** |  |  |  |  |  |  |  |
| Bichon frise | 80 | 15.85 | 1.17 | 15.75 | 1.55 | 13.10 | 18.00 |
| Dalmatian | 39 | 15.73 | 1.38 | 16.00 | 2.45 | 13.30 | 17.80 |
| Jack russell terrier | 180 | 15.83 | 1.28 | 15.80 | 1.93 | 12.90 | 18.00 |
| Labradoodle | 16 | 15.91 | 1.46 | 15.80 | 2.45 | 13.70 | 17.90 |
| Lhasa apso | 49 | 15.82 | 1.32 | 16.10 | 1.90 | 12.90 | 17.90 |
| Miniature poodle | 19 | 16.18 | 1.23 | 16.60 | 1.20 | 12.90 | 17.90 |
| Samoyed | 25 | 15.55 | 1.33 | 16.00 | 2.00 | 13.30 | 17.50 |
| Standard poodle | 24 | 15.39 | 1.35 | 15.70 | 2.30 | 12.60 | 17.10 |
| Toy poodle | 15 | 16.33 | 1.40 | 16.70 | 1.80 | 13.70 | 17.90 |
