# Supplementary material for: Breed-Specific Hematological Phenotypes in the Dog: A Natural Resource for the Genetic Dissection of Hematological Parameters in a Mammalian Species
Source: PLoS One. 2013 Nov 25;8(11):e81288. doi: 10.1371/journal.pone.0081288 (PMC3840015; doi:10.1371/journal.pone.0081288)
Supplement: Table S5 — Descriptive statistics – mean corpuscular hemoglobin§. § Unit of measurement: pg; SD = standard deviation; IQR = interquartile range; Min. = minimum value recorded; Max. = maximum value recorded. (DOC) [file pone.0081288.s020.doc]

| **Breed** | **N** | **Mean** | **SD** | **Median** | **IQR** | **Min.** | **Max.** |
| --- | --- | --- | --- | --- | --- | --- | --- |
| Mixed breed | 580 | 23.38 | 0.83 | 23.60 | 1.00 | 19.80 | 24.50 |
|  |  |  |  |  |  |  |  |
| **Ancient** |  |  |  |  |  |  |  |
| Akita | 17 | 20.96 | 1.13 | 20.80 | 1.00 | 19.60 | 24.20 |
| Chow chow | 11 | 21.24 | 0.83 | 21.50 | 1.25 | 19.80 | 22.30 |
| Maltese terrier | 23 | 23.44 | 0.82 | 23.50 | 0.95 | 21.60 | 24.50 |
| Shar pei | 42 | 21.88 | 0.78 | 21.85 | 0.90 | 20.30 | 23.80 |
| Siberian husky | 26 | 23.54 | 0.71 | 23.75 | 1.23 | 22.10 | 24.50 |
| Tibetan terrier | 35 | 22.45 | 1.13 | 22.50 | 1.55 | 20.20 | 24.30 |
|  |  |  |  |  |  |  |  |
| **Toy** |  |  |  |  |  |  |  |
| Chihuahua | 18 | 23.58 | 0.86 | 23.80 | 1.05 | 21.30 | 24.50 |
| Pekingese | 17 | 23.12 | 0.83 | 23.20 | 1.50 | 21.90 | 24.50 |
| Pomeranian | 23 | 22.70 | 0.93 | 22.60 | 1.40 | 20.90 | 24.30 |
| Pug | 28 | 23.63 | 0.90 | 23.80 | 0.98 | 21.30 | 24.50 |
| Shih tzu | 92 | 23.09 | 0.88 | 23.30 | 1.30 | 21.00 | 24.50 |
|  |  |  |  |  |  |  |  |
| **Working** |  |  |  |  |  |  |  |
| Dobermann | 77 | 23.15 | 0.91 | 23.40 | 1.10 | 20.50 | 24.50 |
| German shepherd dog | 346 | 23.19 | 0.84 | 23.40 | 1.20 | 20.50 | 24.50 |
| Giant schnauzer | 19 | 22.92 | 0.89 | 22.90 | 1.10 | 20.40 | 24.30 |
| Miniature Schnauzer | 37 | 23.58 | 1.03 | 23.90 | 1.20 | 20.10 | 24.50 |
| Schnauzer | 13 | 23.18 | 0.85 | 23.30 | 1.10 | 21.40 | 24.50 |
|  |  |  |  |  |  |  |  |
| **Sight hound** |  |  |  |  |  |  |  |
| Deerhound | 10 | 22.87 | 1.06 | 23.15 | 0.45 | 20.20 | 24.00 |
| Greyhound | 10 | 23.02 | 1.31 | 23.15 | 1.15 | 20.20 | 24.40 |
| Irish wolfhound | 13 | 23.10 | 0.74 | 22.80 | 1.20 | 22.10 | 24.20 |
|  |  |  |  |  |  |  |  |
| **Mastiff-like** |  |  |  |  |  |  |  |
| Boston terrier | 10 | 23.44 | 0.87 | 23.45 | 0.57 | 21.30 | 24.40 |
| Boxer | 351 | 23.47 | 0.68 | 23.50 | 0.95 | 20.40 | 24.50 |
| Bull mastiff | 46 | 23.25 | 0.77 | 23.40 | 0.77 | 21.40 | 24.50 |
| Bulldog | 16 | 24.11 | 0.39 | 24.25 | 0.40 | 23.30 | 24.50 |
| Dogue de Bordeaux | 31 | 23.47 | 0.68 | 23.50 | 0.95 | 21.60 | 24.50 |
| English bull terrier | 53 | 23.59 | 0.70 | 23.80 | 0.90 | 21.30 | 24.50 |
| Mastiff | 23 | 23.31 | 0.94 | 23.40 | 1.50 | 21.10 | 24.50 |
| Staffordshire bull terrier | 165 | 23.35 | 0.75 | 23.50 | 1.00 | 21.00 | 24.50 |
|  |  |  |  |  |  |  |  |
| **Retriever/other Mastiff-like** |  |  |  |  |  |  |  |
| Bernese mountan dog | 40 | 23.62 | 0.55 | 23.70 | 0.80 | 22.40 | 24.50 |
| Flat-coated retriever | 44 | 23.33 | 0.79 | 23.45 | 0.65 | 20.30 | 24.40 |
| Golden retriever | 171 | 23.78 | 0.67 | 24.00 | 0.80 | 21.50 | 24.50 |
| Great dane | 41 | 23.19 | 0.77 | 23.20 | 1.00 | 21.40 | 24.50 |
| Labrador retriever | 761 | 23.39 | 0.77 | 23.50 | 1.10 | 20.00 | 24.50 |
| Leonberger | 20 | 22.95 | 0.83 | 23.00 | 0.95 | 21.20 | 24.30 |
| Newfoundland | 33 | 23.71 | 0.57 | 23.80 | 0.80 | 22.40 | 24.50 |
| Rottweiler | 128 | 23.12 | 0.83 | 23.10 | 1.00 | 20.40 | 24.50 |
| Saint Bernard | 24 | 22.88 | 0.89 | 22.95 | 0.70 | 19.60 | 24.10 |
|  |  |  |  |  |  |  |  |
| **Herding** |  |  |  |  |  |  |  |
| Bearded collie | 23 | 23.80 | 0.45 | 23.80 | 0.70 | 22.60 | 24.50 |
| Border collie | 146 | 23.36 | 0.83 | 23.40 | 1.28 | 20.20 | 24.50 |
| Old English sheepdog | 27 | 23.20 | 1.01 | 23.40 | 1.65 | 21.20 | 24.40 |
| Rough collie | 15 | 23.27 | 0.48 | 23.30 | 0.40 | 22.00 | 24.20 |
| Shetland sheepdog | 26 | 23.20 | 0.75 | 23.40 | 1.35 | 21.80 | 24.50 |
|  |  |  |  |  |  |  |  |
| **Terrier** |  |  |  |  |  |  |  |
| Airedale | 30 | 23.73 | 0.71 | 24.10 | 1.05 | 22.00 | 24.50 |
| Border terrier | 56 | 23.50 | 0.78 | 23.60 | 1.05 | 21.40 | 24.50 |
| Cairn terrier | 40 | 23.37 | 0.76 | 23.35 | 1.33 | 21.90 | 24.50 |
| Fox terrier | 13 | 23.53 | 0.73 | 23.60 | 0.80 | 21.70 | 24.50 |
| Norfolk terrier | 16 | 22.94 | 1.33 | 23.15 | 1.58 | 19.60 | 24.50 |
| Scottish terrier | 18 | 22.94 | 0.89 | 22.90 | 0.98 | 21.20 | 24.40 |
| West Highland white terrier | 199 | 23.44 | 0.79 | 23.60 | 1.10 | 20.80 | 24.50 |
| Yorkshire terrier | 154 | 23.34 | 0.87 | 23.40 | 1.20 | 20.00 | 24.50 |
|  |  |  |  |  |  |  |  |
| **Scent hound** |  |  |  |  |  |  |  |
| Basset hound | 20 | 23.45 | 0.58 | 23.45 | 0.60 | 22.50 | 24.50 |
| Beagle | 116 | 23.17 | 0.71 | 23.10 | 0.93 | 21.00 | 24.50 |
| Dachshund | 64 | 23.06 | 0.96 | 23.25 | 1.30 | 20.70 | 24.50 |
| Miniature dachshund | 15 | 22.54 | 0.82 | 22.70 | 0.90 | 21.00 | 24.10 |
| Rhodesian ridgeback | 33 | 23.27 | 0.71 | 23.40 | 0.90 | 21.70 | 24.30 |
|  |  |  |  |  |  |  |  |
| **Spaniel/Pointer** |  |  |  |  |  |  |  |
| American cocker spaniel | 12 | 23.40 | 0.61 | 23.35 | 0.75 | 22.40 | 24.30 |
| Cavalier King Charles spaniel | 280 | 23.56 | 0.68 | 23.70 | 0.90 | 21.40 | 24.50 |
| Cocker spaniel | 227 | 23.36 | 0.78 | 23.50 | 1.10 | 20.20 | 24.50 |
| English setter | 19 | 23.27 | 0.82 | 23.30 | 0.85 | 21.90 | 24.50 |
| German shorthaired pointer | 18 | 23.50 | 0.64 | 23.70 | 0.88 | 22.30 | 24.40 |
| Gordon setter | 23 | 23.35 | 0.69 | 23.40 | 0.65 | 21.60 | 24.50 |
| Hungarian vizsla | 33 | 23.33 | 0.79 | 23.30 | 1.20 | 21.40 | 24.50 |
| Irish setter | 44 | 23.69 | 0.57 | 23.80 | 0.85 | 22.30 | 24.50 |
| Italian spinone | 42 | 22.84 | 1.12 | 22.95 | 1.65 | 20.20 | 24.50 |
| Pointer | 13 | 23.55 | 0.60 | 23.60 | 1.00 | 22.70 | 24.40 |
| Springer spaniel | 168 | 23.64 | 0.62 | 23.80 | 0.83 | 21.30 | 24.50 |
| Weimaraner | 103 | 22.63 | 0.90 | 22.60 | 1.30 | 20.40 | 24.30 |
|  |  |  |  |  |  |  |  |
| **Other** |  |  |  |  |  |  |  |
| Bichon frise | 80 | 22.83 | 1.00 | 23.05 | 1.50 | 19.70 | 24.30 |
| Dalmatian | 39 | 23.49 | 0.80 | 23.70 | 1.10 | 21.80 | 24.50 |
| Jack russell terrier | 180 | 23.41 | 0.92 | 23.55 | 1.10 | 19.80 | 24.50 |
| Labradoodle | 16 | 23.51 | 0.51 | 23.60 | 0.35 | 22.40 | 24.40 |
| Lhasa apso | 49 | 22.47 | 0.81 | 22.30 | 1.20 | 20.70 | 24.40 |
| Miniature poodle | 19 | 23.28 | 0.75 | 23.40 | 0.70 | 21.70 | 24.30 |
| Samoyed | 25 | 23.33 | 0.87 | 23.60 | 1.30 | 21.50 | 24.50 |
| Standard poodle | 24 | 23.43 | 0.88 | 23.55 | 1.13 | 21.10 | 24.50 |
| Toy poodle | 15 | 23.45 | 0.85 | 23.80 | 1.10 | 21.80 | 24.50 |
