# Supplementary material for: Breed-Specific Hematological Phenotypes in the Dog: A Natural Resource for the Genetic Dissection of Hematological Parameters in a Mammalian Species
Source: PLoS One. 2013 Nov 25;8(11):e81288. doi: 10.1371/journal.pone.0081288 (PMC3840015; doi:10.1371/journal.pone.0081288)
Supplement: Table S6 — Descriptive statistics – mean cell hemoglobin concentration§. § Unit of measurement: g/dL; SD = standard deviation; IQR = interquartile range; Min. = minimum value recorded; Max. = maximum value recorded. (DOC) [file pone.0081288.s021.doc]

| **Breed** | **N** | **Mean** | **SD** | **Median** | **IQR** | **Min.** | **Max.** |
| --- | --- | --- | --- | --- | --- | --- | --- |
| Mixed breed | 580 | 33.37 | 1.11 | 33.40 | 1.80 | 31.00 | 36.90 |
|  |  |  |  |  |  |  |  |
| **Ancient** |  |  |  |  |  |  |  |
| Akita | 17 | 32.91 | 0.90 | 33.10 | 1.30 | 31.40 | 34.20 |
| Chow chow | 11 | 32.52 | 0.91 | 32.70 | 1.50 | 31.40 | 33.90 |
| Maltese terrier | 23 | 32.97 | 1.03 | 32.90 | 1.30 | 31.40 | 35.40 |
| Shar pei | 42 | 32.88 | 0.90 | 32.80 | 1.43 | 31.30 | 34.60 |
| Siberian husky | 26 | 33.58 | 0.96 | 33.45 | 1.10 | 31.20 | 35.60 |
| Tibetan terrier | 35 | 33.33 | 1.08 | 33.30 | 1.85 | 31.70 | 35.30 |
|  |  |  |  |  |  |  |  |
| **Toy** |  |  |  |  |  |  |  |
| Chihuahua | 18 | 33.24 | 1.08 | 33.25 | 1.43 | 31.20 | 34.70 |
| Pekingese | 17 | 33.64 | 1.03 | 33.60 | 1.60 | 32.10 | 35.60 |
| Pomeranian | 23 | 33.12 | 0.99 | 33.10 | 1.30 | 31.20 | 34.70 |
| Pug | 28 | 34.15 | 1.01 | 34.50 | 1.65 | 32.10 | 35.30 |
| Shih tzu | 92 | 33.26 | 1.17 | 33.20 | 1.83 | 31.00 | 36.00 |
|  |  |  |  |  |  |  |  |
| **Working** |  |  |  |  |  |  |  |
| Dobermann | 77 | 33.09 | 1.06 | 33.10 | 1.80 | 31.20 | 35.40 |
| German shepherd dog | 346 | 33.36 | 1.07 | 33.40 | 1.78 | 31.00 | 36.10 |
| Giant schnauzer | 19 | 33.64 | 0.98 | 33.60 | 1.50 | 31.90 | 35.30 |
| Miniature Schnauzer | 37 | 33.16 | 1.06 | 33.10 | 1.40 | 31.30 | 35.10 |
| Schnauzer | 13 | 33.47 | 1.10 | 33.40 | 1.00 | 31.20 | 35.20 |
|  |  |  |  |  |  |  |  |
| **Sight hound** |  |  |  |  |  |  |  |
| Deerhound | 10 | 33.35 | 0.99 | 33.55 | 1.40 | 31.70 | 34.40 |
| Greyhound | 10 | 33.31 | 1.02 | 33.15 | 1.20 | 31.60 | 34.90 |
| Irish wolfhound | 13 | 33.14 | 1.04 | 33.20 | 1.80 | 31.50 | 34.70 |
|  |  |  |  |  |  |  |  |
| **Mastiff-like** |  |  |  |  |  |  |  |
| Boston terrier | 10 | 33.33 | 1.05 | 33.20 | 1.45 | 31.70 | 35.20 |
| Boxer | 351 | 33.50 | 1.09 | 33.40 | 1.85 | 31.20 | 36.10 |
| Bull mastiff | 46 | 33.12 | 1.17 | 32.95 | 1.60 | 31.00 | 35.90 |
| Bulldog | 16 | 33.09 | 1.01 | 32.75 | 0.67 | 31.90 | 35.20 |
| Dogue de Bordeaux | 31 | 33.17 | 1.11 | 33.00 | 1.45 | 31.10 | 35.50 |
| English bull terrier | 53 | 33.11 | 0.98 | 32.90 | 1.50 | 31.20 | 35.40 |
| Mastiff | 23 | 33.20 | 1.17 | 33.20 | 1.80 | 31.30 | 35.00 |
| Staffordshire bull terrier | 165 | 33.35 | 1.06 | 33.20 | 1.70 | 31.00 | 35.70 |
|  |  |  |  |  |  |  |  |
| **Retriever/other Mastiff-like** |  |  |  |  |  |  |  |
| Bernese mountan dog | 40 | 33.63 | 1.03 | 33.90 | 1.48 | 31.40 | 35.30 |
| Flat-coated retriever | 44 | 33.59 | 1.13 | 33.55 | 1.73 | 31.10 | 35.60 |
| Golden retriever | 171 | 33.15 | 1.08 | 33.00 | 1.75 | 31.20 | 35.70 |
| Great dane | 41 | 33.32 | 1.12 | 33.30 | 1.70 | 31.50 | 36.70 |
| Labrador retriever | 761 | 33.42 | 1.11 | 33.40 | 1.70 | 31.00 | 36.70 |
| Leonberger | 20 | 33.33 | 1.02 | 33.30 | 1.30 | 31.50 | 35.40 |
| Newfoundland | 33 | 33.40 | 1.08 | 33.50 | 1.90 | 31.70 | 35.40 |
| Rottweiler | 128 | 33.40 | 1.08 | 33.50 | 1.63 | 31.00 | 35.70 |
| Saint Bernard | 24 | 33.08 | 0.90 | 32.75 | 1.40 | 32.00 | 34.80 |
|  |  |  |  |  |  |  |  |
| **Herding** |  |  |  |  |  |  |  |
| Bearded collie | 23 | 33.54 | 1.08 | 33.60 | 1.95 | 31.80 | 35.00 |
| Border collie | 146 | 33.13 | 1.09 | 32.90 | 1.78 | 31.10 | 36.20 |
| Old English sheepdog | 27 | 33.08 | 1.07 | 32.90 | 1.85 | 31.20 | 35.00 |
| Rough collie | 15 | 33.19 | 0.80 | 33.00 | 1.30 | 31.70 | 34.30 |
| Shetland sheepdog | 26 | 33.24 | 1.08 | 33.20 | 1.45 | 31.30 | 35.30 |
|  |  |  |  |  |  |  |  |
| **Terrier** |  |  |  |  |  |  |  |
| Airedale | 30 | 32.98 | 0.95 | 32.65 | 1.20 | 31.30 | 34.80 |
| Border terrier | 56 | 33.33 | 0.97 | 33.30 | 1.45 | 31.60 | 35.30 |
| Cairn terrier | 40 | 33.25 | 1.10 | 33.35 | 1.65 | 31.20 | 35.80 |
| Fox terrier | 13 | 33.24 | 1.12 | 33.30 | 1.80 | 31.80 | 35.30 |
| Norfolk terrier | 16 | 33.06 | 1.16 | 32.95 | 1.95 | 31.60 | 35.00 |
| Scottish terrier | 18 | 33.14 | 0.98 | 33.10 | 1.38 | 31.50 | 35.00 |
| West Highland white terrier | 199 | 33.20 | 1.09 | 33.20 | 1.75 | 31.00 | 35.20 |
| Yorkshire terrier | 154 | 33.10 | 1.04 | 33.05 | 1.70 | 31.10 | 35.50 |
|  |  |  |  |  |  |  |  |
| **Scent hound** |  |  |  |  |  |  |  |
| Basset hound | 20 | 33.46 | 0.93 | 33.70 | 1.35 | 31.80 | 35.00 |
| Beagle | 116 | 33.09 | 0.97 | 32.90 | 1.33 | 31.10 | 36.20 |
| Dachshund | 64 | 33.31 | 1.10 | 33.35 | 1.83 | 31.00 | 36.00 |
| Miniature dachshund | 15 | 33.91 | 0.88 | 34.20 | 1.50 | 32.00 | 34.80 |
| Rhodesian ridgeback | 33 | 33.01 | 1.09 | 33.10 | 1.70 | 31.00 | 34.70 |
|  |  |  |  |  |  |  |  |
| **Spaniel/Pointer** |  |  |  |  |  |  |  |
| American cocker spaniel | 12 | 33.23 | 0.70 | 33.05 | 1.13 | 32.20 | 34.20 |
| Cavalier King Charles spaniel | 280 | 33.28 | 1.02 | 33.25 | 1.63 | 31.00 | 35.70 |
| Cocker spaniel | 227 | 33.59 | 1.10 | 33.60 | 1.80 | 31.00 | 36.70 |
| English setter | 19 | 33.21 | 1.03 | 33.60 | 1.60 | 31.30 | 34.60 |
| German shorthaired pointer | 18 | 32.99 | 1.19 | 32.80 | 1.90 | 31.40 | 35.40 |
| Gordon setter | 23 | 33.22 | 0.99 | 33.00 | 1.55 | 31.70 | 34.90 |
| Hungarian vizsla | 33 | 33.21 | 0.97 | 32.90 | 1.50 | 32.00 | 35.40 |
| Irish setter | 44 | 33.12 | 0.96 | 33.10 | 1.50 | 31.50 | 35.20 |
| Italian spinone | 42 | 33.38 | 1.08 | 33.35 | 2.15 | 31.60 | 35.10 |
| Pointer | 13 | 33.12 | 1.26 | 32.90 | 1.40 | 31.10 | 35.40 |
| Springer spaniel | 168 | 33.38 | 1.16 | 33.20 | 2.00 | 31.10 | 35.70 |
| Weimaraner | 103 | 33.13 | 1.14 | 33.00 | 1.95 | 31.30 | 36.00 |
|  |  |  |  |  |  |  |  |
| **Other** |  |  |  |  |  |  |  |
| Bichon frise | 80 | 33.18 | 0.98 | 33.35 | 1.15 | 31.00 | 35.80 |
| Dalmatian | 39 | 32.93 | 0.95 | 32.80 | 1.35 | 31.00 | 34.70 |
| Jack russell terrier | 180 | 33.32 | 1.08 | 33.40 | 1.90 | 31.00 | 35.70 |
| Labradoodle | 16 | 33.78 | 0.92 | 34.05 | 1.75 | 32.60 | 35.30 |
| Lhasa apso | 49 | 33.57 | 1.09 | 33.80 | 1.50 | 31.30 | 35.50 |
| Miniature poodle | 19 | 33.55 | 0.89 | 33.30 | 1.05 | 31.70 | 35.00 |
| Samoyed | 25 | 33.08 | 1.26 | 32.70 | 1.90 | 31.00 | 35.50 |
| Standard poodle | 24 | 33.27 | 1.26 | 33.40 | 2.13 | 31.00 | 35.00 |
| Toy poodle | 15 | 33.30 | 0.88 | 33.10 | 0.95 | 31.90 | 35.30 |
