# Supplementary material for: Breed-Specific Hematological Phenotypes in the Dog: A Natural Resource for the Genetic Dissection of Hematological Parameters in a Mammalian Species
Source: PLoS One. 2013 Nov 25;8(11):e81288. doi: 10.1371/journal.pone.0081288 (PMC3840015; doi:10.1371/journal.pone.0081288)
Supplement: Table S7 — Descriptive statistics – white blood cell concentration§. § Unit of measurement: x 109/L; SD = standard deviation; IQR = interquartile range; Min. = minimum value recorded; Max. = maximum value recorded. (DOC) [file pone.0081288.s022.doc]

| **Breed** | **N** | **Mean** | **SD** | **Median** | **IQR** | **Min.** | **Max.** |
| --- | --- | --- | --- | --- | --- | --- | --- |
| Mixed breed | 580 | 9.45 | 2.17 | 9.12 | 3.01 | 6.00 | 15.90 |
|  |  |  |  |  |  |  |  |
| **Ancient** |  |  |  |  |  |  |  |
| Akita | 17 | 9.75 | 2.55 | 9.05 | 4.20 | 6.23 | 14.90 |
| Chow chow | 11 | 10.26 | 1.97 | 9.46 | 2.82 | 6.98 | 13.00 |
| Maltese terrier | 23 | 9.01 | 2.34 | 8.34 | 2.60 | 6.15 | 14.00 |
| Shar pei | 42 | 10.22 | 2.32 | 10.05 | 3.16 | 6.70 | 15.90 |
| Siberian husky | 26 | 10.49 | 2.08 | 10.45 | 2.12 | 6.77 | 16.00 |
| Tibetan terrier | 35 | 9.92 | 2.30 | 10.10 | 3.60 | 6.00 | 14.60 |
|  |  |  |  |  |  |  |  |
| **Toy** |  |  |  |  |  |  |  |
| Chihuahua | 18 | 10.16 | 2.58 | 10.25 | 4.23 | 6.53 | 14.50 |
| Pekingese | 17 | 9.74 | 2.09 | 9.55 | 2.40 | 6.55 | 15.30 |
| Pomeranian | 23 | 10.09 | 2.63 | 9.23 | 3.88 | 7.02 | 15.80 |
| Pug | 28 | 11.17 | 2.33 | 10.70 | 2.99 | 7.46 | 16.40 |
| Shih tzu | 92 | 10.49 | 2.42 | 10.30 | 3.98 | 6.32 | 15.50 |
|  |  |  |  |  |  |  |  |
| **Working** |  |  |  |  |  |  |  |
| Dobermann | 77 | 9.38 | 1.64 | 9.19 | 2.22 | 6.20 | 13.00 |
| German shepherd dog | 346 | 9.63 | 2.11 | 9.44 | 3.15 | 6.07 | 15.30 |
| Giant schnauzer | 19 | 10.26 | 2.52 | 10.60 | 3.59 | 6.35 | 15.70 |
| Miniature Schnauzer | 37 | 10.36 | 2.52 | 10.13 | 3.09 | 6.18 | 16.80 |
| Schnauzer | 13 | 10.68 | 2.17 | 10.90 | 2.74 | 6.18 | 14.50 |
|  |  |  |  |  |  |  |  |
| **Sight hound** |  |  |  |  |  |  |  |
| Deerhound | 10 | 8.05 | 1.35 | 7.95 | 1.29 | 6.26 | 10.30 |
| Greyhound | 10 | 7.67 | 1.54 | 7.02 | 1.82 | 6.06 | 10.30 |
| Irish wolfhound | 13 | 9.58 | 2.03 | 9.76 | 1.43 | 6.15 | 14.30 |
|  |  |  |  |  |  |  |  |
| **Mastiff-like** |  |  |  |  |  |  |  |
| Boston terrier | 10 | 10.14 | 2.47 | 9.62 | 3.10 | 6.16 | 13.90 |
| Boxer | 351 | 9.55 | 2.27 | 9.18 | 3.28 | 6.04 | 16.20 |
| Bull mastiff | 46 | 10.66 | 2.30 | 10.50 | 3.68 | 6.11 | 15.40 |
| Bulldog | 16 | 11.00 | 2.31 | 10.65 | 2.90 | 7.12 | 15.70 |
| Dogue de Bordeaux | 31 | 11.24 | 2.08 | 11.60 | 3.08 | 6.48 | 14.80 |
| English bull terrier | 53 | 11.30 | 1.98 | 11.40 | 2.50 | 6.59 | 15.30 |
| Mastiff | 23 | 10.64 | 1.98 | 10.50 | 2.58 | 7.24 | 14.10 |
| Staffordshire bull terrier | 165 | 9.54 | 2.10 | 9.40 | 3.01 | 6.01 | 15.10 |
|  |  |  |  |  |  |  |  |
| **Retriever/other Mastiff-like** |  |  |  |  |  |  |  |
| Bernese mountan dog | 40 | 10.40 | 1.72 | 10.45 | 2.85 | 6.89 | 13.10 |
| Flat-coated retriever | 44 | 9.19 | 2.03 | 9.00 | 2.82 | 6.05 | 13.70 |
| Golden retriever | 171 | 9.74 | 2.19 | 9.56 | 3.21 | 6.03 | 16.90 |
| Great dane | 41 | 9.42 | 2.12 | 9.00 | 3.13 | 6.19 | 14.40 |
| Labrador retriever | 761 | 9.36 | 2.07 | 9.09 | 2.96 | 6.00 | 16.30 |
| Leonberger | 20 | 8.63 | 1.81 | 8.31 | 2.13 | 6.10 | 12.70 |
| Newfoundland | 33 | 8.79 | 2.41 | 7.74 | 2.59 | 6.20 | 14.70 |
| Rottweiler | 128 | 10.22 | 2.45 | 10.10 | 3.62 | 6.08 | 16.40 |
| Saint Bernard | 24 | 10.54 | 2.09 | 10.19 | 2.44 | 7.39 | 15.30 |
|  |  |  |  |  |  |  |  |
| **Herding** |  |  |  |  |  |  |  |
| Bearded collie | 23 | 10.26 | 1.66 | 9.38 | 2.91 | 7.38 | 13.00 |
| Border collie | 146 | 9.35 | 2.13 | 8.83 | 2.99 | 6.02 | 16.00 |
| Old English sheepdog | 27 | 9.59 | 1.72 | 9.53 | 2.19 | 6.37 | 12.70 |
| Rough collie | 15 | 8.90 | 1.84 | 9.17 | 2.66 | 6.06 | 11.40 |
| Shetland sheepdog | 26 | 10.90 | 2.62 | 10.35 | 4.49 | 6.70 | 14.90 |
|  |  |  |  |  |  |  |  |
| **Terrier** |  |  |  |  |  |  |  |
| Airedale | 30 | 10.98 | 2.35 | 11.25 | 3.46 | 6.40 | 15.40 |
| Border terrier | 56 | 11.06 | 2.24 | 11.35 | 2.31 | 6.05 | 15.50 |
| Cairn terrier | 40 | 10.92 | 2.37 | 10.90 | 3.28 | 6.00 | 15.50 |
| Fox terrier | 13 | 9.51 | 1.94 | 9.35 | 1.73 | 6.47 | 13.00 |
| Norfolk terrier | 16 | 9.71 | 2.31 | 9.53 | 1.51 | 6.26 | 15.30 |
| Scottish terrier | 18 | 9.17 | 1.85 | 9.02 | 1.99 | 6.51 | 12.60 |
| West Highland white terrier | 199 | 10.29 | 2.23 | 10.10 | 3.22 | 6.18 | 16.80 |
| Yorkshire terrier | 154 | 9.29 | 2.16 | 8.97 | 3.11 | 6.03 | 14.80 |
|  |  |  |  |  |  |  |  |
| **Scent hound** |  |  |  |  |  |  |  |
| Basset hound | 20 | 10.38 | 2.14 | 10.40 | 3.20 | 6.87 | 14.20 |
| Beagle | 116 | 9.68 | 2.17 | 9.21 | 3.09 | 6.22 | 15.00 |
| Dachshund | 64 | 9.57 | 2.07 | 9.27 | 2.79 | 6.04 | 15.20 |
| Miniature dachshund | 15 | 9.67 | 1.83 | 9.86 | 2.63 | 6.14 | 12.00 |
| Rhodesian ridgeback | 33 | 9.30 | 2.27 | 9.52 | 3.55 | 6.02 | 13.20 |
|  |  |  |  |  |  |  |  |
| **Spaniel/Pointer** |  |  |  |  |  |  |  |
| American cocker spaniel | 12 | 12.44 | 1.78 | 12.75 | 2.15 | 8.62 | 14.70 |
| Cavalier King Charles spaniel | 280 | 10.99 | 2.20 | 11.00 | 3.34 | 6.05 | 16.80 |
| Cocker spaniel | 227 | 10.47 | 2.19 | 10.50 | 3.15 | 6.01 | 16.50 |
| English setter | 19 | 8.37 | 2.11 | 7.65 | 3.43 | 6.02 | 12.40 |
| German shorthaired pointer | 18 | 9.52 | 2.18 | 8.53 | 2.92 | 6.66 | 14.30 |
| Gordon setter | 23 | 9.59 | 2.05 | 9.01 | 2.31 | 7.01 | 14.10 |
| Hungarian vizsla | 33 | 9.27 | 1.94 | 9.29 | 2.87 | 6.29 | 13.10 |
| Irish setter | 44 | 10.04 | 2.23 | 9.99 | 3.98 | 6.28 | 13.70 |
| Italian spinone | 42 | 9.33 | 1.66 | 8.86 | 2.17 | 6.34 | 13.00 |
| Pointer | 13 | 9.60 | 1.60 | 9.24 | 0.77 | 7.48 | 13.60 |
| Springer spaniel | 168 | 9.73 | 2.22 | 9.49 | 3.28 | 6.00 | 14.90 |
| Weimaraner | 103 | 9.85 | 2.49 | 9.48 | 3.45 | 6.09 | 15.90 |
|  |  |  |  |  |  |  |  |
| **Other** |  |  |  |  |  |  |  |
| Bichon frise | 80 | 9.91 | 2.35 | 9.61 | 3.53 | 6.08 | 14.70 |
| Dalmatian | 39 | 10.74 | 2.05 | 10.60 | 3.33 | 6.76 | 14.30 |
| Jack russell terrier | 180 | 10.09 | 2.13 | 10.00 | 3.21 | 6.02 | 15.70 |
| Labradoodle | 16 | 10.16 | 2.18 | 9.45 | 3.42 | 6.82 | 13.90 |
| Lhasa apso | 49 | 10.58 | 2.64 | 10.20 | 4.18 | 6.17 | 16.60 |
| Miniature poodle | 19 | 9.40 | 1.76 | 9.11 | 2.08 | 6.28 | 13.30 |
| Samoyed | 25 | 11.04 | 1.97 | 10.90 | 3.22 | 6.91 | 14.10 |
| Standard poodle | 24 | 10.68 | 2.34 | 10.18 | 2.73 | 6.35 | 15.40 |
| Toy poodle | 15 | 10.08 | 1.63 | 9.73 | 2.40 | 7.51 | 12.60 |
