# Supplementary material for: Breed-Specific Hematological Phenotypes in the Dog: A Natural Resource for the Genetic Dissection of Hematological Parameters in a Mammalian Species
Source: PLoS One. 2013 Nov 25;8(11):e81288. doi: 10.1371/journal.pone.0081288 (PMC3840015; doi:10.1371/journal.pone.0081288)
Supplement: Table S8 — Descriptive statistics – neutrophil concentration§. § Unit of measurement: x 109/L; SD = standard deviation; IQR = interquartile range; Min. = minimum value recorded; Max. = maximum value recorded. (DOC) [file pone.0081288.s023.doc]

| **Breed** | **N** | **Mean** | **SD** | **Median** | **IQR** | **Min.** | **Max.** |
| --- | --- | --- | --- | --- | --- | --- | --- |
| Mixed breed | 580 | 6.65 | 1.95 | 6.29 | 2.83 | 3.00 | 11.50 |
|  |  |  |  |  |  |  |  |
| **Ancient** |  |  |  |  |  |  |  |
| Akita | 17 | 6.84 | 2.58 | 6.38 | 4.23 | 3.56 | 11.32 |
| Chow chow | 11 | 7.55 | 1.67 | 7.24 | 2.59 | 4.70 | 9.64 |
| Maltese terrier | 23 | 5.94 | 2.06 | 4.97 | 3.20 | 3.57 | 10.22 |
| Shar pei | 42 | 6.88 | 2.00 | 6.86 | 2.69 | 3.12 | 11.32 |
| Siberian husky | 26 | 6.57 | 1.67 | 6.14 | 1.90 | 4.33 | 10.88 |
| Tibetan terrier | 35 | 7.04 | 2.08 | 6.89 | 3.44 | 3.73 | 10.63 |
|  |  |  |  |  |  |  |  |
| **Toy** |  |  |  |  |  |  |  |
| Chihuahua | 18 | 6.44 | 2.39 | 6.26 | 2.86 | 3.16 | 10.80 |
| Pekingese | 17 | 6.96 | 1.94 | 7.07 | 2.47 | 4.19 | 10.97 |
| Pomeranian | 23 | 6.78 | 2.30 | 6.22 | 3.22 | 3.60 | 11.37 |
| Pug | 28 | 8.08 | 2.10 | 7.95 | 3.78 | 4.74 | 11.27 |
| Shih tzu | 92 | 7.29 | 2.16 | 7.10 | 3.41 | 3.35 | 11.30 |
|  |  |  |  |  |  |  |  |
| **Working** |  |  |  |  |  |  |  |
| Dobermann | 77 | 6.83 | 1.65 | 6.61 | 2.43 | 3.16 | 10.92 |
| German shepherd dog | 346 | 6.71 | 1.87 | 6.54 | 2.62 | 3.15 | 11.50 |
| Giant schnauzer | 19 | 6.97 | 1.98 | 6.72 | 3.29 | 4.06 | 10.08 |
| Miniature Schnauzer | 37 | 7.35 | 1.98 | 7.17 | 3.47 | 4.12 | 11.30 |
| Schnauzer | 13 | 8.00 | 1.98 | 8.86 | 3.02 | 4.20 | 10.30 |
|  |  |  |  |  |  |  |  |
| **Sight hound** |  |  |  |  |  |  |  |
| Deerhound | 10 | 5.56 | 1.41 | 5.24 | 1.48 | 3.99 | 8.48 |
| Greyhound | 10 | 4.70 | 1.25 | 4.29 | 1.58 | 3.45 | 7.00 |
| Irish wolfhound | 13 | 6.80 | 1.93 | 6.86 | 1.24 | 3.55 | 11.44 |
|  |  |  |  |  |  |  |  |
| **Mastiff-like** |  |  |  |  |  |  |  |
| Boston terrier | 10 | 7.58 | 2.10 | 7.58 | 2.72 | 3.82 | 10.43 |
| Boxer | 351 | 6.84 | 1.99 | 6.57 | 2.75 | 3.05 | 11.38 |
| Bull mastiff | 46 | 7.59 | 2.17 | 7.06 | 3.62 | 3.62 | 11.40 |
| Bulldog | 16 | 7.87 | 1.97 | 7.95 | 2.93 | 3.84 | 10.98 |
| Dogue de Bordeaux | 31 | 8.14 | 1.88 | 8.30 | 2.77 | 4.02 | 11.34 |
| English bull terrier | 53 | 8.50 | 1.73 | 8.80 | 2.55 | 4.68 | 11.02 |
| Mastiff | 23 | 7.92 | 1.80 | 7.36 | 2.72 | 5.33 | 11.12 |
| Staffordshire bull terrier | 165 | 6.83 | 1.86 | 6.70 | 2.78 | 3.62 | 11.39 |
|  |  |  |  |  |  |  |  |
| **Retriever/other Mastiff-like** |  |  |  |  |  |  |  |
| Bernese mountan dog | 40 | 7.07 | 1.47 | 6.91 | 2.37 | 4.62 | 10.74 |
| Flat-coated retriever | 44 | 6.43 | 2.04 | 5.95 | 2.71 | 3.54 | 11.23 |
| Golden retriever | 171 | 6.63 | 1.92 | 6.22 | 2.57 | 3.20 | 11.49 |
| Great dane | 41 | 6.44 | 1.97 | 6.01 | 2.98 | 3.59 | 10.66 |
| Labrador retriever | 761 | 6.29 | 1.82 | 6.04 | 2.39 | 3.01 | 11.48 |
| Leonberger | 20 | 6.02 | 1.60 | 5.92 | 2.30 | 3.68 | 9.30 |
| Newfoundland | 33 | 5.91 | 1.78 | 5.14 | 2.19 | 3.78 | 9.93 |
| Rottweiler | 128 | 6.91 | 2.20 | 6.85 | 3.27 | 3.21 | 11.40 |
| Saint Bernard | 24 | 6.98 | 1.96 | 6.84 | 2.59 | 3.55 | 11.10 |
|  |  |  |  |  |  |  |  |
| **Herding** |  |  |  |  |  |  |  |
| Bearded collie | 23 | 7.57 | 1.62 | 7.37 | 2.46 | 4.43 | 10.14 |
| Border collie | 146 | 6.60 | 1.83 | 6.39 | 2.68 | 3.20 | 11.25 |
| Old English sheepdog | 27 | 6.87 | 1.53 | 6.80 | 1.67 | 4.12 | 10.32 |
| Rough collie | 15 | 6.57 | 1.60 | 7.06 | 1.89 | 3.64 | 8.80 |
| Shetland sheepdog | 26 | 8.03 | 2.12 | 7.72 | 3.91 | 4.71 | 11.40 |
|  |  |  |  |  |  |  |  |
| **Terrier** |  |  |  |  |  |  |  |
| Airedale | 30 | 7.72 | 2.09 | 7.62 | 3.32 | 3.61 | 11.47 |
| Border terrier | 56 | 7.80 | 1.73 | 8.06 | 2.14 | 3.93 | 11.17 |
| Cairn terrier | 40 | 7.85 | 2.09 | 7.90 | 3.32 | 3.36 | 11.34 |
| Fox terrier | 13 | 6.89 | 1.41 | 6.72 | 1.19 | 4.40 | 9.62 |
| Norfolk terrier | 16 | 6.60 | 1.88 | 6.05 | 1.87 | 3.94 | 11.17 |
| Scottish terrier | 18 | 6.63 | 1.79 | 6.18 | 1.81 | 4.25 | 10.06 |
| West Highland white terrier | 199 | 7.50 | 1.89 | 7.30 | 2.97 | 3.24 | 11.26 |
| Yorkshire terrier | 154 | 6.51 | 1.95 | 6.32 | 2.80 | 3.09 | 11.40 |
|  |  |  |  |  |  |  |  |
| **Scent hound** |  |  |  |  |  |  |  |
| Basset hound | 20 | 7.27 | 1.94 | 7.29 | 2.91 | 3.02 | 10.37 |
| Beagle | 116 | 6.62 | 1.91 | 6.26 | 2.82 | 3.35 | 11.46 |
| Dachshund | 64 | 6.83 | 1.97 | 6.59 | 2.25 | 3.09 | 11.09 |
| Miniature dachshund | 15 | 6.99 | 1.72 | 6.85 | 2.42 | 4.24 | 9.55 |
| Rhodesian ridgeback | 33 | 6.40 | 1.85 | 6.34 | 2.21 | 3.28 | 10.74 |
|  |  |  |  |  |  |  |  |
| **Spaniel/Pointer** |  |  |  |  |  |  |  |
| American cocker spaniel | 12 | 9.10 | 2.32 | 9.76 | 2.83 | 3.53 | 11.35 |
| Cavalier King Charles spaniel | 280 | 7.71 | 1.88 | 7.64 | 2.82 | 3.21 | 11.42 |
| Cocker spaniel | 227 | 7.63 | 1.88 | 7.55 | 3.07 | 3.51 | 11.22 |
| English setter | 19 | 5.72 | 1.74 | 5.37 | 2.48 | 3.64 | 9.55 |
| German shorthaired pointer | 18 | 6.41 | 2.03 | 5.63 | 3.38 | 3.73 | 10.00 |
| Gordon setter | 23 | 6.41 | 1.91 | 5.90 | 1.76 | 3.98 | 10.20 |
| Hungarian vizsla | 33 | 6.55 | 1.81 | 6.16 | 2.45 | 4.18 | 10.98 |
| Irish setter | 44 | 7.62 | 2.09 | 7.20 | 3.27 | 4.06 | 11.28 |
| Italian spinone | 42 | 6.28 | 1.63 | 6.05 | 2.07 | 3.24 | 10.40 |
| Pointer | 13 | 6.86 | 1.55 | 6.61 | 1.21 | 4.86 | 10.34 |
| Springer spaniel | 168 | 6.99 | 1.94 | 6.85 | 3.05 | 3.27 | 11.43 |
| Weimaraner | 103 | 6.82 | 2.02 | 6.39 | 2.78 | 3.05 | 11.40 |
|  |  |  |  |  |  |  |  |
| **Other** |  |  |  |  |  |  |  |
| Bichon frise | 80 | 6.97 | 2.02 | 6.76 | 2.83 | 3.24 | 11.47 |
| Dalmatian | 39 | 8.02 | 1.83 | 8.04 | 2.78 | 4.19 | 11.40 |
| Jack russell terrier | 180 | 7.24 | 1.87 | 7.10 | 2.65 | 3.00 | 11.42 |
| Labradoodle | 16 | 6.29 | 2.00 | 5.98 | 2.88 | 3.48 | 9.96 |
| Lhasa apso | 49 | 7.18 | 2.20 | 6.64 | 3.89 | 3.27 | 11.17 |
| Miniature poodle | 19 | 6.46 | 1.54 | 5.95 | 2.38 | 4.18 | 10.05 |
| Samoyed | 25 | 7.93 | 1.95 | 7.87 | 1.87 | 3.76 | 10.95 |
| Standard poodle | 24 | 7.62 | 1.87 | 7.58 | 2.12 | 3.24 | 11.15 |
| Toy poodle | 15 | 7.20 | 1.88 | 7.20 | 3.28 | 4.74 | 10.02 |
