# Supplementary material for: Breed-Specific Hematological Phenotypes in the Dog: A Natural Resource for the Genetic Dissection of Hematological Parameters in a Mammalian Species
Source: PLoS One. 2013 Nov 25;8(11):e81288. doi: 10.1371/journal.pone.0081288 (PMC3840015; doi:10.1371/journal.pone.0081288)
Supplement: Table S10 — Descriptive statistics – lymphocyte concentration§. § Unit of measurement: x 109/L; SD = standard deviation; IQR = interquartile range; Min. = minimum value recorded; Max. = maximum value recorded. (DOC) [file pone.0081288.s025.doc]

| **Breed** | **N** | **Mean** | **SD** | **Median** | **IQR** | **Min.** | **Max.** |
| --- | --- | --- | --- | --- | --- | --- | --- |
| Mixed breed | 580 | 1.78 | 0.69 | 1.60 | 0.80 | 1.00 | 4.59 |
|  |  |  |  |  |  |  |  |
| **Ancient** |  |  |  |  |  |  |  |
| Akita | 17 | 1.99 | 0.84 | 1.83 | 1.28 | 1.00 | 3.74 |
| Chow chow | 11 | 1.55 | 0.43 | 1.49 | 0.58 | 1.07 | 2.21 |
| Maltese terrier | 23 | 1.84 | 0.79 | 1.47 | 0.97 | 1.04 | 3.60 |
| Shar pei | 42 | 2.06 | 0.76 | 1.87 | 1.13 | 1.05 | 4.13 |
| Siberian husky | 26 | 2.69 | 0.87 | 2.62 | 1.39 | 1.16 | 4.07 |
| Tibetan terrier | 35 | 1.76 | 0.75 | 1.56 | 0.83 | 1.03 | 4.67 |
|  |  |  |  |  |  |  |  |
| **Toy** |  |  |  |  |  |  |  |
| Chihuahua | 18 | 2.50 | 0.73 | 2.42 | 1.14 | 1.36 | 3.89 |
| Pekingese | 17 | 1.84 | 0.66 | 1.58 | 0.59 | 1.14 | 3.69 |
| Pomeranian | 23 | 2.06 | 0.63 | 1.96 | 0.76 | 1.10 | 3.60 |
| Pug | 28 | 2.01 | 0.94 | 1.72 | 1.28 | 1.02 | 4.34 |
| Shih tzu | 92 | 2.04 | 0.79 | 1.80 | 0.90 | 1.00 | 4.55 |
|  |  |  |  |  |  |  |  |
| **Working** |  |  |  |  |  |  |  |
| Dobermann | 77 | 1.52 | 0.53 | 1.37 | 0.59 | 1.00 | 4.35 |
| German shepherd dog | 346 | 1.62 | 0.57 | 1.44 | 0.63 | 1.00 | 4.34 |
| Giant schnauzer | 19 | 2.11 | 0.82 | 1.96 | 0.93 | 1.07 | 4.40 |
| Miniature Schnauzer | 37 | 1.97 | 0.85 | 1.65 | 0.93 | 1.03 | 4.62 |
| Schnauzer | 13 | 1.77 | 0.86 | 1.61 | 0.55 | 1.05 | 4.14 |
|  |  |  |  |  |  |  |  |
| **Sight hound** |  |  |  |  |  |  |  |
| Deerhound | 10 | 1.61 | 0.50 | 1.45 | 0.76 | 1.01 | 2.59 |
| Greyhound | 10 | 2.24 | 0.67 | 2.07 | 0.46 | 1.52 | 3.84 |
| Irish wolfhound | 13 | 1.80 | 0.65 | 1.60 | 0.59 | 1.08 | 3.25 |
|  |  |  |  |  |  |  |  |
| **Mastiff-like** |  |  |  |  |  |  |  |
| Boston terrier | 10 | 1.50 | 0.49 | 1.33 | 0.44 | 1.06 | 2.50 |
| Boxer | 351 | 1.69 | 0.59 | 1.52 | 0.80 | 1.00 | 4.42 |
| Bull mastiff | 46 | 1.96 | 0.83 | 1.70 | 1.14 | 1.00 | 4.36 |
| Bulldog | 16 | 2.10 | 0.90 | 1.93 | 0.93 | 1.09 | 4.24 |
| Dogue de Bordeaux | 31 | 1.91 | 0.59 | 2.06 | 1.10 | 1.07 | 3.13 |
| English bull terrier | 53 | 1.83 | 0.60 | 1.67 | 0.87 | 1.02 | 3.50 |
| Mastiff | 23 | 1.67 | 0.58 | 1.61 | 0.72 | 1.02 | 2.94 |
| Staffordshire bull terrier | 165 | 1.80 | 0.60 | 1.72 | 0.83 | 1.00 | 4.07 |
|  |  |  |  |  |  |  |  |
| **Retriever/other Mastiff-like** |  |  |  |  |  |  |  |
| Bernese mountan dog | 40 | 2.14 | 0.77 | 1.98 | 1.03 | 1.03 | 4.10 |
| Flat-coated retriever | 44 | 1.65 | 0.54 | 1.46 | 0.79 | 1.01 | 3.45 |
| Golden retriever | 171 | 2.22 | 0.86 | 2.03 | 1.28 | 1.01 | 4.67 |
| Great dane | 41 | 1.77 | 0.85 | 1.55 | 0.57 | 1.04 | 4.75 |
| Labrador retriever | 761 | 1.98 | 0.78 | 1.80 | 1.04 | 1.00 | 4.78 |
| Leonberger | 20 | 1.69 | 0.63 | 1.57 | 0.71 | 1.00 | 3.49 |
| Newfoundland | 33 | 1.81 | 0.67 | 1.73 | 0.66 | 1.01 | 4.41 |
| Rottweiler | 128 | 1.94 | 0.77 | 1.74 | 0.91 | 1.01 | 4.37 |
| Saint Bernard | 24 | 2.21 | 0.90 | 2.18 | 1.62 | 1.10 | 3.90 |
|  |  |  |  |  |  |  |  |
| **Herding** |  |  |  |  |  |  |  |
| Bearded collie | 23 | 1.63 | 0.55 | 1.48 | 0.84 | 1.03 | 3.17 |
| Border collie | 146 | 1.73 | 0.63 | 1.58 | 0.65 | 1.00 | 4.03 |
| Old English sheepdog | 27 | 1.70 | 0.72 | 1.38 | 1.02 | 1.00 | 3.48 |
| Rough collie | 15 | 1.36 | 0.41 | 1.21 | 0.33 | 1.00 | 2.21 |
| Shetland sheepdog | 26 | 1.58 | 0.60 | 1.45 | 0.61 | 1.02 | 3.38 |
|  |  |  |  |  |  |  |  |
| **Terrier** |  |  |  |  |  |  |  |
| Airedale | 30 | 2.16 | 0.79 | 2.00 | 1.10 | 1.07 | 3.89 |
| Border terrier | 56 | 1.97 | 0.81 | 1.81 | 1.12 | 1.00 | 4.65 |
| Cairn terrier | 40 | 2.09 | 0.68 | 1.91 | 0.86 | 1.06 | 3.94 |
| Fox terrier | 13 | 1.69 | 0.60 | 1.57 | 0.23 | 1.05 | 3.20 |
| Norfolk terrier | 16 | 1.88 | 0.58 | 1.71 | 0.54 | 1.00 | 3.20 |
| Scottish terrier | 18 | 1.80 | 0.58 | 1.73 | 0.50 | 1.04 | 3.43 |
| West Highland white terrier | 199 | 1.80 | 0.77 | 1.60 | 0.82 | 1.00 | 4.68 |
| Yorkshire terrier | 154 | 1.88 | 0.72 | 1.71 | 0.81 | 1.00 | 4.66 |
|  |  |  |  |  |  |  |  |
| **Scent hound** |  |  |  |  |  |  |  |
| Basset hound | 20 | 2.14 | 0.85 | 1.89 | 1.15 | 1.07 | 3.55 |
| Beagle | 116 | 2.18 | 0.81 | 2.06 | 1.10 | 1.00 | 4.49 |
| Dachshund | 64 | 1.69 | 0.69 | 1.47 | 0.79 | 1.00 | 3.61 |
| Miniature dachshund | 15 | 1.68 | 0.65 | 1.42 | 0.74 | 1.04 | 3.44 |
| Rhodesian ridgeback | 33 | 1.80 | 0.79 | 1.61 | 0.82 | 1.03 | 4.03 |
|  |  |  |  |  |  |  |  |
| **Spaniel/Pointer** |  |  |  |  |  |  |  |
| American cocker spaniel | 12 | 2.23 | 0.85 | 2.06 | 0.49 | 1.38 | 4.31 |
| Cavalier King Charles spaniel | 280 | 2.20 | 0.79 | 2.07 | 1.13 | 1.00 | 4.79 |
| Cocker spaniel | 227 | 1.84 | 0.66 | 1.70 | 0.94 | 1.00 | 4.06 |
| English setter | 19 | 1.56 | 0.57 | 1.43 | 0.62 | 1.01 | 3.00 |
| German shorthaired pointer | 18 | 1.72 | 0.51 | 1.57 | 0.30 | 1.07 | 3.13 |
| Gordon setter | 23 | 2.08 | 0.59 | 2.10 | 0.64 | 1.07 | 3.44 |
| Hungarian vizsla | 33 | 1.70 | 0.57 | 1.58 | 0.75 | 1.04 | 3.24 |
| Irish setter | 44 | 1.61 | 0.53 | 1.47 | 0.64 | 1.05 | 3.46 |
| Italian spinone | 42 | 1.93 | 0.70 | 1.81 | 0.89 | 1.01 | 3.98 |
| Pointer | 13 | 1.84 | 0.72 | 1.62 | 0.75 | 1.05 | 3.65 |
| Springer spaniel | 168 | 1.71 | 0.59 | 1.56 | 0.63 | 1.00 | 4.64 |
| Weimaraner | 103 | 1.78 | 0.78 | 1.59 | 0.86 | 1.00 | 4.66 |
|  |  |  |  |  |  |  |  |
| **Other** |  |  |  |  |  |  |  |
| Bichon frise | 80 | 1.88 | 0.87 | 1.67 | 0.77 | 1.00 | 4.80 |
| Dalmatian | 39 | 1.79 | 0.76 | 1.55 | 0.72 | 1.01 | 4.13 |
| Jack russell terrier | 180 | 1.79 | 0.69 | 1.62 | 0.96 | 1.00 | 3.78 |
| Labradoodle | 16 | 2.82 | 0.96 | 2.81 | 1.10 | 1.23 | 4.28 |
| Lhasa apso | 49 | 2.41 | 1.01 | 2.22 | 1.23 | 1.01 | 4.67 |
| Miniature poodle | 19 | 1.98 | 0.60 | 2.17 | 0.76 | 1.09 | 3.09 |
| Samoyed | 25 | 1.95 | 0.75 | 1.85 | 0.88 | 1.09 | 3.61 |
| Standard poodle | 24 | 1.91 | 0.84 | 1.70 | 0.59 | 1.07 | 4.16 |
| Toy poodle | 15 | 1.68 | 0.49 | 1.61 | 0.74 | 1.02 | 2.58 |
