# Supplementary material for: Breed-Specific Hematological Phenotypes in the Dog: A Natural Resource for the Genetic Dissection of Hematological Parameters in a Mammalian Species
Source: PLoS One. 2013 Nov 25;8(11):e81288. doi: 10.1371/journal.pone.0081288 (PMC3840015; doi:10.1371/journal.pone.0081288)
Supplement: Table S11 — Descriptive statistics – eosinophil concentration§. § Unit of measurement: x 109/L; SD = standard deviation; IQR = interquartile range; Min. = minimum value recorded; Max. = maximum value recorded. (DOC) [file pone.0081288.s026.doc]

| **Breed** | **N** | **Mean** | **SD** | **Median** | **IQR** | **Min.** | **Max.** |
| --- | --- | --- | --- | --- | --- | --- | --- |
| Mixed breed | 580 | 0.39 | 0.32 | 0.32 | 0.45 | 0.00 | 1.30 |
|  |  |  |  |  |  |  |  |
| **Ancient** |  |  |  |  |  |  |  |
| Akita | 17 | 0.41 | 0.25 | 0.37 | 0.29 | 0.00 | 0.89 |
| Chow chow | 11 | 0.48 | 0.31 | 0.47 | 0.47 | 0.04 | 1.08 |
| Maltese terrier | 23 | 0.45 | 0.34 | 0.44 | 0.48 | 0.00 | 1.16 |
| Shar pei | 42 | 0.54 | 0.30 | 0.50 | 0.38 | 0.00 | 1.23 |
| Siberian husky | 26 | 0.60 | 0.36 | 0.51 | 0.60 | 0.18 | 1.30 |
| Tibetan terrier | 35 | 0.42 | 0.27 | 0.37 | 0.35 | 0.00 | 0.98 |
|  |  |  |  |  |  |  |  |
| **Toy** |  |  |  |  |  |  |  |
| Chihuahua | 18 | 0.46 | 0.37 | 0.39 | 0.58 | 0.00 | 1.16 |
| Pekingese | 17 | 0.28 | 0.28 | 0.17 | 0.21 | 0.00 | 1.00 |
| Pomeranian | 23 | 0.36 | 0.34 | 0.33 | 0.58 | 0.00 | 1.24 |
| Pug | 28 | 0.29 | 0.27 | 0.22 | 0.30 | 0.00 | 1.15 |
| Shih tzu | 92 | 0.38 | 0.28 | 0.31 | 0.30 | 0.00 | 1.29 |
|  |  |  |  |  |  |  |  |
| **Working** |  |  |  |  |  |  |  |
| Dobermann | 77 | 0.49 | 0.33 | 0.50 | 0.49 | 0.00 | 1.28 |
| German shepherd dog | 346 | 0.51 | 0.34 | 0.48 | 0.53 | 0.00 | 1.30 |
| Giant schnauzer | 19 | 0.58 | 0.36 | 0.57 | 0.48 | 0.00 | 1.26 |
| Miniature Schnauzer | 37 | 0.37 | 0.30 | 0.29 | 0.34 | 0.00 | 1.13 |
| Schnauzer | 13 | 0.33 | 0.35 | 0.25 | 0.41 | 0.00 | 1.19 |
|  |  |  |  |  |  |  |  |
| **Sight hound** |  |  |  |  |  |  |  |
| Deerhound | 10 | 0.27 | 0.31 | 0.17 | 0.38 | 0.00 | 0.82 |
| Greyhound | 10 | 0.29 | 0.24 | 0.23 | 0.40 | 0.01 | 0.75 |
| Irish wolfhound | 13 | 0.12 | 0.21 | 0.01 | 0.14 | 0.00 | 0.61 |
|  |  |  |  |  |  |  |  |
| **Mastiff-like** |  |  |  |  |  |  |  |
| Boston terrier | 10 | 0.32 | 0.29 | 0.23 | 0.18 | 0.01 | 0.98 |
| Boxer | 351 | 0.41 | 0.31 | 0.35 | 0.42 | 0.00 | 1.30 |
| Bull mastiff | 46 | 0.35 | 0.27 | 0.33 | 0.33 | 0.00 | 1.18 |
| Bulldog | 16 | 0.28 | 0.28 | 0.17 | 0.51 | 0.00 | 0.79 |
| Dogue de Bordeaux | 31 | 0.40 | 0.31 | 0.38 | 0.49 | 0.00 | 1.16 |
| English bull terrier | 53 | 0.24 | 0.24 | 0.18 | 0.31 | 0.00 | 0.92 |
| Mastiff | 23 | 0.36 | 0.28 | 0.29 | 0.50 | 0.00 | 0.89 |
| Staffordshire bull terrier | 165 | 0.28 | 0.24 | 0.23 | 0.35 | 0.00 | 1.19 |
|  |  |  |  |  |  |  |  |
| **Retriever/other Mastiff-like** |  |  |  |  |  |  |  |
| Bernese mountan dog | 40 | 0.46 | 0.34 | 0.43 | 0.53 | 0.00 | 1.24 |
| Flat-coated retriever | 44 | 0.37 | 0.29 | 0.28 | 0.41 | 0.00 | 1.04 |
| Golden retriever | 171 | 0.29 | 0.27 | 0.23 | 0.39 | 0.00 | 1.26 |
| Great dane | 41 | 0.48 | 0.38 | 0.47 | 0.54 | 0.00 | 1.30 |
| Labrador retriever | 761 | 0.42 | 0.32 | 0.38 | 0.45 | 0.00 | 1.29 |
| Leonberger | 20 | 0.31 | 0.31 | 0.24 | 0.23 | 0.00 | 1.26 |
| Newfoundland | 33 | 0.51 | 0.31 | 0.44 | 0.30 | 0.02 | 1.26 |
| Rottweiler | 128 | 0.58 | 0.35 | 0.57 | 0.54 | 0.00 | 1.30 |
| Saint Bernard | 24 | 0.58 | 0.32 | 0.53 | 0.45 | 0.00 | 1.22 |
|  |  |  |  |  |  |  |  |
| **Herding** |  |  |  |  |  |  |  |
| Bearded collie | 23 | 0.34 | 0.30 | 0.26 | 0.37 | 0.00 | 1.09 |
| Border collie | 146 | 0.35 | 0.31 | 0.30 | 0.45 | 0.00 | 1.29 |
| Old English sheepdog | 27 | 0.41 | 0.29 | 0.33 | 0.42 | 0.00 | 1.12 |
| Rough collie | 15 | 0.28 | 0.17 | 0.29 | 0.22 | 0.00 | 0.58 |
| Shetland sheepdog | 26 | 0.41 | 0.35 | 0.34 | 0.62 | 0.00 | 1.10 |
|  |  |  |  |  |  |  |  |
| **Terrier** |  |  |  |  |  |  |  |
| Airedale | 30 | 0.33 | 0.30 | 0.24 | 0.28 | 0.00 | 1.14 |
| Border terrier | 56 | 0.48 | 0.34 | 0.44 | 0.51 | 0.00 | 1.30 |
| Cairn terrier | 40 | 0.30 | 0.25 | 0.27 | 0.34 | 0.00 | 1.08 |
| Fox terrier | 13 | 0.35 | 0.23 | 0.33 | 0.33 | 0.00 | 0.78 |
| Norfolk terrier | 16 | 0.31 | 0.26 | 0.25 | 0.31 | 0.00 | 0.88 |
| Scottish terrier | 18 | 0.23 | 0.20 | 0.22 | 0.37 | 0.00 | 0.63 |
| West Highland white terrier | 199 | 0.23 | 0.26 | 0.13 | 0.31 | 0.00 | 1.24 |
| Yorkshire terrier | 154 | 0.20 | 0.21 | 0.16 | 0.28 | 0.00 | 1.08 |
|  |  |  |  |  |  |  |  |
| **Scent hound** |  |  |  |  |  |  |  |
| Basset hound | 20 | 0.40 | 0.26 | 0.32 | 0.25 | 0.00 | 0.97 |
| Beagle | 116 | 0.30 | 0.25 | 0.23 | 0.31 | 0.00 | 1.18 |
| Dachshund | 64 | 0.37 | 0.31 | 0.28 | 0.40 | 0.00 | 1.29 |
| Miniature dachshund | 15 | 0.35 | 0.32 | 0.23 | 0.25 | 0.00 | 1.03 |
| Rhodesian ridgeback | 33 | 0.45 | 0.30 | 0.39 | 0.48 | 0.00 | 1.16 |
|  |  |  |  |  |  |  |  |
| **Spaniel/Pointer** |  |  |  |  |  |  |  |
| American cocker spaniel | 12 | 0.32 | 0.29 | 0.35 | 0.53 | 0.00 | 0.73 |
| Cavalier King Charles spaniel | 280 | 0.42 | 0.30 | 0.36 | 0.41 | 0.00 | 1.29 |
| Cocker spaniel | 227 | 0.32 | 0.29 | 0.26 | 0.45 | 0.00 | 1.30 |
| English setter | 19 | 0.36 | 0.32 | 0.23 | 0.49 | 0.00 | 0.96 |
| German shorthaired pointer | 18 | 0.58 | 0.33 | 0.52 | 0.52 | 0.13 | 1.17 |
| Gordon setter | 23 | 0.51 | 0.31 | 0.48 | 0.46 | 0.00 | 1.20 |
| Hungarian vizsla | 33 | 0.37 | 0.31 | 0.23 | 0.43 | 0.00 | 1.03 |
| Irish setter | 44 | 0.21 | 0.23 | 0.12 | 0.36 | 0.00 | 0.74 |
| Italian spinone | 42 | 0.51 | 0.32 | 0.52 | 0.45 | 0.00 | 1.19 |
| Pointer | 13 | 0.26 | 0.22 | 0.23 | 0.30 | 0.00 | 0.63 |
| Springer spaniel | 168 | 0.30 | 0.30 | 0.23 | 0.37 | 0.00 | 1.30 |
| Weimaraner | 103 | 0.48 | 0.34 | 0.44 | 0.56 | 0.00 | 1.28 |
|  |  |  |  |  |  |  |  |
| **Other** |  |  |  |  |  |  |  |
| Bichon frise | 80 | 0.33 | 0.30 | 0.24 | 0.35 | 0.00 | 1.30 |
| Dalmatian | 39 | 0.39 | 0.27 | 0.38 | 0.35 | 0.00 | 0.97 |
| Jack russell terrier | 180 | 0.33 | 0.28 | 0.26 | 0.38 | 0.00 | 1.20 |
| Labradoodle | 16 | 0.53 | 0.35 | 0.53 | 0.42 | 0.00 | 1.25 |
| Lhasa apso | 49 | 0.38 | 0.30 | 0.30 | 0.46 | 0.00 | 1.11 |
| Miniature poodle | 19 | 0.22 | 0.25 | 0.17 | 0.26 | 0.00 | 0.78 |
| Samoyed | 25 | 0.39 | 0.39 | 0.26 | 0.56 | 0.00 | 1.20 |
| Standard poodle | 24 | 0.40 | 0.35 | 0.27 | 0.43 | 0.00 | 1.14 |
| Toy poodle | 15 | 0.17 | 0.20 | 0.08 | 0.25 | 0.00 | 0.68 |
