# Supplementary material for: Breed-Specific Hematological Phenotypes in the Dog: A Natural Resource for the Genetic Dissection of Hematological Parameters in a Mammalian Species
Source: PLoS One. 2013 Nov 25;8(11):e81288. doi: 10.1371/journal.pone.0081288 (PMC3840015; doi:10.1371/journal.pone.0081288)
Supplement: Table S12 — Descriptive statistics – platelet concentration§. § Unit of measurement: x 109/L; SD = standard deviation; IQR = interquartile range; Min. = minimum value recorded; Max. = maximum value recorded. (DOC) [file pone.0081288.s027.doc]

| **Breed** | **N** | **Mean** | **SD** | **Median** | **IQR** | **Min.** | **Max.** |
| --- | --- | --- | --- | --- | --- | --- | --- |
| Mixed breed | 580 | 328.87 | 102.26 | 311.00 | 135.00 | 150.00 | 630.00 |
|  |  |  |  |  |  |  |  |
| **Ancient** |  |  |  |  |  |  |  |
| Akita | 7 | 239.00 | 56.33 | 236.00 | 40.00 | 170.00 | 349.00 |
| Chow chow | 11 | 299.09 | 133.56 | 246.00 | 197.50 | 170.00 | 534.00 |
| Maltese terrier | 22 | 361.45 | 132.08 | 334.00 | 130.00 | 193.00 | 646.00 |
| Shar pei | 40 | 315.20 | 96.85 | 294.50 | 122.25 | 175.00 | 600.00 |
| Siberian husky | 25 | 263.44 | 72.29 | 253.00 | 104.00 | 153.00 | 410.00 |
| Tibetan terrier | 33 | 355.15 | 125.44 | 336.00 | 169.00 | 151.00 | 677.00 |
|  |  |  |  |  |  |  |  |
| **Toy** |  |  |  |  |  |  |  |
| Chihuahua | 17 | 406.53 | 99.98 | 406.00 | 108.00 | 186.00 | 599.00 |
| Pekingese | 17 | 428.00 | 71.36 | 432.00 | 86.00 | 339.00 | 599.00 |
| Pomeranian | 23 | 446.91 | 117.55 | 442.00 | 154.00 | 179.00 | 656.00 |
| Pug | 27 | 419.96 | 110.19 | 432.00 | 144.00 | 223.00 | 636.00 |
| Shih tzu | 87 | 358.39 | 98.19 | 355.00 | 141.00 | 167.00 | 621.00 |
|  |  |  |  |  |  |  |  |
| **Working** |  |  |  |  |  |  |  |
| Dobermann | 73 | 298.59 | 101.91 | 273.00 | 116.00 | 158.00 | 603.00 |
| German shepherd dog | 316 | 265.58 | 79.31 | 250.00 | 101.50 | 151.00 | 641.00 |
| Giant schnauzer | 18 | 330.78 | 87.52 | 318.00 | 146.00 | 185.00 | 478.00 |
| Miniature Schnauzer | 33 | 371.91 | 129.22 | 374.00 | 143.00 | 177.00 | 628.00 |
| Schnauzer | 10 | 326.70 | 142.68 | 313.00 | 250.75 | 156.00 | 526.00 |
|  |  |  |  |  |  |  |  |
| **Sight hound** |  |  |  |  |  |  |  |
| Deerhound | 9 | 208.56 | 72.52 | 185.00 | 45.00 | 156.00 | 383.00 |
| Greyhound | 10 | 221.90 | 33.95 | 224.00 | 32.00 | 184.00 | 302.00 |
| Irish wolfhound | 11 | 253.36 | 48.40 | 266.00 | 57.50 | 163.00 | 324.00 |
|  |  |  |  |  |  |  |  |
| **Mastiff-like** |  |  |  |  |  |  |  |
| Boston terrier | 9 | 491.33 | 118.66 | 477.00 | 185.00 | 319.00 | 659.00 |
| Boxer | 334 | 319.20 | 81.32 | 315.00 | 110.50 | 150.00 | 545.00 |
| Bull mastiff | 40 | 287.20 | 65.45 | 285.50 | 92.75 | 158.00 | 420.00 |
| Bulldog | 14 | 387.50 | 112.96 | 387.50 | 92.75 | 186.00 | 582.00 |
| Dogue de Bordeaux | 30 | 262.43 | 67.08 | 256.50 | 96.00 | 152.00 | 388.00 |
| English bull terrier | 51 | 315.12 | 84.53 | 303.00 | 129.50 | 193.00 | 625.00 |
| Mastiff | 21 | 261.24 | 55.16 | 270.00 | 71.00 | 176.00 | 375.00 |
| Staffordshire bull terrier | 156 | 354.37 | 106.78 | 339.50 | 148.50 | 153.00 | 683.00 |
|  |  |  |  |  |  |  |  |
| **Retriever/other Mastiff-like** |  |  |  |  |  |  |  |
| Bernese mountan dog | 39 | 294.41 | 65.45 | 287.00 | 78.00 | 198.00 | 461.00 |
| Flat-coated retriever | 43 | 321.44 | 106.93 | 294.00 | 107.50 | 172.00 | 665.00 |
| Golden retriever | 164 | 302.90 | 87.13 | 298.00 | 106.50 | 151.00 | 658.00 |
| Great dane | 31 | 245.55 | 64.93 | 240.00 | 71.50 | 153.00 | 387.00 |
| Labrador retriever | 682 | 280.24 | 81.05 | 266.00 | 100.75 | 150.00 | 665.00 |
| Leonberger | 18 | 291.33 | 53.04 | 287.50 | 60.00 | 229.00 | 401.00 |
| Newfoundland | 27 | 246.52 | 83.45 | 240.00 | 79.50 | 163.00 | 574.00 |
| Rottweiler | 119 | 377.58 | 104.39 | 374.00 | 129.50 | 188.00 | 681.00 |
| Saint Bernard | 21 | 269.95 | 73.75 | 254.00 | 101.00 | 161.00 | 413.00 |
|  |  |  |  |  |  |  |  |
| **Herding** |  |  |  |  |  |  |  |
| Bearded collie | 22 | 243.45 | 58.41 | 237.00 | 42.00 | 154.00 | 384.00 |
| Border collie | 133 | 327.74 | 101.71 | 298.00 | 121.00 | 168.00 | 663.00 |
| Old English sheepdog | 23 | 307.83 | 120.98 | 294.00 | 112.00 | 156.00 | 684.00 |
| Rough collie | 13 | 269.46 | 81.36 | 254.00 | 102.00 | 169.00 | 438.00 |
| Shetland sheepdog | 24 | 411.50 | 137.79 | 388.50 | 216.00 | 151.00 | 658.00 |
|  |  |  |  |  |  |  |  |
| **Terrier** |  |  |  |  |  |  |  |
| Airedale | 30 | 376.23 | 108.17 | 376.00 | 174.00 | 169.00 | 601.00 |
| Border terrier | 52 | 390.33 | 113.96 | 372.00 | 141.75 | 188.00 | 688.00 |
| Cairn terrier | 34 | 345.38 | 105.57 | 323.00 | 97.00 | 172.00 | 685.00 |
| Fox terrier | 13 | 287.31 | 57.44 | 275.00 | 82.00 | 197.00 | 378.00 |
| Norfolk terrier | 12 | 369.25 | 80.88 | 404.00 | 110.50 | 188.00 | 445.00 |
| Scottish terrier | 18 | 374.28 | 137.61 | 344.50 | 143.25 | 193.00 | 693.00 |
| West Highland white terrier | 182 | 412.79 | 121.40 | 397.50 | 175.50 | 167.00 | 697.00 |
| Yorkshire terrier | 145 | 359.05 | 126.23 | 331.00 | 169.00 | 150.00 | 671.00 |
|  |  |  |  |  |  |  |  |
| **Scent hound** |  |  |  |  |  |  |  |
| Basset hound | 20 | 288.85 | 88.13 | 269.00 | 56.25 | 198.00 | 524.00 |
| Beagle | 110 | 295.52 | 84.47 | 278.50 | 113.75 | 177.00 | 554.00 |
| Dachshund | 59 | 370.80 | 115.17 | 345.00 | 167.00 | 153.00 | 673.00 |
| Miniature dachshund | 14 | 394.64 | 145.57 | 421.50 | 173.50 | 162.00 | 651.00 |
| Rhodesian ridgeback | 28 | 250.21 | 75.10 | 246.50 | 91.00 | 152.00 | 483.00 |
|  |  |  |  |  |  |  |  |
| **Spaniel/Pointer** |  |  |  |  |  |  |  |
| American cocker spaniel | 10 | 410.80 | 138.30 | 383.50 | 176.50 | 248.00 | 648.00 |
| Cavalier King Charles spaniel | 146 | 302.88 | 116.23 | 283.50 | 154.25 | 151.00 | 689.00 |
| Cocker spaniel | 210 | 324.25 | 99.41 | 311.00 | 129.75 | 151.00 | 680.00 |
| English setter | 19 | 291.58 | 70.89 | 270.00 | 88.50 | 183.00 | 473.00 |
| German shorthaired pointer | 17 | 303.06 | 65.63 | 318.00 | 91.00 | 173.00 | 390.00 |
| Gordon setter | 18 | 304.50 | 61.54 | 310.50 | 64.75 | 203.00 | 478.00 |
| Hungarian vizsla | 28 | 272.04 | 69.41 | 265.50 | 93.25 | 156.00 | 399.00 |
| Irish setter | 40 | 316.98 | 85.90 | 307.50 | 92.25 | 163.00 | 556.00 |
| Italian spinone | 40 | 294.90 | 105.77 | 274.00 | 115.50 | 157.00 | 570.00 |
| Pointer | 12 | 288.08 | 59.02 | 281.50 | 85.25 | 209.00 | 387.00 |
| Springer spaniel | 151 | 323.10 | 92.13 | 314.00 | 113.00 | 153.00 | 673.00 |
| Weimaraner | 97 | 298.72 | 71.69 | 296.00 | 99.00 | 159.00 | 498.00 |
|  |  |  |  |  |  |  |  |
| **Other** |  |  |  |  |  |  |  |
| Bichon frise | 76 | 400.47 | 125.33 | 383.00 | 183.75 | 213.00 | 689.00 |
| Dalmatian | 39 | 329.41 | 93.78 | 320.00 | 132.00 | 153.00 | 534.00 |
| Jack russell terrier | 162 | 347.26 | 110.64 | 332.00 | 136.50 | 159.00 | 679.00 |
| Labradoodle | 15 | 249.00 | 71.47 | 260.00 | 116.00 | 150.00 | 374.00 |
| Lhasa apso | 46 | 340.28 | 109.11 | 332.00 | 138.50 | 152.00 | 666.00 |
| Miniature poodle | 16 | 337.25 | 102.47 | 341.50 | 185.75 | 183.00 | 485.00 |
| Samoyed | 23 | 275.70 | 74.62 | 275.00 | 113.00 | 152.00 | 403.00 |
| Standard poodle | 20 | 288.25 | 62.93 | 291.00 | 79.75 | 175.00 | 396.00 |
| Toy poodle | 13 | 344.54 | 83.72 | 349.00 | 130.00 | 224.00 | 462.00 |
