# Supplementary material for: Breed-Specific Hematological Phenotypes in the Dog: A Natural Resource for the Genetic Dissection of Hematological Parameters in a Mammalian Species
Source: PLoS One. 2013 Nov 25;8(11):e81288. doi: 10.1371/journal.pone.0081288 (PMC3840015; doi:10.1371/journal.pone.0081288)
Supplement: Table S13 — Statistical analysis of the effects of age, sex and neutering status. This table shows the results of a linear mixed effects model to assess the effect of age, sex and neutering status, and all two-way and three-way interactions on each hematological parameter, taking breed as a random effect. Red cell parameters Abbreviations: RBC=red blood cell concentration; MCV=mean corpuscular volume; Hct=hematocrit; Hb=hemoglobin; MCH=mean corpuscular hemoglobin; MCHC=mean corpuscular hemoglobin concentration. White cell and platelet concentrations Abbreviations: Concentrations of WBC=white blood cells, Neut=neutrophils, Mono=monocytes, Lymph=lymphocytes, Eosin=eosinophils and PLT=platelets (DOC) [file pone.0081288.s028.doc]

| Effect | **RBC** | **MCV** | **Hct** | **Hb** | **MCH** | **MCHC** |
| --- | --- | --- | --- | --- | --- | --- |
| **Age** | 6.03x10^-17^ | 0.002 | 6.14x10^-24^ | 6.82x10^-30^ | 1.66x10^-8^ | 0.244 |
| **Sex** | 0.034 | 0.288 | 0.122 | 0.134 | 0.153 | 0.748 |
| **Neutering status** | 0.427 | 0.409 | 0.225 | 0.043 | 6.85x10^-3^ | 7.33x10^-13^ |
| **Age*sex** | 0.081 | 0.021 | 0.611 | 0.340 | 0.133 | 0.852 |
| **Age*neutering status** | 0.031 | 0.895 | 0.041 | 8.21x10^-3^ | 0.472 | 0.560 |
| **Sex*neutering status** | 0.346 | 0.474 | 0.120 | 0.219 | 0.689 | 0.696 |
| **Age*sex*neutering status** | 0.145 | 0.491 | 0.336 | 0.476 | 0.112 | 0.694 |

| Effect | **WBC** | **Neut** | **Mono** | **Lymph** | **Eosin** | **PLT** |
| --- | --- | --- | --- | --- | --- | --- |
| **Age** | 6.76x10^-14^ | 0.055 | 2.30x10^-7^ | 6.90x10^-115^ | 9.03x10^-10^ | 1.50x10^-111^ |
| **Sex** | 2.94x10^-5^ | 6.20x10^-4^ | 3.67x10^-5^ | 0.621 | 9.87x10^-4^ | 1.01x10^-6^ |
| **Neutering status** | 6.60x10^-7^ | 3.37x10^-7^ | 1.48x10^-6^ | 0.505 | 0.178 | 7.33x10^-13^ |
| **Age*sex** | 0.817 | 0.839 | 0.871 | 0.534 | 0.972 | 0.931 |
| **Age*neutering status** | 0.023 | 0.140 | 0.526 | 1.62x10^-5^ | 0.990 | 0.499 |
| **Sex*neutering status** | 0.166 | 0.023 | 0.247 | 0.053 | 0.253 | 0.013 |
| **Age*sex*neutering status** | 0.646 | 0.876 | 0.727 | 0.351 | 0.160 | 0.191 |
