# Supplementary material for: Breed-Specific Hematological Phenotypes in the Dog: A Natural Resource for the Genetic Dissection of Hematological Parameters in a Mammalian Species
Source: PLoS One. 2013 Nov 25;8(11):e81288. doi: 10.1371/journal.pone.0081288 (PMC3840015; doi:10.1371/journal.pone.0081288)
Supplement: Table S14 — Principal component analysis – Eigenvalues of the correlation matrix. (DOC) [file pone.0081288.s029.doc]

| **Principal**  **component** | **Eigenvalue** | **Associated variation (%)** | **Cumulative variation (%)** |
| --- | --- | --- | --- |
| 1 | 3.4577 | 28.81 | 28.81 |
| 2 | 2.3046 | 19.2 | 48.02 |
| 3 | 2.0112 | 16.76 | 64.78 |
| 4 | 1.0887 | 9.07 | 73.85 |
| 5 | 1.0485 | 8.74 | 82.59 |
| 6 | 0.9164 | 7.64 | 90.23 |
| 7 | 0.7420 | 6.18 | 96.41 |
| 8 | 0.4142 | 3.45 | 99.86 |
| 9 | 0.0129 | 0.11 | 99.97 |
| 10 | 0.0020 | 0.02 | 99.99 |
| 11 | 0.0015 | 0.01 | 100.00 |
