# Supplementary material for: Breed-Specific Hematological Phenotypes in the Dog: A Natural Resource for the Genetic Dissection of Hematological Parameters in a Mammalian Species
Source: PLoS One. 2013 Nov 25;8(11):e81288. doi: 10.1371/journal.pone.0081288 (PMC3840015; doi:10.1371/journal.pone.0081288)
Supplement: Table S15 — Statistical analysis of pairwise comparisons of hematological parameters between the pure breed and mixed breed dogs. The results of two-sample Kolmogorov-Smirnov tests to compare the distributions of residuals for the pure breed versus mixed breed dogs are shown as the -log10(p value). (Residuals were defined as the observed values minus the estimated fixed effects of age, sex and neutering status.) A Bonferroni correction to account for multiple comparisons was applied, yielding a threshold for significance of 10-4 (i.e. 4 as stated in the table). Abbreviations: RBC=red blood cell concentration; MCV=mean corpuscular volume; Hct=hematocrit; Hb=hemoglobin; MCH=mean corpuscular hemoglobin; MCHC=mean corpuscular hemoglobin concentration; concentrations of WBC=white blood cell, Neut=neutrophils, Mono=monocytes, Lymph=lymphocytes, Eosin=eosinophils and PLT=platelets. (DOC) [file pone.0081288.s030.doc]

| Breed | RBC | MCV | Hct | Hb | MCH | MCHC | WBC | Neut | Mono | Lymph | Eosin | PLT |
| --- | --- | --- | --- | --- | --- | --- | --- | --- | --- | --- | --- | --- |
| **Ancient** |  |  |  |  |  |  |  |  |  |  |  |  |
| Akita | 1.24990 | 9.60143 | 0.77413 | 1.37080 | 10.31244 | 0.87254 | 0.12026 | 0.18487 | 0.65455 | 0.51745 | 0.20859 | 1.43994 |
| Chow chow | 1.36216 | 3.76638 | 0.06400 | 1.12381 | 7.03441 | 0.99949 | 0.60837 | 0.78995 | 0.09730 | 0.11149 | 0.39915 | 0.47576 |
| Maltese terrier | 0.17932 | 1.37861 | 0.27912 | 0.35412 | 0.05859 | 0.69666 | 0.29615 | 2.12399 | 1.14623 | 0.36908 | 0.29376 | 0.75043 |
| Shar pei | 1.85448 | 10.60019 | 0.49322 | 0.99570 | 15.65758 | 1.70802 | 0.57372 | 0.49912 | 1.45965 | 0.84864 | 2.47590 | 0.25964 |
| Siberian husky | 2.78489 | 0.04977 | 2.86337 | 2.76759 | 0.20990 | 0.81999 | 0.80959 | 0.08746 | 0.22741 | 3.99371 | 1.51242 | 0.64827 |
| Tibetan terrier | 1.57301 | 6.64240 | 0.08810 | 0.11705 | 5.77475 | 0.06620 | 0.56451 | 0.81791 | 0.41675 | 0.03647 | 0.64436 | 0.56393 |
|  |  |  |  |  |  |  |  |  |  |  |  |  |
| **Toy** |  |  |  |  |  |  |  |  |  |  |  |  |
| Chihuahua | 0.49546 | 1.08476 | 0.57651 | 0.42889 | 0.10955 | 0.02552 | 0.11966 | 0.09786 | 0.51529 | 2.32919 | 0.07648 | 4.16238 |
| Pekingese | 0.87670 | 1.19491 | 0.84116 | 0.99449 | 0.62048 | 0.16327 | 0.45305 | 0.14204 | 0.07979 | 0.67032 | 0.97794 | 4.95699 |
| Pomeranian | 0.06967 | 2.14244 | 0.61789 | 0.79594 | 3.01184 | 0.54614 | 0.27353 | 0.00177 | 2.29367 | 0.94362 | 0.50888 | 6.44591 |
| Pug | 1.99420 | 0.60735 | 2.04117 | 1.24778 | 0.97197 | 2.84895 | 1.57985 | 1.59615 | 1.85266 | 0.71150 | 1.05628 | 5.27737 |
| Shih tzu | 0.34322 | 0.97296 | 0.93948 | 1.65059 | 2.17976 | 0.39845 | 2.45571 | 1.27885 | 3.26660 | 4.92195 | 0.52653 | 3.21941 |
|  |  |  |  |  |  |  |  |  |  |  |  |  |
| **Working** |  |  |  |  |  |  |  |  |  |  |  |  |
| Dobermann | 0.20314 | 0.13679 | 0.04879 | 0.36495 | 1.47423 | 0.53466 | 0.30741 | 0.66435 | 0.65998 | 3.68780 | 1.41783 | 0.40396 |
| German shepherd dog | 1.70953 | 2.03630 | 1.07761 | 0.94946 | 3.64781 | 0.07580 | 0.54824 | 0.07873 | 12.05259 | 3.79147 | 6.65095 | 10.84277 |
| Giant schnauzer | 0.23159 | 2.01460 | 0.21739 | 0.21739 | 1.80117 | 0.17365 | 0.32573 | 0.09734 | 0.08910 | 1.03049 | 1.06164 | 0.61220 |
| Miniature Schnauzer | 0.32759 | 1.56677 | 0.14489 | 0.34640 | 1.43748 | 0.19276 | 1.05529 | 1.33306 | 0.02679 | 0.00480 | 0.36680 | 2.64729 |
| Schnauzer | 0.70394 | 0.03977 | 1.29019 | 0.78851 | 0.23745 | 0.15129 | 0.66989 | 1.42526 | 0.17591 | 0.75933 | 0.17851 | 0.12484 |
| Sight hound |  |  |  |  |  |  |  |  |  |  |  |  |
| Deerhound | 1.46803 | 2.31441 | 1.13676 | 0.67458 | 1.80430 | 0.21062 | 0.98402 | 0.49595 | 0.48711 | 0.71466 | 0.98402 | 1.67610 |
| Greyhound | 3.65927 | 0.41014 | 3.40289 | 3.11771 | 0.15639 | 0.23688 | 1.52204 | 2.28192 | 1.61852 | 1.81894 | 0.08274 | 3.30342 |
| Irish wolfhound | 0.27942 | 0.13937 | 0.48341 | 0.68204 | 1.28685 | 0.01096 | 0.20544 | 0.14125 | 1.28130 | 0.98811 | 3.69245 | 0.86552 |
|  |  |  |  |  |  |  |  |  |  |  |  |  |
| **Mastiff-like** |  |  |  |  |  |  |  |  |  |  |  |  |
| Boston terrier | 0.25738 | 0.15639 | 0.07466 | 0.06337 | 0.09124 | 0.00532 | 0.07865 | 0.61605 | 0.38556 | 1.04174 | 0.41845 | 3.56477 |
| Boxer | 0.28675 | 0.85740 | 0.82962 | 0.64908 | 0.46825 | 0.56756 | 0.19337 | 0.38292 | 0.28510 | 3.19907 | 0.07550 | 2.69735 |
| Bull mastiff | 0.78251 | 0.43232 | 0.25064 | 1.62778 | 2.02239 | 0.57254 | 2.26623 | 1.02425 | 1.67237 | 0.22267 | 0.44461 | 0.48317 |
| Bulldog | 0.39775 | 3.16216 | 0.18328 | 0.05385 | 3.12685 | 1.13889 | 1.09735 | 1.14651 | 0.80701 | 0.07550 | 0.66851 | 2.52394 |
| Dogue De Bordeaux | 0.35551 | 0.16653 | 0.28071 | 0.26328 | 0.02857 | 0.33041 | 2.83606 | 2.96485 | 0.56417 | 0.06281 | 0.06221 | 0.86326 |
| English bull terrier | 0.31711 | 2.51880 | 0.06115 | 0.02317 | 0.54371 | 0.84203 | 6.57251 | 7.55707 | 1.75273 | 0.32920 | 2.75338 | 0.50134 |
| Mastiff | 0.18759 | 0.19765 | 0.04596 | 0.16968 | 0.38903 | 0.14679 | 1.68994 | 2.39198 | 0.67713 | 0.34902 | 0.10842 | 1.13520 |
| Staffordshire bull terrier | 1.71062 | 0.64732 | 0.51175 | 0.63028 | 0.52755 | 0.19743 | 0.40511 | 0.49223 | 0.21339 | 0.48257 | 3.00167 | 4.54579 |
|  |  |  |  |  |  |  |  |  |  |  |  |  |
| **Retriever/other Mastiff-like** | | |  |  |  |  |  |  |  |  |  |  |
| Bernese mountan dog | 1.44680 | 0.05727 | 1.12528 | 0.59956 | 0.23940 | 0.72435 | 2.28177 | 1.28195 | 1.97549 | 1.91655 | 0.65609 | 0.18438 |
| Flat-coated retriever | 3.81225 | 0.91217 | 3.21078 | 3.62867 | 0.35675 | 0.30292 | 0.10003 | 0.13467 | 1.16028 | 0.80763 | 0.22995 | 0.33604 |
| Golden retriever | 13.36464 | 8.19760 | 5.56535 | 10.15019 | 7.52759 | 1.66459 | 0.74619 | 0.03452 | 0.14108 | 7.79082 | 2.63412 | 0.62823 |
| Great dane | 1.46853 | 1.38816 | 0.54110 | 1.44987 | 2.03346 | 0.12190 | 0.11617 | 0.11711 | 1.22105 | 0.88381 | 0.81164 | 2.45006 |
| Labrador retriever | 7.89204 | 0.28213 | 9.45569 | 9.01096 | 1.14974 | 0.65320 | 0.72831 | 2.99160 | 0.61131 | 3.19154 | 1.33036 | 6.79126 |
| Leonberger | 3.28435 | 1.15867 | 3.58471 | 3.52920 | 1.96374 | 0.16106 | 0.69187 | 0.51834 | 0.32026 | 0.38619 | 0.70595 | 0.34975 |
| Newfoundland | 4.34148 | 0.54001 | 3.48890 | 3.06700 | 0.67177 | 0.27720 | 2.22297 | 2.46088 | 0.77218 | 0.04983 | 1.81000 | 2.89707 |
| Rottweiler | 6.66804 | 2.45573 | 9.60842 | 9.86254 | 5.70225 | 0.20174 | 1.69850 | 0.38815 | 5.02695 | 0.78770 | 7.15405 | 10.37892 |
| Saint Bernard | 1.56323 | 0.66686 | 2.07128 | 2.71592 | 3.89356 | 0.58321 | 1.43221 | 0.30688 | 1.15332 | 1.75395 | 3.25625 | 1.46100 |
|  |  |  |  |  |  |  |  |  |  |  |  |  |
| **Herding** |  |  |  |  |  |  |  |  |  |  |  |  |
| Bearded collie | 0.12609 | 0.68687 | 0.97682 | 0.61039 | 0.99022 | 0.20173 | 2.63110 | 2.64575 | 0.23086 | 0.55274 | 0.15584 | 3.63157 |
| Border collie | 3.60537 | 1.11807 | 1.71656 | 3.68566 | 1.18047 | 1.30629 | 0.50387 | 0.01832 | 0.02662 | 1.22242 | 1.15684 | 0.64383 |
| Old English sheepdog | 0.54687 | 0.54027 | 0.73706 | 0.77802 | 0.39320 | 0.29727 | 0.17357 | 0.66114 | 0.53097 | 0.61451 | 0.21706 | 0.72987 |
| Rough collie | 0.64607 | 0.49814 | 0.13588 | 0.76903 | 1.53735 | 0.47309 | 0.45548 | 0.10546 | 0.18188 | 2.52648 | 0.98186 | 0.43083 |
| Shetland sheepdog | 0.67763 | 0.25474 | 0.61199 | 0.69841 | 0.46033 | 0.04749 | 1.02566 | 1.33435 | 3.00981 | 0.35334 | 0.09822 | 1.90798 |
|  |  |  |  |  |  |  |  |  |  |  |  |  |
| **Terrier** |  |  |  |  |  |  |  |  |  |  |  |  |
| Airedale | 0.83160 | 1.78595 | 0.00050 | 0.03911 | 2.24699 | 1.12200 | 2.29286 | 1.49134 | 0.54344 | 1.84421 | 0.22701 | 2.31091 |
| Border terrier | 0.90708 | 0.23524 | 1.01575 | 1.01763 | 0.51456 | 0.37591 | 6.67876 | 5.87553 | 3.45552 | 0.59888 | 0.89092 | 5.12545 |
| Cairn terrier | 0.38463 | 0.04485 | 0.60886 | 1.03293 | 0.50948 | 0.12358 | 3.11381 | 2.72589 | 0.44964 | 3.60240 | 0.76445 | 0.40196 |
| Fox terrier | 0.74848 | 0.14695 | 1.65972 | 0.70217 | 0.04741 | 0.10637 | 0.31083 | 0.78851 | 0.04530 | 0.20102 | 0.02759 | 0.51381 |
| Norfolk terrier | 0.03301 | 0.21069 | 0.22500 | 0.37986 | 0.86782 | 0.37986 | 0.00804 | 0.02021 | 0.17203 | 0.12540 | 0.17203 | 1.41121 |
| Scottish terrier | 0.57928 | 0.64301 | 0.86433 | 0.56539 | 1.70797 | 0.44394 | 0.22847 | 0.01580 | 0.98817 | 0.15830 | 0.99835 | 0.28039 |
| West Highland white terrier | 0.54940 | 1.31255 | 0.10824 | 0.08833 | 0.32958 | 0.30620 | 2.99846 | 6.07263 | 5.00537 | 0.01405 | 10.61493 | 15.65758 |
| Yorkshire terrier | 1.25921 | 1.49896 | 2.78252 | 1.70270 | 0.38364 | 1.01066 | 0.63005 | 0.42928 | 1.05854 | 0.32485 | 9.75760 | 1.24744 |
|  |  |  |  |  |  |  |  |  |  |  |  |  |
| **Scent hound** |  |  |  |  |  |  |  |  |  |  |  |  |
| Basset hound | 1.85866 | 0.04031 | 1.00909 | 1.65599 | 0.32026 | 0.37489 | 0.49329 | 0.89838 | 0.34173 | 0.62301 | 0.34173 | 1.61342 |
| Beagle | 0.12646 | 0.19783 | 0.26005 | 0.00433 | 4.94669 | 2.29575 | 0.35309 | 0.30521 | 1.25670 | 1.95156 | 3.41393 | 0.80414 |
| Dachshund | 2.86593 | 1.02456 | 2.83075 | 2.74232 | 1.37316 | 0.00224 | 0.04892 | 0.56249 | 0.32419 | 0.70163 | 0.07098 | 2.75982 |
| Miniature dachshund | 1.67306 | 4.10620 | 0.06627 | 0.68618 | 3.98663 | 1.24457 | 0.28447 | 0.08902 | 0.02873 | 0.04466 | 0.35822 | 1.84970 |
| Rhodesian ridgeback | 2.52089 | 0.65898 | 1.86571 | 2.34722 | 0.48382 | 0.52429 | 0.18296 | 0.18591 | 0.62585 | 0.14956 | 0.28185 | 1.85674 |
|  |  |  |  |  |  |  |  |  |  |  |  |  |
| **Spaniel/Pointer** |  |  |  |  |  |  |  |  |  |  |  |  |
| American cocker spaniel | 0.82988 | 0.23193 | 0.71595 | 0.53677 | 0.07649 | 0.42802 | 3.92993 | 3.20126 | 0.47491 | 1.34227 | 0.56023 | 1.00890 |
| Cavalier King Charles spaniel | 15.65758 | 2.27698 | 15.65758 | 15.65758 | 1.59923 | 1.20472 | 15.10949 | 12.28148 | 0.63747 | 13.67129 | 0.95307 | 1.51547 |
| Cocker spaniel | 3.85081 | 1.14007 | 5.60128 | 4.10628 | 0.55580 | 1.05035 | 6.32001 | 7.14205 | 1.81959 | 0.75888 | 2.31042 | 0.06678 |
| English setter | 0.23399 | 0.02355 | 0.15339 | 0.13768 | 0.42429 | 0.17407 | 1.04475 | 0.95868 | 0.32464 | 0.53853 | 0.06372 | 0.50143 |
| German shorthaired Pointer | 0.10328 | 1.71220 | 0.14433 | 0.03083 | 0.03298 | 0.58067 | 0.17110 | 0.64734 | 0.79152 | 0.28064 | 1.07249 | 0.31767 |
| Gordon setter | 3.04181 | 0.19119 | 1.62009 | 2.16303 | 0.16930 | 0.04855 | 0.17312 | 0.48342 | 0.09843 | 2.70357 | 0.80351 | 0.96145 |
| Hungarian vizsla | 0.88951 | 0.16981 | 0.24514 | 0.75425 | 1.14528 | 0.46122 | 0.28888 | 0.07469 | 0.34790 | 0.62897 | 0.71722 | 0.58131 |
| Irish setter | 1.01077 | 3.31305 | 0.14561 | 0.20118 | 1.05390 | 0.74187 | 0.28529 | 1.75259 | 0.01543 | 0.82538 | 4.51440 | 0.64831 |
| Italian spinone | 0.22421 | 1.88411 | 0.10743 | 0.86408 | 2.71879 | 0.00273 | 0.37600 | 0.30689 | 0.01825 | 1.23953 | 1.76406 | 0.52491 |
| Pointer | 0.45000 | 0.47268 | 0.01217 | 0.50355 | 0.13658 | 0.53590 | 0.16922 | 0.12701 | 0.09400 | 0.45902 | 0.42993 | 0.35076 |
| Springer spaniel | 4.92049 | 1.46692 | 2.67098 | 3.42746 | 1.81082 | 0.21214 | 0.45344 | 1.78677 | 4.64096 | 0.30276 | 2.94973 | 0.75311 |
| Weimaraner | 1.68668 | 5.95578 | 0.05429 | 1.12793 | 15.17644 | 1.12271 | 0.06634 | 0.05583 | 3.81611 | 0.57007 | 0.80672 | 0.49982 |
|  |  |  |  |  |  |  |  |  |  |  |  |  |
| **Other** |  |  |  |  |  |  |  |  |  |  |  |  |
| Bichon frise | 0.64716 | 3.24413 | 0.18801 | 0.13827 | 4.73120 | 1.46016 | 0.89742 | 0.53961 | 0.74776 | 0.12100 | 1.35451 | 5.72996 |
| Dalmatian | 0.39062 | 1.07284 | 0.02823 | 0.33467 | 0.23694 | 1.53884 | 2.27575 | 4.04593 | 0.52818 | 0.04098 | 0.18267 | 0.77279 |
| Jack russell terrier | 0.21579 | 0.20516 | 0.04165 | 0.23494 | 0.47711 | 0.15313 | 2.07098 | 3.97336 | 1.44799 | 0.97118 | 1.55473 | 1.42650 |
| Labradoodle | 0.31611 | 0.35730 | 0.19483 | 0.27238 | 0.78711 | 0.59243 | 0.14020 | 0.19095 | 0.14189 | 2.77259 | 0.36227 | 0.49377 |
| Lhasa apso | 1.90233 | 7.11351 | 0.16157 | 0.01699 | 8.42364 | 0.82565 | 1.33014 | 0.81726 | 0.55356 | 3.99688 | 0.07371 | 0.93749 |
| Miniature poodle | 1.10946 | 0.40809 | 0.58331 | 1.19338 | 0.08005 | 0.44254 | 0.07486 | 0.08663 | 0.42368 | 0.79972 | 1.93254 | 0.04048 |
| Samoyed | 0.28680 | 0.20207 | 0.05636 | 0.76447 | 0.26892 | 0.54156 | 2.62911 | 3.44997 | 1.34314 | 0.58511 | 0.23244 | 0.72916 |
| Standard poodle | 0.70504 | 0.44791 | 0.36984 | 0.84884 | 0.02501 | 0.11808 | 1.84714 | 1.85468 | 1.67727 | 0.07000 | 0.01317 | 0.53229 |
| Toy poodle | 0.06331 | 0.07085 | 0.54961 | 0.25569 | 0.07399 | 0.25288 | 0.79029 | 0.34865 | 0.99115 | 0.08049 | 2.14109 | 0.57989 |
