# Supplementary material for: Breed-Specific Hematological Phenotypes in the Dog: A Natural Resource for the Genetic Dissection of Hematological Parameters in a Mammalian Species
Source: PLoS One. 2013 Nov 25;8(11):e81288. doi: 10.1371/journal.pone.0081288 (PMC3840015; doi:10.1371/journal.pone.0081288)
Supplement: Table S16 — Tentative breed-specific reference intervals for the Labrador retriever (n=761). Abbreviations: RBC, red blood cells; Hb, hemoglobin concentration; Hct, hematocrit; MCV, mean corpuscular volume; MCH, mean corpuscular hemoglobin; WBC, white blood cells; RI, reference interval; F, female; M, male; I, intact; N, neutered; *, undetermined owing to data truncation; §, these values fell below (above) the current lower (upper) RIs because they were calculated lower (upper) limits, i.e. the estimated 2.5% (97.5%) of the residuals plus the adjusted means accounting for age, sex and neutering status for each measurand. (DOC) [file pone.0081288.s031.doc]

| Sex | Age  (years) | RBC  (x10^12^/L) | Hb  (g/dL) | Hct  (%) | MCV  (fL) | MCH  (pg) | WBC  (x10^9^/L) | Neutrophils  (x10^9^/L) | Lymphocytes  (x10^9^/L) | Monocytes  (x10^9^/L) | Eosinophils  (x10^9^/L) | Platelets  (x10^9^/L) |
| --- | --- | --- | --- | --- | --- | --- | --- | --- | --- | --- | --- | --- |
| Current RI | | 5.5 – 8.5 | 12 – 18 | 37 – 55 | 60 – 77 | 19.5 – 24.5 | 6.0 – 17.1 | 3.0 – 11.5 | 1.0 – 4.8 | 0.15 – 1.5 | 0 – 1.3 | 150 – 900 |
| F_I_ | < 1 | 5.4^§^ – 7.4 | 12.6 – 16.9 | 37.5 – 51.9 | 64.3 – 74.8 | 21.5 – * | 7.2 – 14.8 | 3.7 – 10.7 | 1.8 – 4.4 | 0.2 – 1.4 | 0.0 – 1.1 | 150.3 – 454.3 |
|  | > 1 ≤ 2 | 5.7 – 7.6 | 13.3 – 17.6 | 39.4 – 53.8 | 64.5 – 74.9 | 21.6 – * | 6.6 – 14.2 | 3.6 – 10.7 | 1.2 – 3.8 | 0.2 – 1.4 | 0.0 – 1.2 | 131.4^§^ – 435.4 |
|  | > 2 ≤ 8 | 5.7 – 7.7 | 13.4 – 17.7 | 39.8 – 54.2 | 64.5 – 75.0 | 21.7 – * | 5.9 – 13.5 | 3.3 – 10.4 | 0.9^§ –^ 3.5 | 0.1^§^ – 1.3 | 0.0 – 1.1 | 159.3 – 463.3 |
|  | > 8 | 5.6 – 7.6 | 13.1 – 17.4 | 38.7 – 53.1 | 63.9 – 74.4 | 21.5 – * | 6.3 – 13.9 | 3.7 – 10.8 | 0.9^§^ – 3.5 | 0.2 – 1.4 | 0.0 – 1.1 | 223.9 – 527.9 |
| F_N_ | < 1 | 5.7 – 7.6 | 13.2 – 17.5 | 39.0 – 53.4 | 64.0 – 74.4 | 21.6 – * | 6.4 – 14.0 | 3.3 – 10.3 | 1.4 – 4.0 | 0.2 – 1.4 | 0.0 – 1.1 | 100.3^§^ – 404.2 |
|  | > 1 ≤ 2 | 5.7 – 7.6 | 13.5 – 17.8 | 39.7 – 54.1 | 65.0 – 75.4 | 21.9 – * | 6.0 – 13.6 | 3.1 – 10.1 | 1.3 – 3.8 | 0.1^§^ – 1.3 | 0.0 – 1.2 | 109.9^§^ – 413.8 |
|  | > 2 ≤ 8 | 5.7 – 7.6 | 13.4 – 17.7 | 39.7 – 54.1 | 64.5 – 75.0 | 21.7 – * | 6 .0- 13.5 | 3.3 – 10.4 | 1.0 – 3.5 | 0.1^§^ – 1.3 | 0.0 – 1.1 | 142.3^§^ – 446.2 |
|  | > 8 | 5.6 – 7.6 | 13.2 – 17.5 | 39.0 – 53.4 | 64.0 – 74.5 | 21.5 – * | 6.0 – 13.5 | 3.5 – 10.5 | 0.8^§^ – 3.4 | 0.2 – 1.4 | 0.0 – 1.1 | 191.0 – 495.0 |
| M_I_ | < 1 | 5.4^§^ – 7.4 | 12.6 – 17.0 | 37.6 – 52.1 | 64.3 – 74.7 | 21.5 – * | 7.3 – 14.9 | 3.9 – 10.9 | 1.6 – 4.2 | 0.3 – 1.5 | 0.0 – 1.1 | 125.0^§^ – 429.0 |
|  | > 1 ≤ 2 | 5.7 – 7.6 | 13.5 – 17.8 | 39.7 – 54.2 | 64.6 – 75.0 | 21.7 – * | 7.2 – 14.7 | 4.0 – 11.1 | 1.3 – 3.9 | 0.2 – 1.4 | 0.1 – 1.2 | 114.7^§^ – 418.6 |
|  | > 2 ≤ 8 | 5.7 – 7.7 | 13.5 – 17.8 | 39.8 – 54.2 | 64.4 – 74.8 | 21.7 – * | 6.5 – 14.0 | 3.8 – 10.9 | 0.9^§^ – 3.4 | 0.2 – 1.4 | 0.0 – 1.1 | 142.5^§^ – 446.5 |
|  | > 8 | 5.5 – 7.5 | 12.9 – 17.2 | 38.3 – 52.7 | 64.3 – 74.8 | 21.6 – * | 6.5 – 14.1 | 3.9 – 10.9 | 0.9^§^ – 3.4 | 0.3 – 1.5 | 0.0 – 1.1 | 198.9 – 502.8 |
| M_N_ | < 1 | 5.5 – 7.4 | 12.9 – 17.3 | 38.3 – 52.7 | 64.7 – 75.2 | 21.8 – * | 6.9 – 14.5 | 3.4 – 10.5 | 1.6 – 4.2 | 0.2 – 1.4 | 0.1 – 1.2 | 95.3^§^ – 399.2 |
|  | > 1 ≤ 2 | 5.7 – 7.7 | 13.5 – 17.8 | 39.7 – 54.2 | 64.3 – 74.8 | 21.7 – * | 6.4 – 14.0 | 3.3 – 10.3 | 1.3 – 3.9 | 0.1^§^ – 1.3 | 0.1 – 1.2 | 107.2^§^ – 411.2 |
|  | > 2 ≤ 8 | 5.7 – 7.6 | 13.4 – 17.7 | 39.6 – 54.0 | 64.4 – 74.9 | 21.7 – * | 6.1 – 13.7 | 3.4 – 10.5 | 1.0 – 3.6 | 0.1^§^ – 1.3 | 0.0 – 1.1 | 126.1^§^ – 430.0 |
|  | > 8 | 5.6 – 7.5 | 13.0 – 17.4 | 38.5 – 53.0 | 64.2 – 74.7 | 21.6 – * | 6.0 – 13.6 | 3.4 – 10.5 | 0.8^§^ – 3.4 | 0.2 – 1.4 | 0.0 – 1.1 | 187.6 – 491.5 |
