# Supplementary material for: Breed-Specific Hematological Phenotypes in the Dog: A Natural Resource for the Genetic Dissection of Hematological Parameters in a Mammalian Species
Source: PLoS One. 2013 Nov 25;8(11):e81288. doi: 10.1371/journal.pone.0081288 (PMC3840015; doi:10.1371/journal.pone.0081288)
Supplement: Table S17 — Tentative breed-specific reference intervals for the German shepherd dog (n=346). Abbreviations: RBC, red blood cells; Hb, hemoglobin concentration; Hct, hematocrit; MCV, mean corpuscular volume; MCH, mean corpuscular hemoglobin; WBC, white blood cells; RI, reference interval; F, female; M, male; I, intact; N, neutered; *, undetermined owing to data truncation; §, these values fell below (above) the current lower (upper) RIs because they were calculated lower (upper) limits, i.e. the estimated 2.5% (97.5%) of the residuals plus the adjusted means accounting for age, sex and neutering status for each measurand. (DOC) [file pone.0081288.s032.doc]

| Sex | Age  (years) | RBC  (x10^12^/L) | Hb  (g/dL) | Hct  (%) | MCV  (fL) | MCH  (pg) | WBC  (x10^9^/L) | Neutrophils  (x10^9^/L) | Lymphocytes  (x10^9^/L) | Monocytes  (x10^9^/L) | Eosinophils  (x10^9^/L) | Platelets  (x10^9^/L) |
| --- | --- | --- | --- | --- | --- | --- | --- | --- | --- | --- | --- | --- |
| Current RI | | 5.5 – 8.5 | 12 – 18 | 37 – 55 | 60 – 77 | 19.5 – 24.5 | 6.0 – 17.1 | 3.0 – 11.5 | 1.0 – 4.8 | 0.15 – 1.5 | 0 – 1.3 | 150 – 900 |
| F_I_ | < 1 | 5.5 – 7.8 | 12.7 – * | 38.4 – 52.6 | 63.7 – 74.5 | 21.2 – * | 7.4 – 15.2 | 4.0 – 10.8 | 1.6 – 3.6 | 0.3 – 1.5 | 0.0 – 1.3 | 144.5 – 465.5 |
|  | > 1 ≤ 2 | 5.8 – 8 | 13.4 – * | 40.2 – 54.5 | 63.9 – 74.7 | 21.3 – * | 6.8 – 14.6 | 3.9 – 10.8 | 1.0 – 3.0 | 0.2 – 1.4 | 0.1 – 1.3 | 125.6 – 446.6 |
|  | > 2 ≤ 8 | 5.8 – 8.1 | 13.5 – * | 40.6 – 54.9 | 63.9 – 74.7 | 21.4 – * | 6.0 – 13.8 | 3.6 – 10.5 | 0.7^§^ – 2.7 | 0.2 – 1.4 | 0.0 – 1.2 | 153.6 – 474.5 |
|  | > 8 | 5.7 – 8.0 | 13.2 – * | 39.5 – 53.8 | 63.4 – 74.1 | 21.2 – * | 6.4 – 14.3 | 4.0 – 10.9 | 0.7^§^ – 2.7 | 0.2 – 1.4 | 0.0 – 1.2 | 218.1 – 539.1 |
| F_N_ | < 1 | 5.8 – 8.0 | 13.3 – * | 39.9 – 54.1 | 63.4 – 74.2 | 21.3 – * | 6.5 – 14.4 | 3.6 – 10.4 | 1.3 – 3.2 | 0.2 – 1.4 | 0.0 – 1.2 | 94.5^§^ – 415.5 |
|  | > 1 ≤ 2 | 5.8 – 8.0 | 13.6 – * | 40.5 – 54.8 | 64.4 – 75.2 | 21.6 – * | 6.1 – 14.0 | 3.4 – 10.2 | 1.1 – 3.0 | 0.2 – 1.4 | 0.1 – 1.3 | 104.1 – 425.1 |
|  | > 2 ≤ 8 | 5.8 – 8.1 | 13.5 – * | 40.5 – 54.8 | 64.0 – 74.7 | 21.4 – * | 6.1 – 13.9 | 3.7 – 10.5 | 0.8^§^ – 2.7 | 0.2 – 1.4 | 0.0 – 1.2 | 136.5 – 457.5 |
|  | > 8 | 5.8 – 8.0 | 13.3 – * | 39.9 – 54.1 | 63.5 – 74.3 | 21.3 – * | 6.1 – 13.9 | 3.8 – 10.6 | 0.6^§^ – 2.6 | 0.2 – 1.4 | 0.0 – 1.2 | 185.3 – 506.2 |
| M_I_ | < 1 | 5.5 – 7.8 | 12.7 – * | 38.5 – 52.8 | 63.7 – 74.5 | 21.2 – * | 7.4 – 15.3 | 4.2 – 11.0 | 1.4 – 3.4 | 0.3 – 1.5 | 0.0 – 1.2 | 119.3 – 440.2 |
|  | > 1 ≤ 2 | 5.8 – 8.1 | 13.5 – * | 40.6 – 54.9 | 64.0 – 74.8 | 21.5 – * | 7.3 – 15.1 | 4.3 – 11.2 | 1.1 – 3.1 | 0.3 – 1.5 | 0.1 – 1.3 | 108.9 – 429.9 |
|  | > 2 ≤ 8 | 5.8 – 8.1 | 13.6 – * | 40.6 – 54.9 | 63.8 – 74.6 | 21.4 – * | 6.6 – 14.4 | 4.2 – 11.0 | 0.7^§^ – 2.6 | 0.2 – 1.4 | 0.0 – 1.3 | 136.8 – 457.7 |
|  | > 8 | 5.6 – 7.9 | 13.0 – * | 39.1 – 53.4 | 63.8 – 74.5 | 21.3 – * | 6.6 – 14.5 | 4.2 – 11.0 | 0.7^§^ – 2.6 | 0.3 – 1.5 | 0.0 – 1.2 | 193.1 – 514.0 |
| M_N_ | < 1 | 5.6 – 7.8 | 13.0 – * | 39.1 – 53.4 | 64.2 – 74.9 | 21.5 – * | 7.0 – 14.8 | 3.7 – 10.6 | 1.4 – 3.4 | 0.3 – 1.5 | 0.1 – 1.3 | 89.5^§^ – 410.4 |
|  | > 1 ≤ 2 | 5.8 – 8.1 | 13.6 – * | 40.6 – 54.9 | 63.8 – 74.5 | 21.4 – * | 6.5 – 14.3 | 3.6 – 10.4 | 1.2 – 3.1 | 0.2 – 1.4 | 0.1 – 1.3 | 101.5 – 422.4 |
|  | > 2 ≤ 8 | 5.8 – 8.1 | 13.5 – * | 40.4 – 54.7 | 63.8 – 74.6 | 21.4 – * | 6.3 – 14.1 | 3.7 – 10.6 | 0.8^§^ – 2.8 | 0.2 – 1.4 | 0.0 – 1.3 | 120.3 – 441.3 |
|  | > 8 | 5.7 – 7.9 | 13.1 – * | 39.4 – 53.7 | 63.7 – 74.4 | 21.3 – * | 6.1 – 14.0 | 3.8 – 10.6 | 0.7^§^ – 2.6 | 0.2 – 1.4 | 0.0 – 1.3 | 181.8 – 502.8 |
