# Supplementary material for: Breed-Specific Hematological Phenotypes in the Dog: A Natural Resource for the Genetic Dissection of Hematological Parameters in a Mammalian Species
Source: PLoS One. 2013 Nov 25;8(11):e81288. doi: 10.1371/journal.pone.0081288 (PMC3840015; doi:10.1371/journal.pone.0081288)
Supplement: Table S18 — Tentative breed-specific reference intervals for the Cavalier King Charles spaniel (n=280). Abbreviations: RBC, red blood cells; Hb, hemoglobin concentration; Hct, hematocrit; MCV, mean corpuscular volume; MCH, mean corpuscular hemoglobin; WBC, white blood cells; RI, reference interval; F, female; M, male; I, intact; N, neutered; *, undetermined owing to data truncation; §, these values fell below (above) the current lower (upper) RIs because they were calculated lower (upper) limits, i.e. the estimated 2.5% (97.5%) of the residuals plus the adjusted means accounting for age, sex and neutering status for each measurand. (DOC) [file pone.0081288.s033.doc]

| Sex | Age  (years) | RBC  (x10^12^/L) | Hb  (g/dL) | Hct  (%) | MCV  (fL) | MCH  (pg) | WBC  (x10^9^/L) | Neutrophils  (x10^9^/L) | Lymphocytes  (x10^9^/L) | Monocytes  (x10^9^/L) | Eosinophils  (x10^9^/L) | Platelets  (x10^9^/L) |
| --- | --- | --- | --- | --- | --- | --- | --- | --- | --- | --- | --- | --- |
| Current RI | | 5.5 – 8.5 | 12 – 18 | 37 – 55 | 60 – 77 | 19.5 – 24.5 | 6.0 – 17.1 | 3.0 – 11.5 | 1.0 – 4.8 | 0.15 – 1.5 | 0 – 1.3 | 150 – 900 |
| F_I_ | < 1 | 5.3^§^ – 7.0 | 12.2 – 16.4 | 36.6^§^ – 50.2 | 65.9 – 75.4 | 21.7 – * | 7.9 – 16.4 | 4.4 – 11.2 | 1.9 – 4.9^§^ | 0.3 – 1.5 | 0 .0– 1.2 | 135.2^§^ – 589.8 |
|  | > 1 ≤ 2 | 5.5 – 7.3 | 12.9 – 17.1 | 38.4 – 52 | 66.1 – 75.5 | 21.9 – * | 7.3 – 15.8 | 4.4 – 11.2 | 1.3 – 4.3 | 0.2 – 1.4 | 0.1 – 1.2 | 116.3^§^ – 570.9 |
|  | > 2 ≤ 8 | 5.6 – 7.3 | 13.0 – 17.2 | 38.8 – 52.5 | 66.1 – 75.6 | 21.9 – * | 6.6 – 15.1 | 4.1 – 10.9 | 1.0 – 4.0 | 0.2 – 1.4 | 0.0 – 1.1 | 144.2^§^ – 598.9 |
|  | > 8 | 5.5 – 7.2 | 12.7 – 16.9 | 37.7 – 51.4 | 65.6 – 75.0 | 21.7 – * | 7.0 – 15.5 | 4.5 – 11.3 | 1.0 – 4.0 | 0.2 – 1.4 | 0.0 – 1.1 | 208.8 – 663.4 |
| F_N_ | < 1 | 5.5 – 7.2 | 12.8 – 17.0 | 38.0 – 51.7 | 65.6 – 75.0 | 21.8 – * | 7.1 – 15.6 | 4.0 – 10.8 | 1.6 – 4.5 | 0.2 – 1.4 | 0.0 – 1.1 | 85.2^§^ – 539.8 |
|  | > 1 ≤ 2 | 5.5 – 7.2 | 13.1 – 17.3 | 38.7 – 52.4 | 66.6 – 76.0 | 22.2 – * | 6.7 – 15.2 | 3.8 – 10.6 | 1.4 – 4.3 | 0.2 – 1.3 | 0.0 – 1.2 | 94.8^§^ – 549.4 |
|  | > 2 ≤ 8 | 5.6 – 7.3 | 13.0 – 17.2 | 38.7 – 52.3 | 66.2 – 75.6 | 21.9 – * | 6.6 – 15.1 | 4.1 – 10.9 | 1.1 – 4 | 0.2 – 1.3 | 0.0 – 1.1 | 127.2^§^ – 581.8 |
|  | > 8 | 5.5 – 7.2 | 12.8 – 17.0 | 38.1 – 51.7 | 65.7 – 75.1 | 21.8 – * | 6.6 – 15.1 | 4.2 – 11.0 | 0.9^§^ – 3.9 | 0.2 – 1.4 | 0.0 – 1.1 | 175.9 – 630.6 |
| M_I_ | < 1 | 5.3^§^ – 7.0 | 12.3 – 16.4 | 36.7^§^ – 50.3 | 65.9 – 75.3 | 21.7 – * | 8.0 – 16.5 | 4.7 – 11.5 | 1.7 – 4.7 | 0.3 – 1.5 | 0.0 – 1.2 | 109.9^§^ – 564.5 |
|  | > 1 ≤ 2 | 5.6 – 7.3 | 13.1 – 17.2 | 38.8 – 52.4 | 66.2 – 75.6 | 22.0 – * | 7.8 – 16.3 | 4.8 – 11.6^§^ | 1.4 – 4.4 | 0.3 – 1.4 | 0.1 – 1.2 | 99.6^§^ – 554.2 |
|  | > 2 ≤ 8 | 5.6 – 7.3 | 13.1 – 17.3 | 38.8 – 52.5 | 66.0 – 75.4 | 21.9 – * | 7.1 – 15.6 | 4.6 – 11.4 | 1.0 – 3.9 | 0.3 – 1.4 | 0.0 – 1.2 | 127.4 – 582.1 |
|  | > 8 | 5.4^§^ – 7.1 | 12.5 – 16.7 | 37.3 – 50.9 | 66.0 – 75.4 | 21.8 – * | 7.2 – 15.7 | 4.7 – 11.4 | 1.0 – 3.9 | 0.3 – 1.5 | 0.0 – 1.1 | 183.7 – 638.4 |
| M_N_ | < 1 | 5.4^§^ – 7.1 | 12.6 – 16.7 | 37.3 – 50.9 | 66.4 – 75.8 | 22.0 – * | 7.6 – 16.0 | 4.2 – 11.0 | 1.7 – 4.7 | 0.3 – 1.5 | 0.1 – 1.2 | 80.1^§^ – 534.8 |
|  | > 1 ≤ 2 | 5.6 – 7.3 | 13.1 – 17.3 | 38.8 – 52.4 | 66.0 – 75.4 | 21.9 – * | 7.0 – 15.5 | 4.1 – 10.8 | 1.5 – 4.4 | 0.2 – 1.3 | 0.1 – 1.2 | 92.1^§^ – 546.7 |
|  | > 2 ≤ 8 | 5.6 – 7.3 | 13.0 – 17.2 | 38.6 – 52.2 | 66.0 – 75.4 | 21.9 – * | 6.8 – 15.3 | 4.2 – 11.0 | 1.1 – 4.1 | 0.2 – 1.4 | 0.0 – 1.2 | 111.0^§^ – 565.6 |
|  | > 8 | 5.4^§^ – 7.2 | 12.7 – 16.8 | 37.6 – 51.2 | 65.9 – 75.3 | 21.8 – * | 6.7 – 15.2 | 4.2 – 11.0 | 0.9^§^ – 3.9 | 0.2 – 1.4 | 0.0 – 1.2 | 172.5 – 627.1 |
