# Supplementary material for: Breed-Specific Hematological Phenotypes in the Dog: A Natural Resource for the Genetic Dissection of Hematological Parameters in a Mammalian Species
Source: PLoS One. 2013 Nov 25;8(11):e81288. doi: 10.1371/journal.pone.0081288 (PMC3840015; doi:10.1371/journal.pone.0081288)
Supplement: Table S19 — Tentative breed-specific reference intervals for the cocker spaniel (n=227). Abbreviations: RBC, red blood cells; Hb, hemoglobin concentration; Hct, hematocrit; MCV, mean corpuscular volume; MCH, mean corpuscular hemoglobin; WBC, white blood cells; RI, reference interval; F, female; M, male; I, intact; N, neutered; *, undetermined owing to data truncation; §, these values fell below (above) the current lower (upper) RIs because they were calculated lower (upper) limits, i.e. the estimated 2.5% (97.5%) of the residuals plus the adjusted means accounting for age, sex and neutering status for each measurand. (DOC) [file pone.0081288.s034.doc]

| Sex | Age  (years) | RBC  (x10^12^/L) | Hb  (g/dL) | Hct  (%) | MCV  (fL) | MCH  (pg) | WBC  (x10^9^/L) | Neutrophils  (x10^9^/L) | Lymphocytes  (x10^9^/L) | Monocytes  (x10^9^/L) | Eosinophils  (x10^9^/L) | Platelets  (x10^9^/L) |
| --- | --- | --- | --- | --- | --- | --- | --- | --- | --- | --- | --- | --- |
| Current RI | | 5.5 – 8.5 | 12 – 18 | 37 – 55 | 60 – 77 | 19.5 – 24.5 | 6.0 – 17.1 | 3.0 – 11.5 | 1.0 – 4.8 | 0.15 – 1.5 | 0 – 1.3 | 150 – 900 |
| F_I_ | < 1 | 5.3^§^ – 7.5 | 12.5 – 17.1 | 36.9^§^ – 52.6 | 64.3 – 74.6 | 21.5 – * | 7.4 – 15.7 | 4.5 – 11.1 | 1.8 – 4.1 | 0.2 – 1.4 | 0.0 – 1.0 | 161.6 – 542.2 |
|  | > 1 ≤ 2 | 5.6 – 7.8 | 13.2 – 17.8 | 38.7 – 54.4 | 64.5 – 74.8 | 21.7 – * | 6.8 – 15.1 | 4.4 – 11.1 | 1.2 – 3.5 | 0.2 – 1.4 | 0.0 – 1.1 | 142.7 – 523.3 |
|  | > 2 ≤ 8 | 5.6 – 7.8 | 13.3 – 17.9 | 39.1 – 54.9 | 64.5 – 74.8 | 21.7 – * | 6.1 – 14.4 | 4.2 – 10.8 | 0.9^§^ – 3.2 | 0.1^§^ – 1.3 | 0.0 – 1.0 | 170.6 – 551.2 |
|  | > 8 | 5.5 – 7.7 | 13.0 – 17.6 | 38.1 – 53.8 | 63.9 – 74.2 | 21.5 – * | 6.5 – 14.8 | 4.5 – 11.2 | 0.9^§^ – 3.2 | 0.2 – 1.4 | 0.0 – 1.0 | 235.2 – 615.8 |
| F_N_ | < 1 | 5.6 – 7.7 | 13.1 – 17.7 | 38.4 – 54.1 | 64.0 – 74.3 | 21.6 – * | 6.6 – 14.9 | 4.1 – 10.7 | 1.4 – 3.7 | 0.2 – 1.3 | 0.0 – 1.0 | 111.6^§^ – 492.2 |
|  | > 1 ≤ 2 | 5.6 – 7.8 | 13.4 – 17.9 | 39.0 – 54.7 | 65.0 – 75.3 | 22.0 – * | 6.2 – 14.5 | 3.9 – 10.5 | 1.2 – 3.6 | 0.1^§^ – 1.3 | 0.0 – 1.1 | 121.2^§^ – 501.8 |
|  | > 2 ≤ 8 | 5.6 – 7.8 | 13.3 – 17.9 | 39.0 – 54.7 | 64.5 – 74.8 | 21.7 – * | 6.2 – 14.5 | 4.2 – 10.8 | 0.9^§^ – 3.3 | 0.1^§^ – 1.3 | 0.0 – 1.0 | 153.6 – 534.2 |
|  | > 8 | 5.6 – 7.7 | 13.1 – 17.7 | 38.4 – 54.1 | 64.1 – 74.3 | 21.6 – * | 6.2 – 14.4 | 4.3 – 10.9 | 0.8^§^ – 3.1 | 0.2 – 1.3 | 0.0 – 1.0 | 202.3 – 582.9 |
| M_I_ | < 1 | 5.3^§^ – 7.5 | 12.6 – 17.1 | 37.0 – 52.7 | 64.3 – 74.5 | 21.5 – * | 7.5 – 15.8 | 4.7 – 11.4 | 1.6 – 3.9 | 0.3 – 1.4 | 0.0 – 1.0 | 136.3^§^ – 516.9 |
|  | > 1 ≤ 2 | 5.6 – 7.8 | 13.4 – 17.9 | 39.1 – 54.8 | 64.6 – 74.9 | 21.8 – * | 7.4 – 15.6 | 4.8 – 11.5 | 1.3 – 3.6 | 0.2 – 1.4 | 0.1 – 1.1 | 126.0^§^ – 506.6 |
|  | > 2 ≤ 8 | 5.6 – 7.8 | 13.4 – 17.9 | 39.1 – 54.9 | 64.4 – 74.7 | 21.7 – * | 6.7 – 14.9 | 4.7 – 11.3 | 0.8^§^ – 3.2 | 0.2 – 1.4 | 0.0 – 1.0 | 153.8 – 534.4 |
|  | > 8 | 5.4^§^ – 7.6 | 12.8 – 17.4 | 37.6 – 53.3 | 64.3 – 74.6 | 21.6 – * | 6.7 – 15.0 | 4.7 – 11.4 | 0.8^§^ – 3.1 | 0.3 – 1.4 | 0.0 – 1.0 | 210.1 – 590.7 |
| M_N_ | < 1 | 5.4^§^ – 7.6 | 12.9 – 17.4 | 37.6 – 53.3 | 64.8 – 75.0 | 21.8 – * | 7.1 – 15.4 | 4.2 – 10.9 | 1.6 – 3.9 | 0.2 – 1.4 | 0.1 – 1.1 | 106.5^§^ – 487.1 |
|  | > 1 ≤ 2 | 5.6 – 7.8 | 13.4 – 18.0 | 39.1 – 54.8 | 64.3 – 74.6 | 21.7 – * | 6.6 – 14.9 | 4.1 – 10.8 | 1.3 – 3.6 | 0.1^§^ – 1.3 | 0.1 – 1.1 | 118.5^§^ – 499.1 |
|  | > 2 ≤ 8 | 5.6 – 7.8 | 13.3 – 17.9 | 38.9 – 54.6 | 64.4 – 74.7 | 21.7 – * | 6.3 – 14.6 | 4.2 – 10.9 | 1.0 – 3.3 | 0.2 – 1.3 | 0.0 – 1.0 | 137.4^§^ – 518.0 |
|  | > 8 | 5.5 – 7.7 | 13.0 – 17.5 | 37.9 – 53.6 | 64.2 – 74.5 | 21.6 – * | 6.2 – 14.5 | 4.3 – 10.9 | 0.8^§^ – 3.1 | 0.2 – 1.4 | 0.0 – 1.0 | 198.9 – 579.5 |
