# Supplementary material for: Breed-Specific Hematological Phenotypes in the Dog: A Natural Resource for the Genetic Dissection of Hematological Parameters in a Mammalian Species
Source: PLoS One. 2013 Nov 25;8(11):e81288. doi: 10.1371/journal.pone.0081288 (PMC3840015; doi:10.1371/journal.pone.0081288)
Supplement: Table S20 — Tentative breed-specific reference intervals for the West Highland white terrier (n=199). Abbreviations: RBC, red blood cells; Hb, hemoglobin concentration; Hct, hematocrit; MCV, mean corpuscular volume; MCH, mean corpuscular hemoglobin; WBC, white blood cells; RI, reference interval; F, female; M, male; I, intact; N, neutered; *, undetermined owing to data truncation; §, these values fell below (above) the current lower (upper) RIs because they were calculated lower (upper) limits, i.e. the estimated 2.5% (97.5%) of the residuals plus the adjusted means accounting for age, sex and neutering status for each measurand. (DOC) [file pone.0081288.s035.doc]

| Sex | Age  (years) | RBC  (x10^12^/L) | Hb  (g/dL) | Hct  (%) | MCV  (fL) | MCH  (pg) | WBC  (x10^9^/L) | Neutrophils  (x10^9^/L) | Lymphocytes  (x10^9^/L) | Monocytes  (x10^9^/L) | Eosinophils  (x10^9^/L) | Platelets  (x10^9^/L) |
| --- | --- | --- | --- | --- | --- | --- | --- | --- | --- | --- | --- | --- |
| Current RI | | 5.5 – 8.5 | 12 – 18 | 37 – 55 | 60 – 77 | 19.5 – 24.5 | 6.0 – 17.1 | 3.0 – 11.5 | 1.0 – 4.8 | 0.15 – 1.5 | 0 – 1.3 | 150 – 900 |
| F_I_ | < 1 | 5.4^§^ – 7.5 | 12.5 – 17.2 | 38.1 – 52.5 | 64.9 – 75.2 | 21.4 – * | 7.6 – 15.8 | 4.2 – 11.0 | 1.7 – 4.3 | 0.2 – 1.4 | 0.0 – 1.0 | 206.2 – 632.4 |
|  | > 1 ≤ 2 | 5.6 – 7.8 | 13.2 – 17.9 | 40.0 – 54.4 | 65.1 – 75.3 | 21.5 – * | 7.0 – 15.2 | 4.2 – 11.0 | 1.1 – 3.7 | 0.2 – 1.4 | 0.0 – 1.0 | 187.3 – 613.5 |
|  | > 2 ≤ 8 | 5.7 – 7.9 | 13.4 – 18.0 | 40.4 – 54.8 | 65.1 – 75.4 | 21.6 – * | 6.2 – 14.4 | 3.9 – 10.7 | 0.8^§^ – 3.4 | 0.1^§^ – 1.3 | 0.0 – 0.9 | 215.2 – 641.4 |
|  | > 8 | 5.6 – 7.8 | 13.0 – 17.7 | 39.3 – 53.7 | 64.5 – 74.8 | 21.4 – * | 6.7 – 14.9 | 4.3 – 11.1 | 0.8^§^ – 3.4 | 0.2 – 1.4 | 0.0 – 0.9 | 279.8 – 706.0 |
| F_N_ | < 1 | 5.6 – 7.8 | 13.1 – 17.8 | 39.6 – 54.0 | 64.6 – 74.8 | 21.5 – * | 6.8 – 15.0 | 3.8 – 10.6 | 1.3 – 4.0 | 0.2 – 1.4 | 0.0 – 0.9 | 156.2 – 582.4 |
|  | > 1 ≤ 2 | 5.6 – 7.8 | 13.4 – 18.1^§^ | 40.3 – 54.7 | 65.6 – 75.8 | 21.8 – * | 6.4 – 14.5 | 3.6 – 10.4 | 1.1 – 3.8 | 0.1^§^ – 1.3 | 0.0 – 1.0 | 165.8 – 592.0 |
|  | > 2 ≤ 8 | 5.7 – 7.8 | 13.4 – 18.0 | 40.3 – 54.7 | 65.1 – 75.4 | 21.6 – * | 6.3 – 14.5 | 3.9 – 10.7 | 0.8^§^ – 3.5 | 0.1^§^ – 1.3 | 0.0 – 1.0 | 198.2 – 624.3 |
|  | > 8 | 5.6 – 7.8 | 13.1 – 17.8 | 39.6 – 54.0 | 64.7 – 74.9 | 21.5 – * | 6.3 – 14.5 | 4.0 – 10.9 | 0.7^§^ – 3.3 | 0.2 – 1.3 | 0.0 – 0.9 | 246.9 – 673.1 |
| M_I_ | < 1 | 5.4^§^ – 7.6 | 12.6 – 17.3 | 38.2 – 52.7 | 64.9 – 75.1 | 21.4 – * | 7.7 – 15.9 | 4.4 – 11.3 | 1.5 – 4.1 | 0.3 – 1.5 | 0.0 – 1.0 | 180.9 – 607.1 |
|  | > 1 ≤ 2 | 5.7 – 7.8 | 13.4 – 18.1^§^ | 40.3 – 54.8 | 65.2 – 75.4 | 21.6 – * | 7.5 – 15.7 | 4.5 – 11.4 | 1.2 – 3.8 | 0.2 – 1.4 | 0.1 – 1.0 | 170.6 – 596.7 |
|  | > 2 ≤ 8 | 5.7 – 7.9 | 13.4 – 18.1^§^ | 40.4 – 54.8 | 65.0 – 75.2 | 21.6 – * | 6.8 – 15.0 | 4.4 – 11.2 | 0.7^§^ – 3.4 | 0.2 – 1.4 | 0.0 – 1.0 | 198.4 – 624.6 |
|  | > 8 | 5.5 – 7.7 | 12.9 – 17.5 | 38.9 – 53.3 | 64.9 – 75.2 | 21.5 – * | 6.9 – 15.0 | 4.4 – 11.3 | 0.7^§^ – 3.4 | 0.3 – 1.5 | 0.0 – 0.9 | 254.7 – 680.9 |
| M_N_ | < 1 | 5.5 – 7.6 | 12.9 – 17.6 | 38.9 – 53.3 | 65.4 – 75.6 | 21.7 – * | 7.2 – 15.4 | 4.0 – 10.8 | 1.5 – 4.1 | 0.2 – 1.4 | 0.1 – 1.0 | 151.1 – 577.3 |
|  | > 1 ≤ 2 | 5.7 – 7.9 | 13.4 – 18.1^§^ | 40.3 – 54.8 | 64.9 – 75.2 | 21.6 – * | 6.7 – 14.9 | 3.8 – 10.7 | 1.2 – 3.9 | 0.1^§^ – 1.3 | 0.1 – 1.0 | 163.1 – 589.3 |
|  | > 2 ≤ 8 | 5.7 – 7.8 | 13.3 – 18.0 | 40.1 – 54.6 | 65.0 – 75.3 | 21.6 – * | 6.5 – 14.7 | 4.0 – 10.8 | 0.9^§^ – 3.5 | 0.2 – 1.3 | 0.0 – 1.0 | 182.0 – 608.2 |
|  | > 8 | 5.5 – 7.7 | 13.0 – 17.6 | 39.1 – 53.6 | 64.8 – 75.1 | 21.5 – * | 6.4 – 14.6 | 4.0 – 10.8 | 0.7^§^ – 3.4 | 0.2 – 1.4 | 0.0 – 1.0 | 243.5 – 669.7 |
