# Supplementary material for: Breed-Specific Hematological Phenotypes in the Dog: A Natural Resource for the Genetic Dissection of Hematological Parameters in a Mammalian Species
Source: PLoS One. 2013 Nov 25;8(11):e81288. doi: 10.1371/journal.pone.0081288 (PMC3840015; doi:10.1371/journal.pone.0081288)
Supplement: Table S21 — Tentative breed-specific reference intervals for the Jack Russell terrier (n=180). Abbreviations: RBC, red blood cells; Hb, hemoglobin concentration; Hct, hematocrit; MCV, mean corpuscular volume; MCH, mean corpuscular hemoglobin; WBC, white blood cells; RI, reference interval; F, female; M, male; I, intact; N, neutered; *, undetermined owing to data truncation; §, these values fell below (above) the current lower (upper) RIs because they were calculated lower (upper) limits, i.e. the estimated 2.5% (97.5%) of the residuals plus the adjusted means accounting for age, sex and neutering status for each measurand. (DOC) [file pone.0081288.s036.doc]

| Sex | Age  (years) | RBC  (x10^12^/L) | Hb  (g/dL) | Hct  (%) | MCV  (fL) | MCH  (pg) | WBC  (x10^9^/L) | Neutrophils  (x10^9^/L) | Lymphocytes  (x10^9^/L) | Monocytes  (x10^9^/L) | Eosinophils  (x10^9^/L) | Platelets  (x10^9^/L) |
| --- | --- | --- | --- | --- | --- | --- | --- | --- | --- | --- | --- | --- |
| Current RI | | 5.5 – 8.5 | 12 – 18 | 37 – 55 | 60 – 77 | 19.5 – 24.5 | 6.0 – 17.1 | 3.0 – 11.5 | 1.0 – 4.8 | 0.15 – 1.5 | 0 – 1.3 | 150 – 900 |
| F_I_ | < 1 | 5.4^§^ – 7.6 | 12.5 – 17.2 | 37.6 – 52.9 | 63.4 – 75.9 | 20.6 – * | 7.5 – 15.3 | 4.1 – 11.3 | 1.6 – 4.1 | 0.2 – 1.5 | 0.0 – 1.0 | 173.7 – 585.6 |
|  | > 1 ≤ 2 | 5.7 – 7.9 | 13.2 – 17.9 | 39.4 – 54.8 | 63.6 – 76.0 | 20.8 – * | 6.9 – 14.7 | 4.1 – 11.3 | 1.0 – 3.5 | 0.2 – 1.4 | 0.0 – 1.1 | 154.8 – 566.7 |
|  | > 2 ≤ 8 | 5.7 – 7.9 | 13.3 – 18.0 | 39.9 – 55.2 | 63.6 – 76.1 | 20.8 – * | 6.1 – 14.0 | 3.8 – 11.0 | 0.7^§^ – 3.2 | 0.1^§^ – 1.4 | 0.0 – 1.0 | 182.8 – 594.6 |
|  | > 8 | 5.6 – 7.8 | 13.0 – 17.7 | 38.8 – 54.1 | 63.0 – 75.5 | 20.7 – * | 6.6 – 14.4 | 4.2 – 11.4 | 0.7^§^ – 3.2 | 0.2 – 1.4 | 0.0 – 1.0 | 247.3 – 659.2 |
| F_N_ | < 1 | 5.7 – 7.9 | 13.1 – 17.8 | 39.1 – 54.4 | 63.1 – 75.5 | 20.7 – * | 6.7 – 14.5 | 3.7 – 10.9 | 1.3 – 3.7 | 0.2 – 1.4 | 0.0 – 1.0 | 123.7^§^ – 535.6 |
|  | > 1 ≤ 2 | 5.7 – 7.9 | 13.4 – 18.1^§^ | 39.8 – 55.1^§^ | 64.1 – 76.5 | 21.1 – * | 6.2 – 14.1 | 3.5 – 10.7 | 1.1 – 3.5 | 0.1^§^ – 1.4 | 0.0 – 1.0 | 133.3^§^ – 545.2 |
|  | > 2 ≤ 8 | 5.7 – 7.9 | 13.3 – 18.0 | 39.7 – 55.1^§^ | 63.6 – 76.1 | 20.9 – * | 6.2 – 14.0 | 3.8 – 11.0 | 0.8^§^ – 3.3 | 0.1^§^ – 1.4 | 0.0 – 1.0 | 165.7 – 577.6 |
|  | > 8 | 5.7 – 7.9 | 13.1 – 17.8 | 39.1 – 54.4 | 63.2 – 75.6 | 20.7 – * | 6.2 – 14.0 | 3.9 – 11.1 | 0.6^§^ – 3.1 | 0.2 – 1.4 | 0.0 – 1.0 | 214.5 – 626.3 |
| M_I_ | < 1 | 5.4^§^ – 7.7 | 12.5 – 17.3 | 37.7 – 53 | 63.4 – 75.8 | 20.6 – * | 7.6 – 15.4 | 4.4 – 11.6^§^ | 1.4 – 3.9 | 0.3 – 1.5 | 0.0 – 1.0 | 148.5^§^ – 560.3 |
|  | > 1 ≤ 2 | 5.7 – 7.9 | 13.3 – 18.1^§^ | 39.8 – 55.1^§^ | 63.7 – 76.1 | 20.9 – * | 7.4 – 15.2 | 4.5 – 11.7^§^ | 1.1 – 3.6 | 0.2 – 1.5 | 0.1 – 1.1 | 138.1^§^ – 550.0 |
|  | > 2 ≤ 8 | 5.7 – 8.0 | 13.4 – 18.1^§^ | 39.9 – 55.2 | 63.5 – 75.9 | 20.8 – * | 6.7 – 14.5 | 4.3 – 11.5 | 0.7^§^ – 3.1 | 0.2 – 1.5 | 0.0 – 1.0 | 166.0 – 577.8 |
|  | > 8 | 5.5 – 7.7 | 12.8 – 17.5 | 38.3 – 53.7 | 63.4 – 75.9 | 20.8 – * | 6.7 – 14.6 | 4.4 – 11.6^§^ | 0.7^§^ – 3.1 | 0.3 – 1.5 | 0.0 – 1.0 | 222.3 – 634.1 |
| M_N_ | < 1 | 5.5 – 7.7 | 12.8 – 17.6 | 38.3 – 53.7 | 63.9 – 76.3 | 20.9 – * | 7.1 – 15.0 | 3.9 – 11.1 | 1.4 – 3.9 | 0.2 – 1.5 | 0.1 – 1.1 | 118.7^§^ – 530.5 |
|  | > 1 ≤ 2 | 5.7 – 7.9 | 13.4 – 18.1^§^ | 39.8 – 55.1^§^ | 63.5 – 75.9 | 20.9 – * | 6.6 – 14.4 | 3.8 – 11.0 | 1.2 – 3.6 | 0.1^§^ – 1.4 | 0.1 – 1.1 | 130.7^§^ – 542.5 |
|  | > 2 ≤ 8 | 5.7 – 7.9 | 13.3 – 18.0 | 39.6 – 54.9 | 63.5 – 76.0 | 20.9 – * | 6.4 – 14.2 | 3.9 – 11.1 | 0.8^§^ – 3.3 | 0.1^§^ – 1.4 | 0.0 – 1.0 | 149.5^§^ – 561.4 |
|  | > 8 | 5.6 – 7.8 | 12.9 – 17.7 | 38.6 – 53.9 | 63.3 – 75.8 | 20.8 – * | 6.3 – 14.1 | 3.9 – 11.1 | 0.7^§^ – 3.1 | 0.2 – 1.4 | 0.0 – 1.0 | 211.0 – 622.9 |
