# Supplementary material for: Breed-Specific Hematological Phenotypes in the Dog: A Natural Resource for the Genetic Dissection of Hematological Parameters in a Mammalian Species
Source: PLoS One. 2013 Nov 25;8(11):e81288. doi: 10.1371/journal.pone.0081288 (PMC3840015; doi:10.1371/journal.pone.0081288)
Supplement: Table S22 — Tentative breed-specific reference intervals for the golden retriever (n=171). Abbreviations: RBC, red blood cells; Hb, hemoglobin concentration; Hct, hematocrit; MCV, mean corpuscular volume; MCH, mean corpuscular hemoglobin; WBC, white blood cells; RI, reference interval; F, female; M, male; I, intact; N, neutered; *, undetermined owing to data truncation; §, these values fell below (above) the current lower (upper) RIs because they were calculated lower (upper) limits, i.e. the estimated 2.5% (97.5%) of the residuals plus the adjusted means accounting for age, sex and neutering status for each measurand. (DOC) [file pone.0081288.s037.doc]

| Sex | Age  (years) | RBC  (x10^12^/L) | Hb  (g/dL) | Hct  (%) | MCV  (fL) | MCH  (pg) | WBC  (x10^9^/L) | Neutrophils  (x10^9^/L) | Lymphocytes  (x10^9^/L) | Monocytes  (x10^9^/L) | Eosinophils  (x10^9^/L) | Platelets  (x10^9^/L) |
| --- | --- | --- | --- | --- | --- | --- | --- | --- | --- | --- | --- | --- |
| Current RI | | 5.5 – 8.5 | 12 – 18 | 37 – 55 | 60 – 77 | 19.5 – 24.5 | 6.0 – 17.1 | 3.0 – 11.5 | 1.0 – 4.8 | 0.15 – 1.5 | 0 – 1.3 | 150 – 900 |
| F_I_ | < 1 | 5.4^§^ – 7.2 | 12.5 – 16.9 | 37.1 – 51.8 | 65.5 – 75.9 | 21.7 – * | 7.4 – 15.2 | 3.6 – 11.1 | 1.8 – 4.7 | 0.2 – 1.4 | 0.0 – 1.0 | 151.5 – 493.9 |
|  | > 1 ≤ 2 | 5.7 – 7.5 | 13.2 – 17.5 | 39.0 – 53.6 | 65.7 – 76.1 | 21.9 – * | 6.8 – 14.6 | 3.6 – 11.1 | 1.3 – 4.1 | 0.2 – 1.3 | 0.0 – 1.1 | 132.6^§^ – 475.0 |
|  | > 2 ≤ 8 | 5.7 – 7.6 | 13.3 – 17.7 | 39.4 – 54.0 | 65.7 – 76.1 | 21.9 – * | 6.0 – 13.9 | 3.3 – 10.8 | 0.9 – 3.8 | 0.1^§^ – 1.3 | 0.0 – 1.0 | 160.5 – 503.0 |
|  | > 8 | 5.6 – 7.5 | 13.0 – 17.3 | 38.3 – 52.9 | 65.1 – 75.5 | 21.8 – * | 6.5 – 14.3 | 3.7 – 11.2 | 0.9 – 3.8 | 0.2 – 1.3 | 0.0 – 1.0 | 225.1 – 567.5 |
| F_N_ | < 1 | 5.7 – 7.5 | 13.1 – 17.5 | 38.6 – 53.2 | 65.2 – 75.6 | 21.8 – * | 6.6 – 14.4 | 3.2 – 10.7 | 1.5 – 4.4 | 0.2 – 1.3 | 0.0 – 1.0 | 101.5^§^ – 443.9 |
|  | > 1 ≤ 2 | 5.7 – 7.5 | 13.4 – 17.7 | 39.3 – 53.9 | 66.2 – 76.6 | 22.2 – * | 6.2 – 14.0 | 3.0 – 10.5 | 1.3 – 4.2 | 0.1^§^ – 1.3 | 0.0 – 1.1 | 111.1^§^ – 453.5 |
|  | > 2 ≤ 8 | 5.7 – 7.5 | 13.3 – 17.7 | 39.3 – 53.9 | 65.7 – 76.1 | 22.0 – * | 6.1 – 13.9 | 3.3 – 10.8 | 1.0 – 3.9 | 0.1^§^ – 1.3 | 0.0 – 1.0 | 143.5^§^ – 485.9 |
|  | > 8 | 5.7 – 7.5 | 13.1 – 17.4 | 38.6 – 53.2 | 65.2 – 75.7 | 21.8 – * | 6.1 – 13.9 | 3.4 – 10.9 | 0.9^§^ – 3.8 | 0.1^§^ – 1.3 | 0.0 – 1.0 | 192.2 – 534.7 |
| M_I_ | < 1 | 5.4^§^ – 7.3 | 12.5 – 16.9 | 37.3 – 51.9 | 65.5 – 75.9 | 21.7 – * | 7.5 – 15.3 | 3.9 – 11.4 | 1.7 – 4.6 | 0.2 – 1.4 | 0.0 – 1.0 | 126.2^§^ – 468.7 |
|  | > 1 ≤ 2 | 5.7 – 7.5 | 13.3 – 17.7 | 39.4 – 54.0 | 65.8 – 76.2 | 22.0 – * | 7.3 – 15.1 | 4.0 – 11.5 | 1.3 – 4.2 | 0.2 – 1.4 | 0.1 – 1.1 | 115.9^§^ – 458.3 |
|  | > 2 ≤ 8 | 5.7 – 7.6 | 13.4 – 17.7 | 39.4 – 54.0 | 65.6 – 76.0 | 21.9 – * | 6.6 – 14.4 | 3.8 – 11.3 | 0.9^§^ – 3.8 | 0.2 – 1.3 | 0.0 – 1.0 | 143.7^§^ – 486.2 |
|  | > 8 | 5.5 – 7.4 | 12.8 – 17.2 | 37.9 – 52.5 | 65.5 – 75.9 | 21.9 – * | 6.7 – 14.5 | 3.8 – 11.4 | 0.9^§^ – 3.8 | 0.2 – 1.4 | 0.0 – 1.0 | 200.1 – 542.5 |
| M_N_ | < 1 | 5.5 – 7.3 | 12.8 – 17.2 | 37.9 – 52.5 | 65.9 – 76.4 | 22.0 – * | 7.0 – 14.8 | 3.4 – 10.9 | 1.6 – 4.5 | 0.2 – 1.4 | 0.1 – 1.1 | 96.4^§^ – 438.9 |
|  | > 1 ≤ 2 | 5.7 – 7.6 | 13.4 – 17.7 | 39.4 – 54.0 | 65.5 – 75.9 | 22.0 – * | 6.5 – 14.3 | 3.2 – 10.8 | 1.4 – 4.3 | 0.1^§^ – 1.3 | 0.1 – 1.1 | 108.4^§^ – 450.9 |
|  | > 2 ≤ 8 | 5.7 – 7.5 | 13.3 – 17.6 | 39.2 – 53.8 | 65.6 – 76.0 | 22.0 – * | 6.3 – 14.1 | 3.4 – 10.9 | 1.0 – 3.9 | 0.1^§^ – 1.3 | 0.0 – 1.0 | 127.3^§^ – 469.7 |
|  | > 8 | 5.6 – 7.4 | 12.9 – 17.3 | 38.1 – 52.8 | 65.4 – 75.8 | 21.9 – * | 6.2 – 14.0 | 3.4 – 10.9 | 0.9^§^ – 3.8 | 0.2 – 1.3 | 0.0 – 1.0 | 188.8 – 531.2 |
