# Supplementary material for: Breed-Specific Hematological Phenotypes in the Dog: A Natural Resource for the Genetic Dissection of Hematological Parameters in a Mammalian Species
Source: PLoS One. 2013 Nov 25;8(11):e81288. doi: 10.1371/journal.pone.0081288 (PMC3840015; doi:10.1371/journal.pone.0081288)
Supplement: Table S23 — Tentative breed-specific reference intervals for the springer spaniel (n=168). Abbreviations: RBC, red blood cells; Hb, hemoglobin concentration; Hct, hematocrit; MCV, mean corpuscular volume; MCH, mean corpuscular hemoglobin; WBC, white blood cells; RI, reference interval; F, female; M, male; I, intact; N, neutered; *, undetermined owing to data truncation; §, these values fell below (above) the current lower (upper) RIs because they were calculated lower (upper) limits, i.e. the estimated 2.5% (97.5%) of the residuals plus the adjusted means accounting for age, sex and neutering status for each measurand. (DOC) [file pone.0081288.s038.doc]

| Sex | Age  (years) | RBC  (x10^12^/L) | Hb  (g/dL) | Hct  (%) | MCV  (fL) | MCH  (pg) | WBC  (x10^9^/L) | Neutrophils  (x10^9^/L) | Lymphocytes  (x10^9^/L) | Monocytes  (x10^9^/L) | Eosinophils  (x10^9^/L) | Platelets  (x10^9^/L) |
| --- | --- | --- | --- | --- | --- | --- | --- | --- | --- | --- | --- | --- |
| Current RI | | 5.5 – 8.5 | 12 – 18 | 37 – 55 | 60 – 77 | 19.5 – 24.5 | 6.0 – 17.1 | 3.0 – 11.5 | 1.0 – 4.8 | 0.15 – 1.5 | 0 – 1.3 | 150 – 900 |
| F_I_ | < 1 | 5.3^§^ – 7.3 | 12.6 – 17.0 | 36.7^§^ – 51.9 | 65.6 – 76.0 | 22.0 – 24.3 | 6.9 – 15.2 | 3.7 – 11.1 | 1.5 – 4.0 | 0.3 – 1.4 | 0.0 – 1.1 | 175.6 – 520.8 |
|  | > 1 ≤ 2 | 5.6 – 7.6 | 13.2 – 17.7 | 38.5 – 53.8 | 65.7 – 76.2 | 22.1 – 24.4 | 6.3 – 14.6 | 3.7 – 11.1 | 0.9^§^ – 3.4 | 0.2 – 1.4 | 0.0 – 1.2 | 156.7 – 501.9 |
|  | > 2 ≤ 8 | 5.6 – 7.6 | 13.4 – 17.8 | 39.0 – 54.2 | 65.8 – 76.3 | 22.2 – 24.4 | 5.6^§^ – 13.9 | 3.4 – 10.8 | 0.6^§^ – 3.1 | 0.2 – 1.3 | 0.0 – 1.1 | 184.7 – 529.8 |
|  | > 8 | 5.5 – 7.5 | 13.0 – 17.5 | 37.9 – 53.1 | 65.2 – 75.7 | 22.0 – 24.3 | 6.0 – 14.3 | 3.8 – 11.2 | 0.6^§^ – 3.1 | 0.2 – 1.4 | 0.0 – 1.1 | 249.2 – 594.4 |
| F_N_ | < 1 | 5.6 – 7.6 | 13.2 – 17.6 | 38.2 – 53.4 | 65.2 – 75.7 | 22.1 – 24.3 | 6.1 – 14.4 | 3.3 – 10.7 | 1.2 – 3.7 | 0.2 – 1.4 | 0.0 – 1.1 | 125.6^§^ – 470.8 |
|  | > 1 ≤ 2 | 5.6 – 7.6 | 13.4 – 17.9 | 38.8 – 54.1 | 66.2 – 76.7 | 22.4 – 24.7^§^ | 5.7^§^ – 14.0 | 3.1 – 10.5 | 1.0 – 3.5 | 0.1^§^ – 1.3 | 0.0 – 1.2 | 135.2^§^ – 480.4 |
|  | > 2 ≤ 8 | 5.6 – 7.6 | 13.4 – 17.8 | 38.8 – 54.1 | 65.8 – 76.3 | 22.2 – 24.5 | 5.6^§^ – 14.0 | 3.4 – 10.8 | 0.7^§^ – 3.2 | 0.1^§^ – 1.3 | 0.0 – 1.1 | 167.6 – 512.8 |
|  | > 8 | 5.6 – 7.6 | 13.1 – 17.6 | 38.2 – 53.4 | 65.3 – 75.8 | 22.1 – 24.3 | 5.6^§^ – 14.0 | 3.6 – 10.9 | 0. ^§^5 – 3.1 | 0.2 – 1.3 | 0.0 – 1.1 | 216.4 – 561.5 |
| M_I_ | < 1 | 5.4^§^ – 7.3 | 12.6 – 17.1 | 36.8^§^ – 52.0 | 65.5 – 76.0 | 22.0 – 24.3 | 7.0 – 15.3 | 4.0 – 11.4 | 1.3 – 3.8 | 0.3 – 1.5 | 0.0 – 1.1 | 150.4 – 495.5 |
|  | > 1 ≤ 2 | 5.6 – 7.6 | 13.4 – 17.9 | 38.9 – 54.1 | 65.8 – 76.3 | 22.3 – 24.5 | 6.8 – 15.2 | 4.1 – 11.5 | 1.0 – 3.5 | 0.2 – 1.4 | 0.0 – 1.2 | 140.0^§^ – 485.2 |
|  | > 2 ≤ 8 | 5.7 – 7.6 | 13.4 – 17.9 | 39.0 – 54.2 | 65.6 – 76.1 | 22.2 – 24.4 | 6.1 – 14.5 | 3.9 – 11.3 | 0.6^§^ – 3.1 | 0.2 – 1.4 | 0.0 – 1.1 | 167.9 – 513.0 |
|  | > 8 | 5.4^§^ – 7.4 | 12.9 – 17.3 | 37.4 – 52.7 | 65.6 – 76.1 | 22.1 – 24.4 | 6.2 – 14.5 | 4.0 – 11.3 | 0.6^§^ – 3.1 | 0.3 – 1.5 | 0.0 – 1.1 | 224.2 – 569.4 |
| M_N_ | < 1 | 5.4^§^ – 7.4 | 12.9 – 17.4 | 37.4 – 52.7 | 66.0 – 76.5 | 22.3 – 24.6^§^ | 6.6 – 14.9 | 3.5 – 10.9 | 1.3 – 3.8 | 0.3 – 1.4 | 0.0 – 1.2 | 120.6^§^ – 465.7 |
|  | > 1 ≤ 2 | 5.6 – 7.6 | 13.4 – 17.9 | 38.9 – 54.1 | 65.6 – 76.1 | 22.2 – 24.5 | 6.0 – 14.4 | 3.4 – 10.7 | 1.1 – 3.6 | 0.1^§^ – 1.3 | 0.0 – 1.2 | 132.6^§^ – 477.7 |
|  | > 2 ≤ 8 | 5.6 – 7.6 | 13.4 – 17.8 | 38.7 – 53.9 | 65.6 – 76.1 | 22.2 – 24.5 | 5.8^§^ – 14.1 | 3.5 – 10.9 | 0.7^§^ – 3.2 | 0.2 – 1.3 | 0.0 – 1.1 | 151.4 – 496.6 |
|  | > 8 | 5.5 – 7.5 | 13.0 – 17.4 | 37.7 – 52.9 | 65.5 – 76.0 | 22.1 – 24.4 | 5.7^§^ – 14.0 | 3.5 – 10.9 | 0.6^§^ – 3.1 | 0.2 – 1.4 | 0.0 – 1.1 | 212.9 – 558.1 |
