# Supplementary material for: Breed-Specific Hematological Phenotypes in the Dog: A Natural Resource for the Genetic Dissection of Hematological Parameters in a Mammalian Species
Source: PLoS One. 2013 Nov 25;8(11):e81288. doi: 10.1371/journal.pone.0081288 (PMC3840015; doi:10.1371/journal.pone.0081288)
Supplement: Table S24 — Tentative breed-specific reference intervals for the Staffordshire bull terrier (n=165). Abbreviations: RBC, red blood cells; Hb, hemoglobin concentration; Hct, hematocrit; MCV, mean corpuscular volume; MCH, mean corpuscular hemoglobin; WBC, white blood cells; RI, reference interval; F, female; M, male; I, intact; N, neutered; *, undetermined owing to data truncation; §, these values fell below (above) the current lower (upper) RIs because they were calculated lower (upper) limits, i.e. the estimated 2.5% (97.5%) of the residuals plus the adjusted means accounting for age, sex and neutering status for each measurand. (DOC) [file pone.0081288.s039.doc]

| Sex | Age  (years) | RBC  (x10^12^/L) | Hb  (g/dL) | Hct  (%) | MCV  (fL) | MCH  (pg) | WBC  (x10^9^/L) | Neutrophils  (x10^9^/L) | Lymphocytes  (x10^9^/L) | Monocytes  (x10^9^/L) | Eosinophils  (x10^9^/L) | Platelets  (x10^9^/L) |
| --- | --- | --- | --- | --- | --- | --- | --- | --- | --- | --- | --- | --- |
| Current RI | | 5.5 – 8.5 | 12 – 18 | 37 – 55 | 60 – 77 | 19.5 – 24.5 | 6.0 – 17.1 | 3.0 – 11.5 | 1.0 – 4.8 | 0.15 – 1.5 | 0 – 1.3 | 150 – 900 |
| F_I_ | < 1 | 5.6 – 7.6 | 13.0 – * | 38.5 – 52.7 | 64.8 – 74.5 | 21.4 – * | 7.3 – 15.1 | 4 .0– 11.0 | 1.7 – 4.0 | 0.2 – 1.3 | 0.0 – 0.9 | 179.4 – 573.9 |
|  | > 1 ≤ 2 | 5.9 – 7.8 | 13.7 – * | 40.4 – 54.6 | 65.0 – 74.7 | 21.6 – * | 6.7 – 14.5 | 4.0 – 10.9 | 1.2 – 3.4 | 0.2 – 1.2 | 0.0 – 0.9 | 160.5 – 555.0 |
|  | > 2 ≤ 8 | 5.9 – 7.9 | 13.8 – * | 40.8 – 55.0 | 65.0 – 74.7 | 21.6 – * | 5.9^§^ – 13.8 | 3.7 – 10.7 | 0.8^§^ – 3.1 | 0.1^§^ – 1.2 | 0.0 – 0.8 | 188.4 – 582.9 |
|  | > 8 | 5.8 – 7.8 | 13.5 – * | 39.7 – 53.9 | 64.4 – 74.2 | 21.5 – * | 6.4 – 14.2 | 4.1 – 11.0 | 0.8^§^ – 3.1 | 0.2 – 1.2 | 0.0 – 0.8 | 253.0 – 647.5 |
| F_N_ | < 1 | 5.9 – 7.8 | 13.6 – * | 40.0 – 54.2 | 64.5 – 74.2 | 21.5 – * | 6.5 – 14.3 | 3.6 – 10.6 | 1.4 – 3.7 | 0.2 – 1.2 | 0.0 – 0.8 | 129.4^§^ – 523.9 |
|  | > 1 ≤ 2 | 5.9 – 7.8 | 13.9 – * | 40.7 – 54.9 | 65.5 – 75.2 | 21.9 – * | 6.1 – 13.9 | 3.4 – 10.4 | 1.2 – 3.5 | 0.1^§^ – 1.2 | 0.0 – 0.9 | 139.0^§^ – 533.5 |
|  | > 2 ≤ 8 | 5.9 – 7.9 | 13.8 – * | 40.7 – 54.9 | 65.0 – 74.8 | 21.7 – * | 6.0 – 13.9 | 3.7 – 10.7 | 0.9^§^ – 3.2 | 0.1^§^ – 1.2 | 0.0 – 0.8 | 171.4 – 565.9 |
|  | > 8 | 5.8 – 7.8 | 13.6 – * | 40.0 – 54.2 | 64.6 – 74.3 | 21.5 – * | 6.0 – 13.8 | 3.8 – 10.8 | 0.8^§^ – 3.1 | 0.1^§^ – 1.2 | 0.0 – 0.8 | 220.1 – 614.6 |
| M_I_ | < 1 | 5.6 – 7.6 | 13.0 – * | 38.7 – 52.9 | 64.8 – 74.5 | 21.4 – * | 7.4 – 15.2 | 4.3 – 11.2 | 1.6 – 3.9 | 0.2 – 1.3 | 0.0 – 0.8 | 154.1 – 548.6 |
|  | > 1 ≤ 2 | 5.9 – 7.9 | 13.9 – * | 40.8 – 55.0 | 65.1 – 74.8 | 21.7 – * | 7.2 – 15.1 | 4.4 – 11.3 | 1.2 – 3.5 | 0.2 – 1.2 | 0.1 – 0.9 | 143.8^§^ – 538.3 |
|  | > 2 ≤ 8 | 5.9 – 7.9 | 13.9 – * | 40.8 – 55.0 | 64.9 – 74.6 | 21.6 – * | 6.5 – 14.3 | 4.2 – 11.2 | 0.8^§^ – 3.1 | 0.2 – 1.2 | 0.0 – 0.9 | 171.6 – 566.1 |
|  | > 8 | 5.7 – 7.7 | 13.3 – * | 39.3 – 53.5 | 64.8 – 74.6 | 21.5 – * | 6.6 – 14.4 | 4.2 – 11.2 | 0.8^§^ – 3.1 | 0.2 – 1.3 | 0.0 – 0.8 | 227.9 – 622.4 |
| M_N_ | < 1 | 5.7 – 7.7 | 13.3 – * | 39.3 – 53.5 | 65.3 – 75.0 | 21.7 – * | 6.9 – 14.8 | 3.8 – 10.7 | 1.5 – 3.8 | 0.2 – 1.3 | 0.1 – 0.9 | 124.3^§^ – 518.8 |
|  | > 1 ≤ 2 | 5.9 – 7.9 | 13.9 – * | 40.8 – 55.0 | 64.8 – 74.6 | 21.7 – * | 6.4 – 14.3 | 3.6 – 10.6 | 1.3 – 3.6 | 0.1^§^ – 1.2 | 0.1 – 0.9 | 136.3^§^ – 530.8 |
|  | > 2 ≤ 8 | 5.9 – 7.9 | 13.8 – * | 40.6 – 54.8 | 64.9 – 74.6 | 21.7 – * | 6.2 – 14.0 | 3.8 – 10.8 | 1.0 – 3.2 | 0.1^§^ – 1.2 | 0.0 – 0.9 | 155.2 – 549.7 |
|  | > 8 | 5.8 – 7.7 | 13.4 – * | 39.6 – 53.8 | 64.7 – 74.5 | 21.6 – * | 6.1 – 13.9 | 3.8 – 10.8 | 0.8^§^ – 3.1 | 0.2 – 1.2 | 0.0 – 0.9 | 216.7 – 611.2 |
