# Supplementary material for: Breed-Specific Hematological Phenotypes in the Dog: A Natural Resource for the Genetic Dissection of Hematological Parameters in a Mammalian Species
Source: PLoS One. 2013 Nov 25;8(11):e81288. doi: 10.1371/journal.pone.0081288 (PMC3840015; doi:10.1371/journal.pone.0081288)
Supplement: Table S25 — Tentative breed-specific reference intervals for the Yorkshire terrier (n=154). Abbreviations: RBC, red blood cells; Hb, hemoglobin concentration; Hct, hematocrit; MCV, mean corpuscular volume; MCH, mean corpuscular hemoglobin; WBC, white blood cells; RI, reference interval; F, female; M, male; I, intact; N, neutered; *, undetermined owing to data truncation; §, these values fell below (above) the current lower (upper) RIs because they were calculated lower (upper) limits, i.e. the estimated 2.5% (97.5%) of the residuals plus the adjusted means accounting for age, sex and neutering status for each measurand. (DOC) [file pone.0081288.s040.doc]

| Sex | Age  (years) | RBC  (x10^12^/L) | Hb  (g/dL) | Hct  (%) | MCV  (fL) | MCH  (pg) | WBC  (x10^9^/L) | Neutrophils  (x10^9^/L) | Lymphocytes  (x10^9^/L) | Monocytes  (x10^9^/L) | Eosinophils  (x10^9^/L) | Platelets  (x10^9^/L) |
| --- | --- | --- | --- | --- | --- | --- | --- | --- | --- | --- | --- | --- |
| Current RI | | 5.5 – 8.5 | 12 – 18 | 37 – 55 | 60 – 77 | 19.5 – 24.5 | 6.0 – 17.1 | 3.0 – 11.5 | 1.0 – 4.8 | 0.15 – 1.5 | 0 – 1.3 | 150 – 900 |
| F_I_ | < 1 | 5.6 – 7.6 | 12.6 – 17.2 | 38.3 – 52.9 | 63.8 – 75.3 | 21.1 – 24.3 | 7.2 – 15.1 | 3.6 – 11.2 | 1.6 – 4.5 | 0.2 – 1.4 | 0.0 – 0.8 | 159.4 – 618.2 |
|  | > 1 ≤ 2 | 5.8 – 7.8 | 13.3 – 17.9 | 40.1 – 54.8 | 64.0 – 75.5 | 21.2 – 24.5 | 6.6 – 14.5 | 3.5 – 11.1 | 1.1 – 3.9 | 0.2 – 1.4 | 0.0 – 0.9 | 140.5^§^ – 599.3 |
|  | > 2 ≤ 8 | 5.9 – 7.9 | 13.4 – 18.0 | 40.5 – 55.2 | 64.1 – 75.5 | 21.3 – 24.5 | 5.8 – 13.7 | 3.3 – 10.9 | 0.7^§^ – 3.6 | 0.1 – 1.3 | 0.0 – 0.8 | 168.4 – 627.2 |
|  | > 8 | 5.8 – 7.8 | 13.1 – 17.7 | 39.4 – 54.1 | 63.5 – 74.9 | 21.1 – 24.4 | 6.3 – 14.2 | 3.6 – 11.2 | 0.8^§^ – 3.6 | 0.2 – 1.4 | 0.0 – 0.8 | 233.0 – 691.8 |
| F_N_ | < 1 | 5.8 – 7.8 | 13.2 – 17.8 | 39.8 – 54.4 | 63.5 – 75.0 | 21.2 – 24.4 | 6.4 – 14.3 | 3.2 – 10.8 | 1.3 – 4.2 | 0.2 – 1.4 | 0.0 – 0.8 | 109.4^§^ – 568.2 |
|  | > 1 ≤ 2 | 5.8 – 7.8 | 13.5 – 18.0 | 40.4 – 55.1 | 64.5 – 76.0 | 21.5 – 24.8^§^ | 6.0 – 13.9 | 3.0 – 10.6 | 1.1 – 4.0 | 0.1^§^ – 1.3 | 0.0 – 0.9 | 119.0^§^ – 577.8 |
|  | > 2 ≤ 8 | 5.9 – 7.9 | 13.4 – 18.0 | 40.4 – 55.1 | 64.1 – 75.5 | 21.3 – 24.6^§^ | 5.9 – 13.8 | 3.3 – 10.9 | 0.8^§^ – 3.7 | 0.1^§^ – 1.3 | 0.0 – 0.8 | 151.4 – 610.2 |
|  | > 8 | 5.8 – 7.8 | 13.2 – 17.8 | 39.8 – 54.4 | 63.6 – 75.1 | 21.2 – 24.4 | 5.9 – 13.8 | 3.4 – 11.0 | 0.7^§^ – 3.5 | 0.2 – 1.4 | 0.0 – 0.8 | 200.1 – 658.9 |
| M_I_ | < 1 | 5.6 – 7.6 | 12.7 – 17.2 | 38.4 – 53 | 63.8 – 75.3 | 21.1 – 24.3 | 7.3 – 15.2 | 3.8 – 11.4 | 1.5 – 4.3 | 0.3 – 1.5 | 0.0 – 0.8 | 134.1^§^ – 592.9 |
|  | > 1 ≤ 2 | 5.9 – 7.9 | 13.5 – 18.0 | 40.5 – 55.1 | 64.1 – 75.6 | 21.3 – 24.6^§^ | 7.1 – 15.0 | 3.9 – 11.5 | 1.1 – 4.0 | 0.2 – 1.4 | 0.0 – 0.9 | 123.8^§^ – 582.6 |
|  | > 2 ≤ 8 | 5.9 – 7.9 | 13.5 – 18.0 | 40.5 – 55.2 | 63.9 – 75.4 | 21.3 – 24.5 | 6.4 – 14.3 | 3.8 – 11.4 | 0.7^§^ – 3.6 | 0.2 – 1.4 | 0.0 – 0.8 | 151.6 – 610.4 |
|  | > 8 | 5.7 – 7.7 | 12.9 – 17.5 | 39.0 – 53.7 | 63.9 – 75.3 | 21.2 – 24.5 | 6.5 – 14.4 | 3.8 – 11.4 | 0.7^§^ – 3.6 | 0.3 – 1.5 | 0.0 – 0.8 | 207.9 – 666.7 |
| M_N_ | < 1 | 5.6 – 7.6 | 13.0 – 17.5 | 39.0 – 53.7 | 64.3 – 75.8 | 21.4 – 24.6^§^ | 6.8 – 14.7 | 3.3 – 10.9 | 1.5 – 4.3 | 0.2 – 1.4 | 0.0 – 0.9 | 104.3^§^ – 563.1 |
|  | > 1 ≤ 2 | 5.9 – 7.9 | 13.5 – 18.1^§^ | 40.5 – 55.1 | 63.9 – 75.3 | 21.3 – 24.6^§^ | 6.3 – 14.2 | 3.2 – 10.8 | 1.2 – 4.1 | 0.1^§^ – 1.3 | 0.0 – 0.9 | 116.3^§^ – 575.1 |
|  | > 2 ≤ 8 | 5.9 – 7.9 | 13.4 – 18.0 | 40.3 – 54.9 | 63.9 – 75.4 | 21.3 – 24.6^§^ | 6.1 – 14.0 | 3.3 – 10.9 | 0.9^§^ – 3.7 | 0.1^§^ – 1.3 | 0.0 – 0.8 | 135.2^§^ – 594.0 |
|  | > 8 | 5.7 – 7.7 | 13.1 – 17.6 | 39.3 – 53.9 | 63.8 – 75.2 | 21.2 – 24.5 | 6.0 – 13.9 | 3.4 – 11.0 | 0.7^§^ – 3.6 | 0.2 – 1.4 | 0.0 – 0.8 | 196.7 – 655.5 |
