# Supplementary material for: Breed-Specific Hematological Phenotypes in the Dog: A Natural Resource for the Genetic Dissection of Hematological Parameters in a Mammalian Species
Source: PLoS One. 2013 Nov 25;8(11):e81288. doi: 10.1371/journal.pone.0081288 (PMC3840015; doi:10.1371/journal.pone.0081288)
Supplement: Table S26 — Tentative breed-specific reference intervals for the Rottweiler (n=128). Abbreviations: RBC, red blood cells; Hb, hemoglobin concentration; Hct, hematocrit; MCV, mean corpuscular volume; MCH, mean corpuscular hemoglobin; WBC, white blood cells; RI, reference interval; F, female; M, male; I, intact; N, neutered; *, undetermined owing to data truncation; §, these values fell below (above) the current lower (upper) RIs because they were calculated lower (upper) limits, i.e. the estimated 2.5% (97.5%) of the residuals plus the adjusted means accounting for age, sex and neutering status for each measurand. (DOC) [file pone.0081288.s041.doc]

| Sex | Age  (years) | RBC  (x10^12^/L) | Hb  (g/dL) | Hct  (%) | MCV  (fL) | MCH  (pg) | WBC  (x10^9^/L) | Neutrophils  (x10^9^/L) | Lymphocytes  (x10^9^/L) | Monocytes  (x10^9^/L) | Eosinophils  (x10^9^/L) | Platelets  (x10^9^/L) |
| --- | --- | --- | --- | --- | --- | --- | --- | --- | --- | --- | --- | --- |
| Current RI | | 5.5 – 8.5 | 12 - 18 | 37 - 55 | 60 - 77 | 19.5 – 24.5 | 6.0 – 17.1 | 3.0 – 11.5 | 1.0 – 4.8 | 0.15 – 1.5 | 0 – 1.3 | 150 - 900 |
| F_I_ | < 1 | 5.3^§^ – 7.5 | 11.9^§^ – 17 | 36.4^§^ – 51.6 | 63.6 – 73.9 | 21.0 – * | 7.4 – 16.1 | 3.7 – 11.3 | 1.6 – 4.3 | 0.3 – 1.5 | 0.0 – 1.3 | 211.5 – 589.7 |
|  | > 1 ≤ 2 | 5.5 – 7.8 | 12.6 – 17.6 | 38.3 – 53.4 | 63.7 – 74.1 | 21.1 – * | 6.8 – 15.5 | 3.6 – 11.3 | 1.0 – 3.7 | 0.2 – 1.5 | 0.1 – 1.3 | 192.6 – 570.8 |
|  | > 2 ≤ 8 | 5.6 – 7.8 | 12.8 – 17.8 | 38.7 – 53.8 | 63.8 – 74.1 | 21.2 – * | 6.0 – 14.7 | 3.4 – 11.0 | 0.7^§^ – 3.4 | 0.2 – 1.4 | 0.0 – 1.2 | 220.6 – 598.7 |
|  | > 8 | 5.5 – 7.7 | 12.4 – 17.4 | 37.6 – 52.8 | 63.2 – 73.6 | 21.0 – * | 6.5 – 15.2 | 3.7 – 11.4 | 0.7^§^ – 3.4 | 0.2 – 1.5 | 0.0 – 1.2 | 285.1 – 663.3 |
| F_N_ | < 1 | 5.5 – 7.8 | 12.6 – 17.6 | 37.9 – 53.1 | 63.2 – 73.6 | 21.1 – * | 6.5 – 15.2 | 3.3 – 10.9 | 1.2 – 4.0 | 0.2 – 1.4 | 0.0 – 1.2 | 161.5 – 539.7 |
|  | > 1 ≤ 2 | 5.5 – 7.8 | 12.8 – 17.8 | 38.6 – 53.7 | 64.2 – 74.6 | 21.4 – * | 6.1 – 14.8 | 3.1 – 10.7 | 1.1 – 3.8 | 0.2 – 1.4 | 0.1 – 1.3 | 171.1 – 549.3 |
|  | > 2 ≤ 8 | 5.6 – 7.8 | 12.8 – 17.8 | 38.6 – 53.7 | 63.8 – 74.2 | 21.2 – * | 6.1 – 14.8 | 3.4 – 11.0 | 0.8^§^ – 3.5 | 0.1^§^ – 1.4 | 0.0 – 1.2 | 203.5 – 581.6 |
|  | > 8 | 5.5 – 7.8 | 12.5 – 17.5 | 37.9 – 53.1 | 63.3 – 73.7 | 21.0 – * | 6.1 – 14.8 | 3.5 – 11.2 | 0.6^§^ – 3.3 | 0.2 – 1.4 | 0.0 – 1.2 | 252.3 – 630.4 |
| M_I_ | < 1 | 5.3^§^ – 7.5 | 12.0 – 17.0 | 36.6^§^ – 51.7 | 63.5 – 73.9 | 21.0 – * | 7.4 – 16.1 | 3.9 – 11.6^§^ | 1.4 – 4.1 | 0.3 – 1.5 | 0.0 – 1.3 | 186.2 – 564.4 |
|  | > 1 ≤ 2 | 5.6 – 7.8 | 12.8 – 17.8 | 38.7 – 53.8 | 63.8 – 74.2 | 21.2 – * | 7.3 – 16.0 | 4.0 – 11.7^§^ | 1.1 – 3.8 | 0.2 – 1.5 | 0.1 – 1.3 | 175.9 – 554.0 |
|  | > 2 ≤ 8 | 5.6 – 7.8 | 12.8 – 17.8 | 38.7 – 53.8 | 63.6 – 74.0 | 21.2 – * | 6.6 – 15.3 | 3.9 – 11.5 | 0.7^§^ – 3.4 | 0.2 – 1.5 | 0.0 – 1.3 | 203.8 – 581.9 |
|  | > 8 | 5.4^§^ – 7.6 | 12.3 – 17.3 | 37.2 – 52.3 | 63.6 – 74.0 | 21.1 – * | 6.6 – 15.3 | 3.9 – 11.6^§^ | 0.7^§^ – 3.4 | 0.3 – 1.5 | 0.0 – 1.2 | 260.1 – 638.2 |
| M_N_ | < 1 | 5.3^§^ – 7.6 | 12.3 – 17.3 | 37.2 – 52.3 | 64.0 – 74.4 | 21.3 – * | 7.0 – 15.7 | 3.4 – 11.1 | 1.4 – 4.1 | 0.3 – 1.5 | 0.1 – 1.3 | 156.5 – 534.6 |
|  | > 1 ≤ 2 | 5.6 – 7.8 | 12.8 – 17.8 | 38.7 – 53.8 | 63.6 – 74.0 | 21.2 – * | 6.5 – 15.2 | 3.3 – 11.0 | 1.1 – 3.8 | 0.2 – 1.4 | 0.1 – 1.3 | 168.4 – 546.6 |
|  | > 2 ≤ 8 | 5.6 – 7.8 | 12.7 – 17.8 | 38.5 – 53.6 | 63.6 – 74.0 | 21.2 – * | 6.3 – 15.0 | 3.4 – 11.1 | 0.8^§^ – 3.5 | 0.2 – 1.4 | 0.0 – 1.3 | 187.3 – 565.5 |
|  | > 8 | 5.4^§^ – 7.7 | 12.4 – 17.4 | 37.4 – 52.6 | 63.5 – 73.9 | 21.1 – * | 6.1 – 14.8 | 3.5 – 11.1 | 0.6^§^ – 3.3 | 0.2 – 1.4 | 0.0 – 1.3 | 248.8 – 627.0 |
