# Supplementary material for: Breed-Specific Hematological Phenotypes in the Dog: A Natural Resource for the Genetic Dissection of Hematological Parameters in a Mammalian Species
Source: PLoS One. 2013 Nov 25;8(11):e81288. doi: 10.1371/journal.pone.0081288 (PMC3840015; doi:10.1371/journal.pone.0081288)
Supplement: Materials and Methods S1 — (DOC) [file pone.0081288.s042.doc]

**Materials and Methods** (detailed version)

*Inclusion and exclusion criteria*

Hematological profiles were collected from the database of the Royal Veterinary College (RVC) Diagnostic Laboratory (DL) from December 1998 to August 2012, which receives samples from both its own small animal hospitals (the Queen Mother Hospital for Animals, a primary, secondary and tertiary care center, and the Beaumont Sainsbury Animal Hospital, a primary care center) and external veterinary practices. The database was interrogated over the period of time from its inception in December 1998 to August 2012, comparing each dog’s profile to the generic reference intervals used by the DL for all mixed and pure breed dogs other than greyhounds, derived by validation of the most widely used veterinary hematology reference intervals [1] by sampling approximately 40 healthy staff dogs of various breeds (excluding greyhounds), ages, sexes and neutering status. In the case of greyhounds, comparisons were made to widely-applied greyhound-specific reference intervals used by the DL over this period. Hematological profiles were included in the study if every parameter fell within the reference interval, excluding platelet concentrations, which were interpreted separately. In the case of platelets, we recognized that clumping falsely decreases the concentration measured by the automated analyzer, which could potentially mask thrombocytosis or lead to spurious thrombocytopenia. Platelet concentrations falling outside the reference interval were therefore not considered to be an exclusion criterion for the interpretation of the other hematological parameters, but only those concentrations falling within the reference interval were considered in the analysis of platelet data. If more than one normal hematological profile was discovered from the same dog, the earliest recorded profile was captured for the purposes of this study. Profiles with missing data values or samples that were hemolyzed or lipemic were excluded from analysis, as were those associated with insufficiently specific breed descriptors or missing signalment information. Data were collected from both pure breed dogs and a control group of mixed breed dogs, anonymizing all patient details in accordance with general guidelines laid down by the Royal Veterinary College Ethics and Welfare Committee.

*Specimen collection, analysis, and quality control*

All blood samples were collected by licensed veterinarians for routine diagnostic purposes under the Veterinary Surgeons Act (1966), following written informed consent by the owners of the dogs. Whole blood was collected from the jugular, cephalic or lateral saphenous vein of each dog into tubes containing potassium (K_2_)-EDTA and analyzed within 48 hours of collection. All blood samples were analyzed by multi-angle polarized scatter separation and impedance using a CELL-DYN 3500 Hematology Analyzer (Abbott Laboratories Ltd, Maidenhead; UK), adopting veterinary species-specific software settings. Blood smears were evaluated under the direct supervision of a Board-certified veterinary clinical pathologist in every case. Reported parameters were red blood cell (RBC) concentration, mean corpuscular volume (MCV), hematocrit (Hct), hemoglobin (Hb) concentration, mean corpuscular haemoglobin (MCH), mean corpuscular hemoglobin concentration (MCHC), and concentrations of white blood cells (WBCs), neutrophils, monocytes, lymphocytes, eosinophils, and platelets. In the case of WBCs, at least a 100-cell manual differential count was undertaken as a basis for the calculation of the concentrations of neutrophils, monocytes, lymphocytes and eosinophils.

Internal quality control (IQC) comprised three levels of control material (normal, low and high; Abbott Laboratories Ltd) run each day before patient samples were analyzed. Results were accepted only when control samples fell within the mean range provided by the manufacturer and IQC daily print-outs were archived for at least 10 years. The laboratory also participated in an external hematology quality assurance program provided monthly by the United Kingdom National External Quality Assessment Scheme (NEQUAS).

*Statistical analysis*

Age of the dogs was classified into eight mutually exclusive categories, namely less than or equal to one year of age (designated ≤1), greater than one year to less than or equal to two years of age (designated >1:≤2), and >2:≤4, >4:≤6, >6:≤8, >8:≤10, >10:≤12, and >12 years of age. (For clarity of expression in the text of the Results and Discussion sections, these age categories are respectively labeled in abbreviated form: ≤1, 1‒2, 2‒4, 4‒6, 6‒8, 8‒10, 10‒12 and >12 years.) A linear mixed effects model was used to assess the effect of age, sex, neutering status, and all two-way and three-way interactions on each parameter, taking breed as a random effect. Significance was assumed when p<0.05. There were no significant interactions between age and sex, or between age, sex and neutering status, for any of the measured parameters; these interactions were therefore removed from the linear mixed effects model. Residuals were defined as the observed values minus the estimated fixed effects of age, sex, and neutering status. For those breeds represented by at least 10 dogs, principal component analysis (PCA) by best linear unbiased prediction of the random breed effects was undertaken on all but the mixed breed dogs to summarize correlated breed effects on hematological parameters. Those breeds with at least one principal component of less than -2 or greater than +2 were considered to have distinctive phenotypes.

A complementary analytical approach was then adopted to verify the conclusions of PCA. For each hematological parameter, the distributions of residuals for all breeds represented by at least 10 dogs were compared with those of the mixed breed dogs by two-sample Kolmogorov-Smirnov (KS) tests. Since there were 75 breeds represented by at least 10 dogs, a Bonferroni correction of 0.05/75=6.7 x 10^-4^ was applied. In an effort to increase the rigor of the statistical analysis and further minimize the possibility of a type 1 error inherent in multiple comparisons, a threshold of p=10^-4^ was applied in these analyses. To facilitate the pictorial representation of the data analyzed by KS tests, all breeds were classified into groups previously defined by haplotype analysis of single nucleotide polymorphisms (SNPs) [2], or to their closest matches based on publically available information about breed heritage. These groups were: Ancient, Toy, Working, Sight Hound, Mastiff-like, Retriever/other Mastiff-like, Herding, Terrier, Scent Hound and Spaniel/Pointer (Supplementary Tables 1 to 12). Those breeds not identified in this haplotype analysis and without an obvious nearest match were considered in a miscellaneous group labeled ‘Other’. Data from breeds having values significantly different from the mixed breed control group were displayed in combined box-and-whisker / dot plots.

Where significant differences were identified, tentative breed-specific reference intervals were calculated. Within a specific breed the hematological parameters were assumed to differ with age, sex and neutering status, without concomitant differences in variation. Linear mixed effects models were used to estimate adjusted means of age (≤1, >1:≤2, >2:≤8, >8), sex and neutering status using all available data. The Harrell-Davis distribution-free quantile estimator was adopted to estimate the 2.5 and 97.5 percentiles (± jacknife standard errors) of the residuals for breeds with at least 120 individuals. Tentative breed-specific reference intervals were calculated as the estimated 2.5% and 97.5% of the residuals, plus the appropriate adjusted means accounting for age, sex and neutering status. The upper limit for the 95% breed-specific reference interval per parameter was not calculated if more than 2% of the recorded data were equal to the existing RVC DL upper reference limit, thus suggesting ‘truncation’ of the data. Truncation in this manner was either not apparent for the lower limits of any of the parameters, or the lower limit was already 0.

**References for supporting information**

1. Lumsden JH, Mullen K, McSherry BJ (1979) Canine hematology and biochemistry reference values. Can J Comp Med 43: 125-131.

2. Vonholdt BM, Pollinger JP, Lohmueller KE, Han E, Parker HG, et al. (2010) Genome-wide SNP and haplotype analyses reveal a rich history underlying dog domestication. Nature 464: 898-902.
